# Click Pt(IV)-Carbohydrates Pro-Drugs for Treatment of Osteosarcoma

**DOI:** 10.3389/fchem.2021.795997

**Published:** 2021-12-07

**Authors:** Eoin Moynihan, Giada Bassi, Andrea Ruffini, Silvia Panseri, Monica Montesi, Trinidad Velasco-Torrijos, Diego Montagner

**Affiliations:** ^1^ Department of Chemistry, Maynooth University, Maynooth, Ireland; ^2^ Institute of Science and Technology for Ceramics—National Research Council, Faenza, Italy; ^3^ Kathleen Londsdale Institute for Human Health Research, Maynooth University, Maynooth, Ireland

**Keywords:** Pt(IV) prodrugs, cisplatin, sugars, osteosarcoma, cancer stem cells, click chemistry

## Abstract

The selectivity *vs.* cancer cells has always been a major challenge for chemotherapeutic agents and in particular for cisplatin, one of the most important anticancer drugs for the treatment of several types of tumors. One strategy to overtake this challenge is to modify the coordination sphere of the metallic center with specific vectors whose receptors are overexpressed in the tumoral cell membrane, such as monosaccharides. In this paper, we report the synthesis of four novel glyco-modified Pt(IV) pro-drugs, based on cisplatin scaffold, and their biological activity against osteosarcoma (OS), a malignant tumor affecting in particular adolescents and young adults. The sugar moiety and the Pt scaffold are linked exploiting the Copper Azide Alkyne Cycloaddition (CUAAC) reaction, which has become the flagship of click chemistry due to its versatility and mild conditions. Cytotoxicity and drug uptake on three different OS cell lines as well as CSCs (Cancer Stem Cell) are described.

## Introduction

**FIGURE 1:**
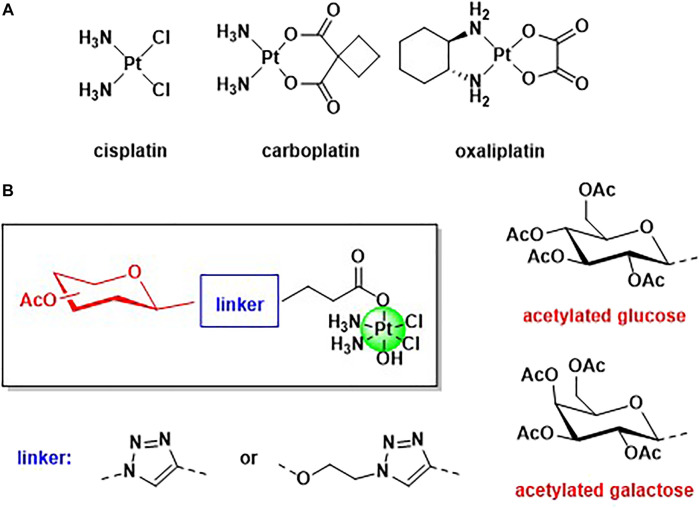
**(A)** Structure of the three worldwide approved Pt(II) based anticancer drugs, cisplatin, carboplatin, and oxalilplatin. **(B)** General structure of the novel Pt(IV) complexes based on cisplatin scaffold and functionalised with acetylated glucose and galactose.

Despite the large success of cisplatin and of the second generation Pt(II) anticancer drugs (oxalilplatin and carboplatin) for the treatment of tumors, several drawbacks and side effects are limiting their use (Figure 1A) (Johnstone et al., 2013). The main concern is the lack of selectivity of Pt(II) based drugs, and in the last 2 decades a large interest has grown in the development of selective targeted metal-based anticancer drugs (Jung and Lippard, 2007).

A very promising strategy that has been successfully adopted is the use of carbohydrates, such as glucose and galactose, as targeting vectors, exploiting the Warburg effect (Koppenol et al., 2011; Wu et al., 2016). Tumoral tissues require a higher demand of nutrients, such as sugars, to maintain the fast proliferation rate, and many cancer cells are overexpressing glucose transporters (Jang et al., 2013). Several metal-based complexes have been modified with carbohydrates with the aim to increase selectivity *versus* cancer cells, including platinum, palladium, gold, ruthenium, copper, cobalt, and tin (Pettenuzzo et al., 2016). Many glyco-modified Pt(II) complexes are reported (Kenny and Marmion, 2019) but the literature on Pt(IV) functionalized with sugars mainly refers to the interesting works reported by Wang et al. (Ma et al., 2016; 2017a; 2017b, 2018). Pt(IV) complexes show several advantages with respect to Pt(II) counterparts, being more stable and less prone to substitution reactions (Harper et al., 2010). Pt(IV) species are also called pro-drugs because they must be activated by intracellular reduction with the release of the Pt(II) scaffold and the axial ligands (Harper et al., 2010), and dual-action Pt(IV) pro-drugs are obtained when the axial positions are occupied by another relevant biological molecule (another drug, an enzyme inhibitor, a vector, etc) (Neumann et al., 2014; Ma et al., 2015; Wexselblatt et al., 2015; Gibson, 2016; Lee et al., 2018; Lo Re et al., 2018; Montagner et al., 2018; Petruzzella et al., 2018; Savino et al., 2018; Almotairy et al., 2020; Gabano et al., 2021). In this work, we present a facile synthesis of mono glyco-functionalised Pt(IV) complexes based on cisplatin scaffold and their biological applications against osteosarcoma cell lines. Osteosarcoma (OS) is the main primary bone malignant entity affecting adolescents and young adults, and it is an aggressive tumor with a tendency to metastasize and invade para-carcinoma tissues (Heymann et al., 2019). The primary treatment for this tumor is a combination of surgery and chemotherapy but unfortunately, the prognosis remains poor due to chemoresistance and early metastasis (Han et al., 2019). The absence of specific and targeted strategies for the treatment of OS increased the scientific interest in designing and synthesizing new drugs able to selective target OS cancer cells. Cisplatin is a standard drug for osteosarcoma therapy but the effectiveness of the therapy is often limited by the chemoresistance and the absence of drug specificity (Li et al., 2018). As most of the tumoral issues, also OS cancer cells exhibit increased glycolytic activity and accumulate greater quantities of sugars, compared to normal tissues (Cifuentes et al., 2011; Cura and Carruthers, 2012; Ogawa et al., 2021). It has been shown that the overexpression of sugar receptors in OS is predominantly associated with the likelihood of metastasis and poor patient prognosis (Medina and Owen, 2002).

For the first time, the carbohydrates and the platinum scaffold are linked, exploiting the Copper Azide Alkyne Cycloaddition (CUAAC) reaction, which has become the flagship of click chemistry. Click chemistry is becoming a very promising and emerging tool in synthetic medicinal chemistry because its versatility and mild conditions allow the conjugation of a plethora of functional groups (Pathak et al., 2014; Wirth et al., 2015; Lauria et al., 2020). Triazole linkers are very attractive bioisosteres that can often replace the amide bonds with increased metabolic stability (Valverde et al., 2013; Bonandi et al., 2017; Farrer and Griffith, 2020). Modification of the axial positions of a Pt(IV) scaffold with targeting vectors can be achieved using several synthetic strategies (reaction between the free carboxylic acid and oxoplatin in the presence of a coupling reagent, or reaction of an activated acyl chloride with oxoplatin) but often the final Pt(IV) compound requires purification (Zhang et al., 2013). In this paper, we will show how CUAAC chemistry can be used as a tool to link targeting carbohydrates to a Pt scaffold. We report the syntheses and characterization of four novel Pt(IV) complexes based on cisplatin and functionalized with acetylated glucose and galactose. Acetylated carbohydrate derivatives have been often found to have higher anti-cancer activity than their deprotected counterparts, likely due to an increase in lipophilicity that facilitates diffusion through cell membranes (Morris et al., 2011; Upadhyaya et al., 2016; Wang et al., 2018). In addition, we investigated the effect of the linker connecting these structural moieties by the formation of anomeric *N*-triazolyl or *O*-ethylene glycosides (Figure 1B). Anomeric triazoles can offer additional sites of interaction for glucose transporters and hence enhance the activity of these types of derivatives compared to conventional glycosides (Brito et al., 2020; Ottoni et al., 2020). The anticancer activity and the cellular uptake of the complexes have been tested against three different osteosarcoma cell lines and in a model of enriched-cancer stem cells, in order to compare *in vitro* the effectiveness with standard cisplatin.

## Experimental

### Materials and Methods

All reagents and reactants (**5** and **6**) were purchased from commercial sources. The two sources used were Sigma-Aldrich and Fluorochem. All solvents were used without further purification. Cisplatin and oxoplatin were synthesized as previously reported (Dhara, 1970; Brandon and Dabrowiak, 1984).

The elemental analysis studies (carbon, hydrogen, and nitrogen) were performed by means of a PerkinElmer 2400 series II analyzer. ESI Mass Spectra were recorded with a Waters LCT Premier XE Spectrometer. NMR: ^1^H, ^13^C, and ^195^Pt NMR spectra were obtained in a solution of CDCl_3_ or DMSO-*d*_*6*_ at 300 K, in 5-mm sample tubes, with a Bruker Advance 500 MHz spectrometer (operating at 500.13, 125.75, and 107.49 MHz, respectively). The ^1^H and ^13^C chemical shift was referenced to the residual impurity of the solvent. The external reference was Na_2_PtCl_4_ in D_2_O (adjusted to *δ* = −1628 ppm from Na_2_PtCl_6_) for ^195^Pt. The stability was followed using high-performance liquid chromatography (HPLC) with a Phenomenex Luna C18 (5 μM, 100 Å, 250 mm × 4.60 mm i.d.) column at room temperature at a flow rate of 1.0 ml/min with 254 nm UV detection. Mobile phase containing 80:20 acetonitrile (0.1% trifluoroacetic acid): water (0.1% trifluoroacetic acid): the complexes were dissolved in DMSO (0.5 ml) and diluted to a final concentration of 0.5 mM using acetonitrile and water solution (1/1) and 2 mM 4-(2-hydroxyethyl)piperazine-1-ethanesulfonic acid (HEPES) buffer (pH 6.8). Infrared (IR) spectra were recorded in the region 4,000–400 cm^−1^ on a Perkin Elmer precisely spectrum 100 FT/IR spectrometer. The solid samples were run using ATR. Compounds **7, 8** (Tropper et al., 1992), **9** (Mangunuru et al., 2015)**,** and **13–16** (Reddy et al., 2017) were prepared according to reported procedures. An extensive biological evaluation of the activity of all the compounds was performed in human osteosarcoma cell line *in vitro* models, as reported below.

### Synthesis

#### Synthesis of N-(2,3,4,6-Tetra-O-Acetyl-β-D-Glucopyranosyl-1,2,3-Triazol-4-yl)-Propanoic Acid **(9)**

Compound **7** (0.98 g, 3.412 mmol) and 4-pentynoic acid (0.2 g, 2.038 mmol) were dissolved in a mixture of tetrahydrofuran (6 ml), tert-Butanol (6 ml), and deionized water (4 ml). Separately, copper(II) sulphate pentahydrate (0.1 g, 0.08 mmol) and sodium ascorbate (0.161 g, 0.325 mmol) were dissolved in deionized water (2 ml), added to a reaction flask, and allowed to stir at r.t. overnight (16 h). The solvent was removed *in vacuo* and the residue was dissolved in DCM (15 ml) and washed with brine (2 ml × 20 ml). The organic phase was dried with MgSO_4_, filtered and the solvent was evaporated. The crude product was purified by column chromatography (1:1, petroleum ether: ethyl acetate) to yield a white solid (0.615 g, 1.304 mmol, 64%). R_*f*_ = 0.92 (90:10 DCM: MeOH) [ $\alpha {]}_{D}^{23.3}$ −17.14 (c 0.7, MeOH). ^1^H NMR (500 MHz, CDCl_3_) *δ* 7.62 (s, 1H, triaz-H), 5.85 (d, J = 9.0 Hz, 1H, H-1), 5.46–5.37 (m, 2H, H-2, H-3), 5.24 (t, J = 9.95 Hz, 1H, H-4), 4.29 (dd, J = 12.6, 5.0 Hz, 1H, H-6), 4.14 (dd, J = 12.6, 2.1 Hz, 1H, H-6′), 3.99 (ddd, J = 10.1, 5.0, 2.1 Hz, 1H, H-5), 3.05 (t, J = 7.3 Hz, 2H, triaz-C*H*_2_), 2.78 (t, J = 7.3 Hz, 2H, C*H*_2_CO), 2.08 (s, 3H, CH_3_ of OAc), 2.06 (s, 3H, CH_3_ of OAc), 2.02 (s, 3H, CH_3_ of OAc), 1.85 (s, 3H, CH_3_ of OAc) ppm. ^13^C NMR (125 MHz, CDCl_3_) *δ* 176.93 (*C*OOH), 170.70 (*C*O of OAc), 170.08 (*C*O of OAc), 169.54 (*C*O of OAc), 169.13 (*C*O of OAc), 147.04 (triaz-*C*), 119.81 (triaz-*C*H), 85.80 (C-1), 75.22 (C-5), 72.81 (C-3), 70.30 (C-2), 67.88 (C-4), 61.70 (C-6), 33.24 (*C*H_2_COOH), 20.83 (2 × *C*H_3_ of OAc), 20.68 (2 × *C*H_3_ of OAc), 20.24 (triaz-*C*H_2_) ppm. IR (ATR) 2966.47, 1738.08, 1701.08, 1429.63, 1369.28, 1218.46, 1096.44, 1037.86, 918.27, 838.17 cm^−1^. HR-MS (+): m/z calcd for C_19_H_25_N_3_O_11_ + Na^+^ (M + Na)^+^ 494.1489, found 494.1383. HR-MS (+): m/z calcd for C_19_H_25_N_3_O_11_ + H^+^ (M + H)^+^ 472.1489, found 472.1564.

#### Synthesis of N-(2,3,4,6-Tetra-O-Acetyl-β-D-Galactopyranosyl-1,2,3-Triazol-4-yl)-Propanoic Acid **(10)**

Compound **10** was prepared according to the method reported for compound **9** (Yield 0.713 g, 1.512 mmol, 74%). R_*f*_ = 0.33 (95:5 DCM: MeOH) [ $\alpha {]}_{D}^{21.6}$ −1.51 (c 0.66, MeOH). ^1^H NMR (500 MHz, CDCl_3_) *δ* 7.65 (s, 1H, triaz-H), 5.81 (d, J = 9.4 Hz, 1H, H-1), 5.57–5.50 (m, 2H, H-2, H-3), 5.23 (dd, J = 10.3, 3.4 Hz, 1H, H-4), 4.24–4.10 (m, 3H, H-5, H-6, H-6′), 3.05 (t, J = 7.4 Hz, 2H, triaz-C*H*_2_), 2.79 (t, J = 7.4 Hz, 2H, C*H*_2_CO), 2.21 (s, 3H, C*H*_3_ of OAc), 2.03 (s, 3H, C*H*_3_ of OAc), 1.99 (s, 3H, C*H*_3_ of OAc), 1.86 (s, 3H, C*H*_3_ of OAc) ppm. ^13^C NMR (125 MHz, CDCl_3_) *δ* 177.14 (*C*OOH), 170.52 (*C*O of OAc), 170.13 (*C*O of OAc), 169.97 (*C*O of OAc), 169.25 (*C*O of OAc), 146.91 (triaz-*C*), 119.88 (triaz-*C*H), 86.31 (C-1), 74.11 (C-5), 70.93 (C-4), 67.88 (C-2), 67.02 (C-3), 61.31 (C-6), 33.27 (*C*H_2_COOH), 20.81 (triaz-*C*H_2_), 20.78 (*C*H_3_ of OAc), 20.76 (*C*H_3_ of OAc), 20.61 (*C*H_3_ of OAc), 20.32 (*C*H_3_ of OAc) ppm. IR (ATR) 3087.65, 1734.80, 1715.92, 1436.51, 1366.99, 1216.01, 1045.12, 923.00, 850.50, 717.78 cm^−1^. HR-MS (+): m/z calcd for C_19_H_25_N_3_O_11_ + H^+^ (M + H)^+^ 472.1489, found 472.1564. HR-MS (+): m/z calcd for C_19_H_25_N_3_O_11_ + Na^+^ (M + Na)^+^ 494.1489, found 494.1384.

#### Synthesis of N-[2-O-(2,3,4,6-Tetra-O-Acetyl-β-D-Glucopyranosyl)-ethyl-1,2,3-Triazol-4-yl]-Propanoic Acid **(17)**

Compound **17** was prepared according to the method reported for compound **9** (Yield 0.147 g, 0.285 mmol, 44.24%). R_*f*_ = 0.35 (DCM: MeOH 95:5) [ $\alpha {]}_{D}^{21.6}$ −8.57 (c 0.7, MeOH). ^1^H NMR (500 MHz, CDCl_3_) *δ* 7.44 (s, 1H, triaz-*H*), 5.19 (t, *J* = 9.5 Hz, 1H, H-3), 5.06 (t, *J* = 9.7 Hz, 1H, H-4), 4.97 (dd, *J* = 9.6, 8.0 Hz, 1H, H-2), 4.55 (dt, *J* = 14.4, 3.8 Hz, 1H, C*H*-triaz), 4.50–4.44 (m, 1H, C*H′*-triaz), 4.43 (d, *J* = 7.9 Hz, 1H, H-1), 4.24 (dd, *J* = 12.4, 4.7 Hz, 1H, H-6), 4.19 (dt, *J* = 10.6, 4.0 Hz, 1H, OC*H*), 4.11 (dd, *J* = 12.4, 2.2 Hz, 1H, H-6′), 3.95–3.87 (m, 1H, OC*H′*), 3.68 (ddd, *J* = 10.0, 4.6, 2.3 Hz, 1H, H-5), 3.02 (t, *J* = 7.0 Hz, 2H, triaz-C*H*_*2*_), 2.77 (t, *J* = 6.7 Hz, 2H, C*H*_*2*_CO), 2.07 (s, 3H, OAc), 2.01 (s, 3H, OAc), 1.99 (s, 3H, OAc), 1.96 (s, 3H, OAc) ppm. ^13^C NMR (125 MHz, CDCl_3_) *δ* 176.42 (*C*OOH), 170.78 (*C*O of OAc), 170.48 (*C*O of OAc), 169.59 (*C*O of OAc), 169.54 (*C*O of OAc), 146.25 (*C*-triaz), 122.83 (*C*H-triaz), 100.66 (C-1), 72.67 (C-3), 72.04 (C-5), 71.17 (C-2), 68.28 (C-4), 67.89 (O*C*H_2_), 61.85 (C-6), 50.20 (CH_2_-triaz), 33.54 (*C*H_2_COOH), 20.88 (triaz-*C*H_2_), 20.84 (*C*H_3_ of OAc), 20.70 (2 × *C*H_3_ of OAc), 20.68 (*C*H_3_ of OAc) ppm. IR (ATR) 3136.33, 2954.03, 1753.42, 1742.06, 1720.47, 1428.59, 1367.14, 1251.30, 1221.16, 1167.23, 1046.93, 1031.50, 910.47, 826.13 cm^−1^. HR-MS (+): m/z calcd for C_21_H_29_N_3_O_12_ + H^+^ (M + H)^+^ 516.1751, found 516.1826. HR-MS (+): m/z calcd for C_21_H_29_N_3_O_12_ + Na^+^ (M + Na)^+^ 538.1781, found 538.1644.

#### Synthesis of N-[2-O-(2,3,4,6-Tetra-O-Acetyl-β-D-Galactopyranosyl)-ethyl-1,2,3-Triazol-4-yl]-Propanoic Acid **(18)**

Compound **18** was prepared according to the method reported for compound **9** (Yield 0.500 g, 0.969 mmol, 47%). R_*f*_ = 0.41 (DCM: MeOH 95:5) [ $\alpha {]}_{D}^{20}$ −1.45 (c 0.68, MeOH). ^1^H NMR (500 MHz, CDCl_3_) *δ* 7.46 (s, 1H, triaz-H), 5.38 (dd, *J* = 3.4, 0.9 Hz, 1H, H-4), 5.17 (dd, *J* = 10.5, 7.9 Hz, 1H, H-2), 5.01 (dd, *J* = 10.5, 3.4 Hz, 1H, H-3), 4.57 (dt, *J* = 7.7, 3.4 Hz, 1H, C*H*-triaz), 4.49 (ddd, *J* = 14.5, 8.8, 3.4 Hz, 1H, C*H′*-triaz), 4.41 (d, *J* = 7.9 Hz, 1H, H-1), 4.21 (dt, *J* = 10.6, 3.9 Hz, 1H, OC*H*), 4.13 (qd, *J* = 11.3, 6.7 Hz, 2H, H-6, H-6′), 3.95–3.87 (m, 2H, H-5, OC*H′*), 3.04 (t, *J* = 7.1 Hz, 2H, triaz-C*H*_*2*_), 2.79 (t, *J* = 7.1 Hz, 2H, C*H*_*2*_CO), 2.16 (s, 3H, CH_3_ of OAc), 2.04 (s, *J* = 3.6 Hz, 3H, CH_3_ of OAc), 1.97 (d, *J* = 2.1 Hz, 6H, 2 × CH_3_ of OAc). ^13^C NMR (125 MHz, CDCl_3_) *δ* 176.26 (*C*OOH), 170.59 (*C*O of OAc), 170.32 (2 × *C*O of OAc), 169.79 (CO of OAc), 146.22 (C-triaz), 122.91 (*C*H-triaz), 101.07 (C-1), 70.98 (C-5), 70.74 (C-3), 68.78 (C-2), 67.77 (O*C*H_2_), 67.04 (C-4), 61.32 (C-6), 50.22 (*C*H_2_-triaz), 33.49 (*C*H_2_COOH), 20.92 (triaz-*C*H_2_), 20.82 (*C*H_3_ of OAc), 20.80 (*C*H_3_ of OAc), 20.77 (*C*H_3_ of OAc), 20.69 (*C*H_3_ of OAc). IR (ATR) 2940.25, 1739.44, 1430.24, 1367.99, 1214.32, 1043.51, 955.29, 916.32, 859.50, 827.84, 734.98 cm^−1^. HR-MS (+): m/z calcd for C_21_H_29_N_3_O_12_ + H^+^ (M + H)^+^ 516.1751, found 516.1826. HR-MS (+): m/z calcd for C_21_H_29_N_3_O_12_ + Na^+^ (M + Na)^+^ 538.1781, found 538.1664.

#### Synthesis of N-(2,3,4,6-Tetra-O-Acetyl-β-D-Glucopyranosyl-1,2,3-Triazol-4-yl)-{3-Oxopropyl-[oxy(2,5-Dioxopyrrolidin-1-yl)]} **(11)**

Compound **9** (0.2 g, 0.424 mmol) and N-hydroxysuccinimide (0.058 g, 0.604 mmol) were dissolved in anhydrous DCM (7 ml) and purged with N_2_. A solution of EDCI (0.097 g, 0.607 mmol) in anhydrous DCM (2 ml) was added *via* cannula over an ice bath and the solution was stirred for 45 min. The reaction was warmed to r.t. and stirred for a further 16 h. The organic layer was washed with 0.1 M HCl (2 × 10 ml) and dried with MgSO_4_, filtered, and concentrated *in vacuo*. The product was obtained as a white solid which was reacted on without further purification (0.185 g, 0.325 mmol, 77%). R_*f*_ = 0.72 (DCM: MeOH 95:5) [ $\alpha {]}_{D}^{21.6}$ −9.85 (c 0.71, CHCl_3_). ^1^H NMR (500 MHz, CDCl_3_) *δ* 7.74 (s, 1H, C*H*-triaz), 5.83 (d, J = 9.2 Hz, 1H, H-1), 5.40–5.35 (m, 2H, H-2 and H-3), 5.24–5.18 (m, 1H, H-4), 4.25 (dd, J = 12.6, 5.1 Hz, 1H, H-6), 4.11 (dd, J = 12.6, 2.1 Hz, 1H, H-6’), 4.01–3.94 (m, 1H, H-5), 3.14 (td, J = 7.1, 3.2 Hz, 2H, triaz-C*H*_*2*_), 3.00–2.95 (m, 2H, C*H*_*2*_CO), 2.81 (s, 4H, CH_2_CH_2_-succ), 2.03 (s, 3H, OAc), 2.02 (s, 3H, OAc), 1.98 (s, 3H, OAc), 1.81 (s, 3H, OAc) ppm. ^13^C NMR (125 MHz, CDCl_3_) *δ* 170.68 (*C*O of OAc), 170.07 (*C*O of OAc), 169.52 (*C*O of OAc), 169.24 (*C*O succ x2), 169.05 (*C*O of OAc), 167.77 (*C*O), 145.83 (*C*-triaz), 120.27 (*C*H-triaz), 85.79 (C-1), 75.20 (C-5), 72.91 (C-3), 70.35 (C-2), 67.87 (C-4), 61.76 (C-6), 30.99 (*C*H_2_CO), 25.73 (*C*H_2_ *C*H_2_-succ), 21.11 (*C*H_2_-triaz), 20.83 (*C*H_3_ of OAc), 20.69 (*C*H_3_ of OAc), 20.66 (*C*H_3_ of OAc), 20.27 (*C*H_3_ of OAc) ppm. IR (ATR) 2945.90, 1732.16, 1430.40, 1367.42, 1203.35, 1064.13, 1035.83, 924.03, 813.55, 736.68 cm^−1^. HR-MS (+): m/z calcd for C_23_H_28_N_4_O_13_ + H^+^ (M + H)^+^ 569.1653, found 569.1727. HR-MS (+): m/z calcd for C_23_H_28_N_4_O_13_ + Na^+^ (M + Na)^+^ 591.1653, found 591.1554.

#### Synthesis of N-(2,3,4,6-Tetra-O-Acetyl-β-D-Galactopyranosyl-1,2,3-Triazol-4-yl)-{3-Oxopropyl-[oxy(2,5-Dioxopyrrolidin-1-yl)]} **(12)**

Compound **12** was prepared according to the method reported for compound **11** (Yield 0.131 g, 0.230 mmol, 54%). R_*f*_ = 0.15 (pet. ether: EtOAc 1:1) [ $\alpha {]}_{D}^{21.6}$ +1.42 (c 0.7, DCM). ^1^H NMR (500 MHz, CDCl_3_) *δ* 7.79 (s, 1H, *C*H-triaz), 5.81 (d, J = 9.3 Hz, 1H, H-1), 5.55 (dd, J = 12.5, 7.0 Hz, 2H, H-2 and H-4), 5.22 (dd, J = 10.3, 3.3 Hz, 1H, H-3), 4.23–4.12 (m, 3H, H-5, H-6 and H-6’), 3.18 (t, J = 7.1 Hz, 2H, triaz-C*H*_2_), 3.04 (t, J = 7.1 Hz, 2H, C*H*_2_CO), 2.84 (s, 4H, CH_2_CH_2_-succ), 2.23 (s, 3H, OAc), 2.05 (s, 3H, OAc), 2.01 (s, 3H, OAc), 1.88 (s, 3H, OAc) ppm. ^13^C NMR (125 MHz, CDCl_3_) *δ* 170.52 (*C*O of OAc), 170.18 (*C*O of OAc), 170.00 (*C*O of OAc), 169.17 (*C*O of OAc), 169.14 (*C*O Succ x2), 167.84 (*C*O), 145.84 (*C*-triaz), 120.27 (*C*H-triaz), 86.35 (C-1), 74.08 (C-5), 71.07 (C-3), 67.95 (C-2), 67.01 (C-4), 61.38 (C-6), 30.87 (triaz-*C*H_2_), 25.73 (*C*H_2_ *C*H_2_-succ), 21.01 (*C*H_2_CO), 20.81 (*C*H_3_ of OAc), 20.78 (*C*H_3_ of OAc), 20.66 (*C*H_3_ of OAc), 20.38 (*C*H_3_ of OAc) ppm. IR (ATR) 2944.04, 1731.92, 1430.13, 1368.12, 1205.23, 1045.44, 923.03, 812.54, 744.66 cm^−1^. HR-MS (+): m/z calcd for C_23_H_28_N_4_O_13_ + H^+^ (M + H)^+^ 569.1653, found 569.1726. HR-MS (+): m/z calcd for C_23_H_28_N_4_O_13_ + Na^+^ (M + Na)^+^ 591.1653, found 591.1547.

#### Synthesis of N-[2-O-(2,3,4,6-Tetra-O-Acetyl-β-D-Glucopyranosyl)-ethyl-1,2,3-Triazol-4-yl]-{3-Oxopropyl-[oxy(2,5-Dioxopyrrolidin-1-yl)]} **(19)**

Compound **19** was prepared according to the method discussed for compound **11** (0.126 g, 0.205 mmol, 87.5%). R_*f*_ = 0.75 (DCM:MeOH 95:5) [ $\alpha {]}_{D}^{21.6}$ +1.42 (c 0.7, DCM). ^1^H NMR (500 MHz, CDCl_3_) *δ* 7.49 (s, 1H, triaz-*H*), 5.11 (t, J = 9.5 Hz, 1H, H-3), 5.02 (t, J = 9.7 Hz, 1H, H-4), 4.92 (dd, J = 9.6, 7.9 Hz, 1H, H-2), 4.51 (ddd, J = 14.5, 4.6, 3.7 Hz, 1H, C*H*-triaz), 4.48–4.43 (m, 2H, H-1 and C*H′*-triaz), 4.21–4.11 (m, 2H, H-6′ and OC*H*), 4.06 (dd, J = 12.3, 2.3 Hz, 1H, H-6), 3.88 (ddd, J = 10.7, 8.4, 3.6 Hz, 1H, OC*H*), 3.66 (ddd, J = 10.0, 4.8, 2.4 Hz, 1H, H-5), 3.09 (dd, J = 10.7, 4.1 Hz, 2H, triaz-C*H*_*2*_), 3.00–2.95 (m, 2H, C*H*_*2*_CO), 2.80 (d, J = 5.7 Hz, 4H, C*H*_*2*_C*H*_*2*_-succ), 2.02 (s, 3H, OAc), 1.96 (s, 3H, OAc), 1.93 (s, 3H, OAc), 1.90 (s, 3H, OAc) ppm. ^13^C NMR (125 MHz, CDCl_3_) *δ* 170.76 (*C*O of OAc), 170.28 (*C*O of OAc), 169.54 (*C*O of OAc), 169.42 (*C*O of OAc), 169.22 (*C*O succ x2), 168.06 (*C*O), 144.96 (*C*-triaz), 123.04 (*C*H-triaz), 100.70 (C-1), 72.69 (C-3), 72.06 (C-5), 71.11 (C-2), 68.37 (C-4), 67.98 (O*C*H_2_), 61.92 (C-6), 50.08 (*C*H_2_-triaz), 30.95 (*C*H_2_CO), 25.72 (*C*H_2_ *C*H_2_-succ), 21.00 (triaz-*C*H_2_), 20.87 (*C*H_3_ of OAc), 20.71 (3 × *C*H_3_ of OAc) ppm. IR (ATR) 2955.80, 1733.40, 1430.16, 1366.33, 1208.17, 1033.60, 907.61, 812.61, 733.58, 700.28 cm^−1^. HR-MS (ESI+): m/z calcd for C_25_H_32_N_4_O_14_ + H^+^ (M + H)^+^ 613.1915, found 613.1985. HR-MS (ESI+): m/z calcd for C_25_H_32_N_4_O_14_ + Na^+^ (M + Na)^+^ 635.1915, found 635.1806.

#### Synthesis of N-[2-O-(2,3,4,6-Tetra-O-Acetyl-β-D-Galactopyranosyl)-ethyl-1,2,3-Triazol-4-yl]-{3-Oxopropyl-[oxy(2,5-Dioxopyrrolidin-1-yl)]} **(20)**

Compound **20** was prepared according to the method reported for compound **11** (Yield 0.106 g, 0.173 mmol, 79%). R_*f*_ = 0.69 (DCM: MeOH 95:5) [ $\alpha {]}_{D}^{21.6}$ −1.49 (c 0.67, DCM). ^1^H NMR (500 MHz, CDCl_3_) *δ* 7.48 (s, 1H, triaz-*H*), 5.33 (d, *J* = 2.7 Hz, 1H, H-4), 5.12 (dd, *J* = 10.5, 7.9 Hz, 1H, H-2), 4.93 (dd, *J* = 10.5, 3.4 Hz, 1H, H-3), 4.53 (dt, *J* = 14.4, 3.9 Hz, 1H, C*H*-triaz), 4.49–4.42 (m, 1H, C*H′*-triaz), 4.40 (d, *J* = 7.9 Hz, 1H, H-1), 4.19 (dt, *J* = 10.4, 4.1 Hz, 1H, OC*H′*), 4.08 (ddd, *J* = 25.0, 11.3, 6.6 Hz, 2H, H-6, H-6′), 3.93–3.85 (m, 2H, H-5, OC*H*), 3.11 (t, *J* = 6.8 Hz, 2H, triaz-C*H*_*2*_), 3.03–2.97 (m, 2H, C*H*_*2*_CO), 2.80 (s, *J* = 12.2 Hz, 4H, C*H*_*2*_C*H*_*2*_-succ), 2.11 (s, 3H, C*H*_*3*_ of OAc), 2.00 (s, 3H, C*H*_*3*_ of OAc), 1.92 (s, 3H, C*H*_*3*_ of OAc), 1.91 (s, 3H, C*H*_*3*_ of OAc). ^13^C NMR (125 MHz, CDCl_3_) *δ* 170.53 (*C*O of OAc), 170.33 (*C*O of OAc), 170.21 (*C*O of OAc), 169.57 (*C*O of OAc), 169.19 (*C*O succ x2), 168.10 (*C*O), 144.95 (*C*-triaz), 123.03 (*C*H-triaz), 101.11 (C-1), 70.98 (C-5), 70.77 (C-3), 68.65 (C-2), 67.79 (O*C*H_2_), 67.03 (C-4), 61.32 (C-6), 50.07 (*C*H_2_-triaz), 30.89 (*C*H_2_CO), 25.73 (*C*H_2_H_2_-succ), 20.99 (triaz-CH_2_), 20.82 (2 × *C*H_3_ of OAc), 20.81 (*C*H_3_ of OAc), 20.69 (*C*H_3_ of OAc). IR (ATR) 2959.63, 1732.72, 1429.93, 1367.59, 1211.91, 1045.63, 915.63, 812.19, 731.78 cm^−1^. HR-MS (+): m/z calcd for C_25_H_32_N_4_O_14_ + H^+^ (M + H)^+^ 613.1915, found 613.1983. HR-MS (+): m/z calcd for C_25_H_32_N_4_O_14_ + H^+^ (M + H)^+^ 635.1915, found 635.1804.

#### Synthesis of Complex **1**

Compound **11** (0.140 g, 0.246 mmol) was added to a suspension of oxoplatin (0.086 g, 0.270 mmol) in DMSO (5 ml) and stirred at 60°C for 16 h. Residual oxoplatin was filtered through cotton wool and the solvent was removed by lyophilization. The oily residue was dissolved in acetone and the product was precipitated with diethyl ether and collected by centrifugation. The yellow-white solid was washed with diethyl ether and dried *in vacuo* (0.083 g, 0.105 mmol, 43%) [ $\alpha {]}_{D}^{21.6}$ −10.34 (c 0.58, DCM). ^1^H NMR (500 MHz, DMSO) *δ* 8.16 (s, 1H, C*H*-triaz), 6.25 (d, *J* = 8.8 Hz, 1H, H-1), 5.96 (br. t, 6H, 2 × NH_3_), 5.60–5.49 (m, 2H, H-2,H-3), 5.12 (t, *J* = 9.6 Hz, 1H, H-4), 4.34 (ddd, *J* = 10.0, 5.2, 2.2 Hz, 1H, H-5), 4.13 (dd, *J* = 12.5, 5.4 Hz, 1H, H-6), 4.08–4.03 (m, 1H, H-6’), 2.80 (t, *J* = 7.6 Hz, 2H, triaz-C*H*_*2*_), 2.46 (t, *J* = 7.7 Hz, 2H, CH_2_CO), 2.01 (s, 3H, OAc), 1.99 (s, 3H, OAc), 1.95 (s, 3H, OAc), 1.78 (s, 3H, OAc) ppm. ^13^C NMR (125 MHz, DMSO) *δ* 179.90 (*C*OOPt), 170.34 (*C*O of OAc), 169.82 (*C*O of OAc), 169.61 (*C*O of OAc), 168.80 (*C*O of OAc), 147.32 (*C*-triaz), 121.44 (*C*H-triaz), 83.89 (C-1), 73.40 (C-5), 72.29 (C-3), 70.30 (C-2), 67.74 (H-4), 61.91 (C-6), 35.93 (*C*H_2_CO), 21.95 (triaz-*C*H_2_), 20.73 (*C*H_3_ of OAc), 20.54 (*C*H_3_ of OAc), 20.42 (*C*H_3_ of OAc), 20.09 (*C*H_3_ of OAc) ppm. ^195^Pt(^1^H) NMR (108 MHz, DMSO) *δ* 1041.75 ppm. IR (ATR) 3212.46, 1746.40, 1627.32, 1367.46, 1215.23, 1035.51, 924.48, 822.96 cm^−1^. HR-MS (-): m/z calcd for C_19_H_31_Cl_2_N_5_O_12_Pt–H^−^ (M-H)^−^ 786.4640, found 786.0898. El. Anal. Calcd. for C_19_H_31_Cl_2_N_5_O_12_Pt: % C = 28.29; H = 3.97; N = 8.89; found: % C = 28.88; H = 4.18; N = 8.52.

#### Synthesis of Complex **2**

Complex **2** was prepared according to the method reported for complex **1** (Yield 0.065 g, 0.082 mmol, 35.9%) [ $\alpha {]}_{D}^{21.6}$ −2.85 (c 0.7, DCM). ^1^H NMR (500 MHz, DMSO) *δ* 8.12 (s, 1H, triaz-C*H*), 6.18 (d, *J* = 9.2 Hz, 1H, H-1), 5.95 (br. t., 6H, 2 × NH_3_), 5.58 (t, *J* = 10.1 Hz, 1H, H-2), 5.45 (dd, *J* = 10.1, 3.5 Hz, 1H, H-3), 5.40 (dd, *J* = 3.4, 1.0 Hz, 1H, H-4), 4.59–4.56 (t, *J* = 6.75 Hz, 1H, H-5), 4.13 (dd, *J* = 11.6, 5.1 Hz, 1H, H-6), 4.01 (dd, *J* = 11.5, 7.3 Hz, 1H, H-6’), 2.82 (t, *J* = 7.25 Hz, 2H, triaz-C*H*_*2*_), 2.47–2.46 (m, 2H, C*H*_*2*_CO), 2.19 (s, 3H, CH_3_ of OAc), 1.99 (s, 3H, CH_3_ of OAc), 1.94 (s, 3H, CH_3_ of OAc), 1.82 (s, 3H, CH_3_ of OAc) ppm. ^13^C NMR (125 MHz, DMSO) *δ* 179.66 (*C*OOPt), 170.03 (*C*O of OAc), 169.94 (*C*O of OAc), 169.48 (*C*O of OAc), 168.56 (*C*O of OAc), 147.12 (*C*-triaz), 121.44 (*C*H-triaz), 84.19 (C-1), 72.80 (C-5), 70.43 (C-3), 67.78 (C-2), 67.29 (C-4), 61.58 (C-6), 35.92 (*C*H_2_CO), 21.79 (triaz-*C*H_2_), 20.51 (*C*H_3_ of OAc), 20.47 (*C*H_3_ of OAc), 20.34 (*C*H_3_ of OAc), 20.02 (*C*H_3_ of OAc) ppm. ^195^Pt{^1^H} NMR (108 MHz, DMSO) *δ* 1044.64 ppm. IR (ATR) 3207.92, 1746.25, 1625.63, 1368.09, 1214.56, 1050.93, 922.78 cm^−1^. HR-MS (+): m/z calcd for C_19_H_31_Cl_2_N_5_O_12_Pt + H^+^ (M + H)^+^ 786.4640, found 786.1041. El. Anal. Calcd. for C_19_H_31_Cl_2_N_5_O_12_Pt: % C = 28.98; H = 3.97; N = 8.89; found: % C = 28.48; H = 4.18; N = 8.52. 786.0994.

#### Synthesis of Complex **3**

Complex **3** was prepared according to the method reported for complex **1** (0.05 g, 0.060 mmol, 29%) [ $\alpha {]}_{D}^{21.6}$ −1.66 (c 0.6, DCM). ^1^H NMR (500 MHz, CDCl_3_) *δ* 7.76 (s, 1H, triaz-C*H*), 5.87 (br t, 6H, 2 × NH_3_), 5.23 (t, *J* = 9.6 Hz, 1H, H-3), 4.89 (t, *J* = 9.7 Hz, 1H, H-4), 4.81 (d, *J* = 8.0 Hz, 1H, H-1), 4.75–4.69 (m, 1H, H-2), 4.53–4.40 (m, 2H, C*H*_*2*_-triaz), 4.18 (dd, J = 12.3, 5.0 Hz, 1H, H-6), 4.08 (dt, *J* = 8.9, 4.1 Hz, 1H, OC*H*), 4.02 (dd, *J* = 12.2, 2.1 Hz, 1H, H-6′), 3.98 (ddd, *J* = 9.9, 4.9, 2.4 Hz, 1H, H-5), 3.89 (ddd, *J* = 11.4, 7.8, 4.0 Hz, 1H, OC*H’*), 2.81–2.77 (m, 2H, triaz-C*H*_*2*_), 2.48–2.43 (m, 2H, C*H*_*2*_CO), 2.02 (s, 3H, CH_3_ of OAc), 1.97 (s, 3H, CH_3_ of OAc), 1.92 (s, 3H, CH_3_ of OAc), 1.88 (s, 3H, CH_3_ of OAc) ppm. ^13^C NMR (125 MHz, CDCl_3_) *δ* 179.88 (*C*OOPt), 170.08 (*C*O of OAc), 169.53 (*C*O of OAc), 169.28 (*C*O of OAc), 168.95 (*C*O of OAc), 146.14 (*C*-triaz), 122.47 (*C*H-triaz), 99.20 (C-1), 71.90 (C-3), 70.64 (C-2), 70.59 (C-5), 68.08 (C-4), 67.45 (O*C*H_2_), 61.64 (C-6), 49.06 (*C*H_2_-triaz), 36.16 (*C*H_2_CO), 21.97 (triaz-CH_2_), 20.54 (*C*H_3_ of OAc), 20.38 (*C*H_3_ of OAc), 20.29 (*C*H_3_ of OAc), 20.27 (*C*H_3_ of OAc) ppm. ^195^Pt{^1^H} NMR (108 MHz, DMSO) *δ* 1047.09 ppm. IR (ATR) 3214.82, 1745.13, 1626.73, 1430.32, 1365.92, 1217.35, 1034.85, 908.82, 699.33 cm^−1^. HR-MS (+): m/z calcd for C_21_H_35_Cl_2_N_5_O_13_Pt + H^+^ (M + H)^+^ 832.5170, found 832.1316. HR-MS (+): m/z calcd for C_21_H_35_Cl_2_N_5_O_13_Pt + Na^+^ (M + H)^+^ 854.5170, found 854.1134. El. Anal. Calcd. for C_21_H_35_Cl_2_N_5_O_13_Pt: % C = 30.33; H = 4.24; N = 8.42; found: % C = 30.79; H = 4.61; N = 8.90.

#### Synthesis of Complex **4**

Complex **4** was prepared according to the method reported for complex **1** (0.07 g, 0.084 mmol, 38%) [ $\alpha {]}_{D}^{21.6}$ +43.1 (c 0.58, DCM). ^1^H NMR (500 MHz, DMSO) *δ* 7.75 (s, 1H, triaz-C*H*), 5.82 (br t, 6H, 2 × NH_3_), 5.24 (dd, *J* = 3.5, 0.8 Hz, 1H, H-4), 5.12 (dd, *J* = 10.4, 3.6 Hz, 1H, H-3), 4.88 (dd, *J* = 10.4, 8.0 Hz, 1H, H-2), 4.71 (d, *J* = 8.0 Hz, 1H, H-1), 4.52–4.40 (m, 2H, C*H*_2_-triaz), 4.19 (dd, *J* = 7.2, 6.3 Hz, 1H, H-5), 4.12–4.00 (m, 3H, H-6, H-6′, OC*H*), 3.93–3.86 (m, 1H, OC*H*’), 2.82–2.78 (t, *J* = 7.25 Hz, 2H, triaz-C*H*_2_), 2.47 (dd, *J* = 8.6, 6.9 Hz, 2H, C*H*_2_CO), 2.11 (s, 3H, C*H*_3_ of OAc), 2.01 (s, 3H, C*H*_3_ of OAc), 1.90 (s, 3H, C*H*_3_ of OAc), 1.89 (s, 3H, C*H*_3_ of OAc) ppm. ^13^C NMR (125 MHz, DMSO) *δ* 179.86 (*C*OOPt), 169.93 (*C*O of OAc), 169.91 (*C*O of OAc), 169.48 (*C*O of OAc), 169.05 (*C*O of OAc), 146.13 (*C*-triaz), 122.43 (*C*H-triaz), 99.67 (C-1), 70.07 (C-5), 69.94 (C-3), 68.34 (C-2), 67.30 (C-4), 67.26 (O*C*H_2_), 61.23 (C-6), 49.09 (C*H*_2_-triaz), 36.13 (*C*H_2_CO), 21.97 (triaz-*C*H_2_), 20.52 (*C*H_3_ of OAc), 20.40 (*C*H_3_ of OAc), 20.32 (2x*C*H_3_ of OAc) ppm. ^195^Pt{^1^H} NMR (108 MHz, DMSO) *δ* 1047.15 ppm. IR (ATR) 3214.92, 1740.41, 1631.39, 1429.52, 1367.30, 1217.81, 1173.03, 1045.52, 952.53 cm^−1^. HR-MS (+): m/z calcd for C_21_H_35_Cl_2_N_5_O_13_Pt + H^+^ (M + H)^+^ 832.5170, found 854.1139. HR-MS (+): m/z calcd for C_21_H_35_Cl_2_N_5_O_13_Pt + H^+^ (M + Na)^+^ 854.5170, found 854.1315. El. Anal. Calcd. for C_21_H_35_Cl_2_N_5_O_13_Pt: % C = 30.33; H = 4.24; N = 8.42; found: % C = 29.98; H = 4.69; N = 8.01.

### *In vitro* Biological Evaluation

*In vitro* tests of cisplatin-based drugs were performed to evaluate the cellular behaviors in response to the different compounds (1–4) compared to cisplatin. All the drugs were reconstituted in Dimethyl Sulfoxide (DMSO) at 1 mg/ml final concentration, and then dissolved in the culture media at different concentrations: 15, 30, and 60 µM. Three different osteosarcoma cells lines (MG63, SAOS-2, U-2OS) and an *in vitro* model of osteosarcoma stem cells (enriched-CSCs) were maintained in culture with and without the drugs for 72 h.

#### Cell Culture

Human Osteosarcoma cell lines MG63 (ATCC^®^ CRL1427™), U-2OS (ATCC^®^ HTB-96™), and SAOS-2 (ATCC^®^ HTB-85™), purchased from American Type Culture Collection (ATCC), were used. MG63 cell line was cultured in DMEM F12-GlutaMAX™ Modified Medium (Gibco) supplemented with 10% Foetal Bovine Serum (FBS) (Gibco) and 1% of penicillin/streptomycin mixture (pen/strep) (100 U/ml—100 μg/ml, Gibco). SAOS-2 and U-2OS cell lines were cultured in McCoy’s 5A Modified Medium (Gibco) supplemented with 15 and 10% FBS, respectively, and 1% pen/strep. Cells were kept in an incubator at 37°C under controlled humidity and 5% CO_2_ atmosphere conditions. Cells were detached from culture flasks by trypsinization and centrifuged. The cell number and viability were determined by Trypan Blue Dye Exclusion test and all cell handling procedures were performed under a laminar flow hood in sterility conditions. For the experiment, all cell lines were seeded 5.0 × 10^3^ cells/well in 96 well-plates and 5.0 × 10^4^ cells/well in 6 well-plates.

#### Enriched-CSCs Culture

Enriched-cancer stem cells (CSCs) were obtained under specific culture conditions as reported in the literature (Brown et al., 2017; Bassi et al., 2020) as a sarcosphere-forming method starting from a human MG63 osteosarcoma cell line. The MG63 cell line was seeded in Ultra-Low Attachment T25 flasks (Corning Inc., NY) with a density of 2.0 × 10^3^ cells/cm^2^ in serum-free DMEM F12-GlutaMAX™ Modified Medium supplemented with a specific cocktail of factors: 10 μL/ml N2 (Gibco), 20 μL/ml B27 (Gibco), 0.1 μL/ml human Basic-Fibroblast Growth Factor (bFGF) (Invitrogen), and 0.01 μL/ml human Epidermal Growth Factor (EGF) (PeproTech). The cocktail was added to each flask every 2/3 days for a total of 10 days of culture. After their formation, the CSCs were collected and centrifugated for 10 min at 130 × g; the pellet was resuspended in the same medium conditions, well mixed, and directly seeded in Ultra-Low Attachment 96 well-plate and Ultra-Low Attachment 6 well-plate with 200 µL/well and 1.5 ml/well volume of cell culture medium, respectively. The factors’ cocktail was added every 2/3 days during the experiment following the above-reported manufacturer’s instructions.

#### MTT Cell Viability Assay

A quantitative analysis of cell viability and proliferation was carried out by MTT assay on cell cultures, by using the cells only as a negative control. At 72 h, the MTT assay was performed according to the manufacturer’s instructions. Briefly, MTT reagent [3-(4,5-dimethylthiazol-2-yl)-2,5-diphenyltetrazolium bromide] (5 mg/ml) was dissolved in Phosphate Saline Buffer 1X (PBS 1X). At 72 h, the cells were incubated with 10% media volume MTT solution for 2 h at 37°C, 5% CO_2_, and controlled humidity conditions. The cell culture media was removed and substituted with DMSO (Sigma) dissolving formazan crystals derived from MTT conversion by metabolically active cells. For CSCs, the total media was centrifugated and the deposited crystals were directly resuspended in DMSO. After 15-min of incubation under slight stirring conditions, the absorbance of formazan was read at 570 nm by using a Multiskan FC Microplate Photometer (Thermo Scientific). The values of absorbance are directly proportional to the number of metabolic active cells in each well. The experiment was carried out with three biological replicates for each condition.

#### Cell Morphology Evaluation

Cells treated with and without the drugs (30 µM) were fixed in 4% buffered Paraformaldehyde (PFA) following the manufacturer’s instructions. The fixed cells were permeabilized in PBS 1X with 0.1% (v/v) Triton X-100 (Sigma) for 5 min at room temperature and F-actin filaments were highlighted with a red fluorescent solution of Rhodamine Phalloidin (Actin Red 555 Ready Probes™ Reagent, Invitrogen), following the company indications, for 30 min at room temperature. DAPI (600 nM) counterstaining was performed for cell nuclei identification, following the manufacturer’s instructions. The images were acquired by using an Inverted Ti-E Fluorescent Microscope.

#### Inductively Coupled Plasma-Optical Emission Spectrometry

The ICP-OES (Agilent Technologies 5100 ICP-OES, Santa Clara, United States) was performed on Enriched-CSCs culture to quantify cellular internalization of drugs, following the manufacturer’s instructions. At 72 h, cells were mechanically by 50–100 times p200 pipetting to disaggregate spheroid, counted by Trypan Blue Dye Exclusion Test, and collected in 400 µL PBS 1X. ICP-OES was used for the quantitative determination of platinum ions content per cell derived by cisplatin-based drugs, by using cells only as a negative control. Briefly, the samples were dissolved in 500 µL nitric acid (65 wt%) and 2.1 ml of milliQ water followed by 30 min of sonication in an ultrasonicator bath. The analytical wavelength of Pt was 265.945 nm. One experiment was carried out and for each condition, the amount of drug per cell was quantified in biological triplicate. The data are represented in the graph.

#### Statistical Analysis

Statistical analysis was performed by using GraphPad Prism Software (8.0.1 version). The results of the MTT assays are reported in the graphs as mean percentage of cell viability with respect to cells only ± standard deviation, and they were analyzed by Two-way analysis of variance (Two-way ANOVA) and Tukey’s multiple comparisons test. IC_50_ values were calculated as log(inhibitor) versus mean percentage of dead cells with respect to cells only, and the obtained values are reported in the graphs ± 95% confidence interval (CI) for each cell line. The results of MTT assays on CSCs are reported in the graph as mean percentage of cell viability with respect to cells only ± the standard error of the mean, and they were analyzed by Unpaired *t*-test setting the *p* value ≤ 0.05 to determine statistically significant differences. The ICP-OES data were elaborated as picograms of iron ions per cell and reported in the graph ± standard error of the mean. The results were analyzed by One-way analysis of variance (One-way ANOVA) and Dunnett’s multiple comparisons test (*p value ≤ 0.05, **p value ≤ 0.01, ***p value ≤ 0.001, ****p value ≤ 0.0001).

## Results and Discussion

**FIGURE 2:**
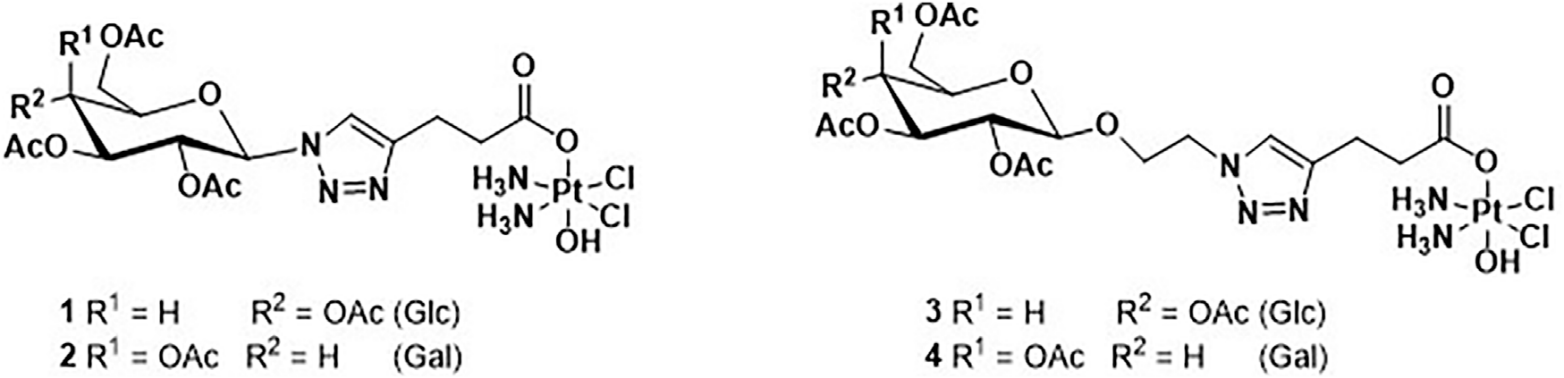
Structure of the four novel glyco-Pt(IV) complexes **(1–4)**.

The four novel glyco-Pt(IV) complexes are shown in Figure 2. Complexes **1** and **2** are β-anomeric triazolyl *N*-glycosides which have the triazole group directly attached to the anomeric carbon (**1**, glucose, and **2** galactose) while complexes **3** and **4** have an additional *O*-ethylene linker (**3**, glucose, and **4** galactose).

**SCHEME 1:**
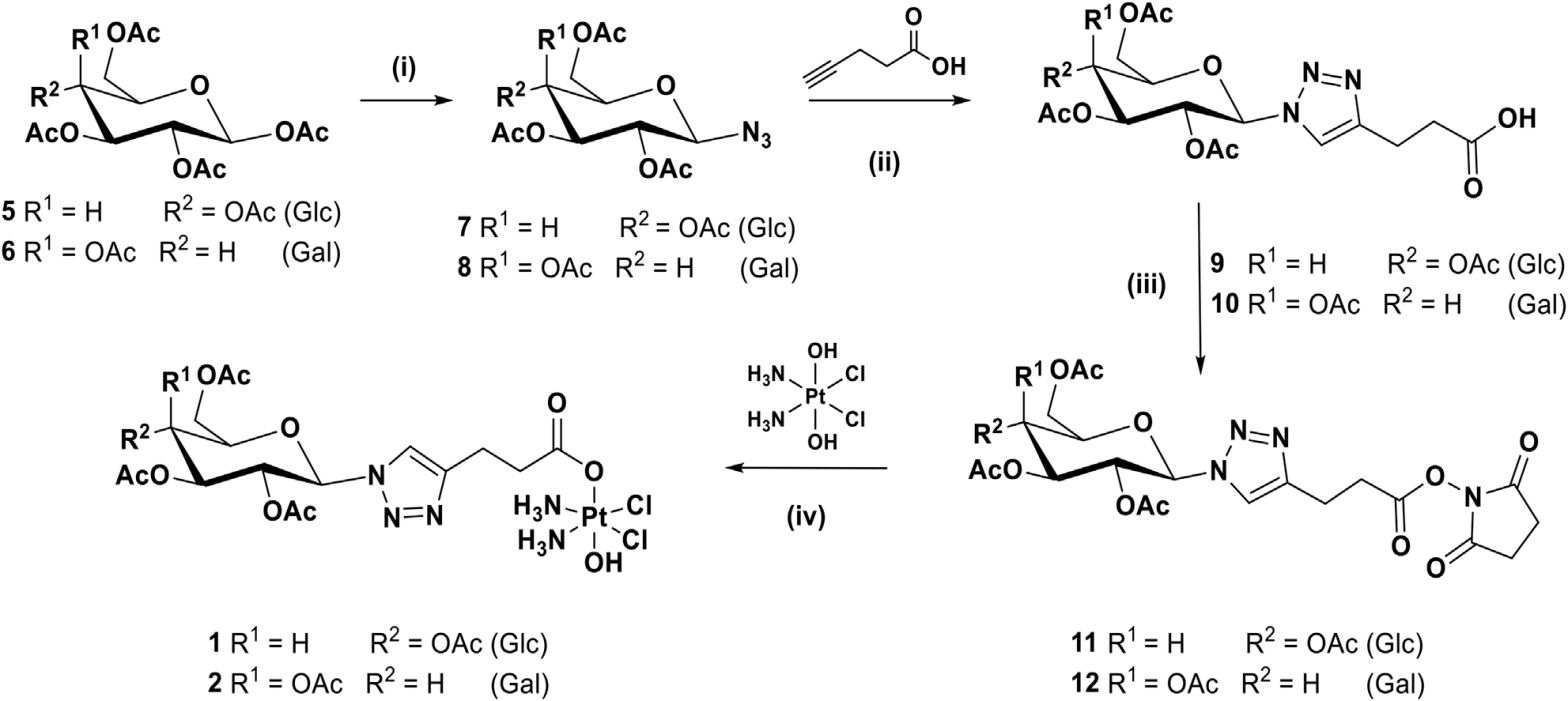
Synthetic route for the complexes **1** and **2**: (i) TMSN_3_, SnCl_4_, DCM, rt, 16 h, 91%; (ii) CuSO_4_, sodium ascorbate, *t*-BuOH, THF, H_2_O, rt, 16 h, 64%; (iii) EDCI, NHS, DCM, rt, 16 h, 77%; (iv) DMSO, 60°C, 16 h, 43%.

The synthetic route for complexes **1** and **2** can be seen in Scheme 1. Briefly, the per-acetylated glucose **5** and galactose **6** were transformed to the corresponding β-azides at the anomeric carbon, **7** and **8** respectively (Tropper et al., 1992), by reaction with azidotrimethylsilane and tin tetrachloride. Compounds **7** and **8** were then reacted with pentynoic acid, using CUAAC conditions at room temperature, to produce the carboxylic acids **9** and **10** (Mangunuru et al., 2015). These latter carboxylic acids are activated, forming NHS esters **11** and **12,** respectively, that were reacted with oxoplatin to produce the final desired compounds **1** and **2**.

**SCHEME 2:**
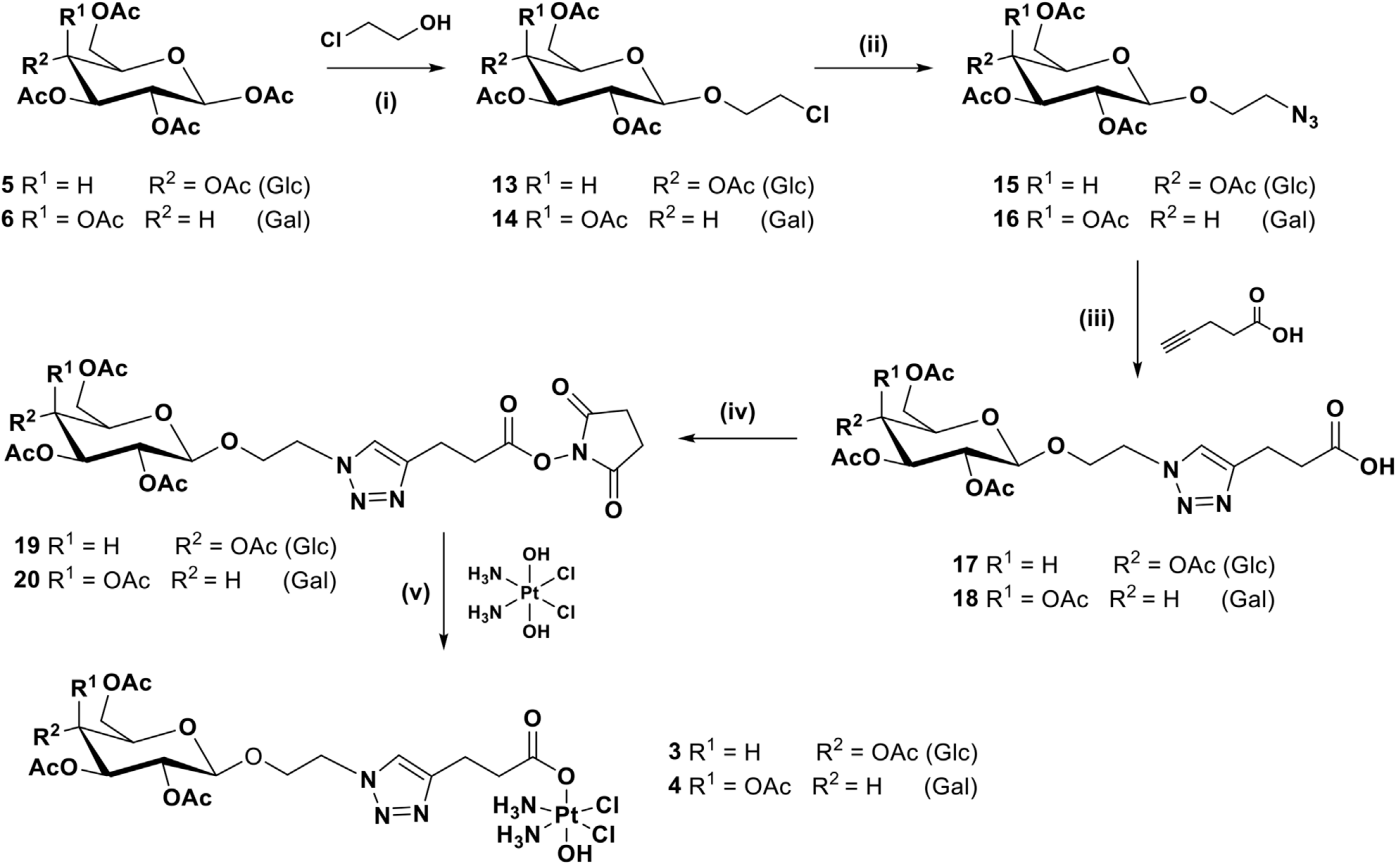
Synthetic route for complexes **3** and **4**: (i) BF_3_.OEt_2_, 3 Å MS, 0°C to rt, DCM, 16 h, 39%; (ii) NaN_3_, DMF, 80°C, 16 h, 59%; (iii) CuSO_4_, sodium ascorbate, *t*-BuOH, THF, H_2_O, rt, 16 h, 44.24%; (iv) EDCI, NHS, DCM, rt, 16 h, 87.5%; (v) DMSO, 60°C, 16 h, 29%.

For complexes **3** and **4**, with an *O*-ethylene spacer between the anomeric carbon and the triazole ring, the synthetic route requires one more step, as shown in Scheme 2, whereby the per-acetylated sugars **5** and **6** were reacted with chloroethanol in the presence of boron trifluoride diethyl etherate to produce **13** and **14** (Reddy et al., 2017). They are then transformed to the corresponding azides **15** and **16** before being reacted with pentynoic acid under similar CUAAC conditions used previously, to give acids **17** and **18**. Activation to form NHS esters **19** and **20** followed by reaction with oxoplatin produced the final desired complexes **3** and **4**.

All the intermediate compounds (**5**–**20**) have been obtained with moderate to good yields and have been characterized with multinuclear NMR, IR, and LC(HR)-MS. The final complexes containing platinum (**1–4**) have been characterized with multinuclear NMR (^1^H, ^13^C, and ^195^Pt), HR-MS, and Elemental Analyses to assess the purity. All the data and spectra can be found in the Experimental Section and the Supplementary Material.

The physiological stability has been evaluated with HPLC in DMSO/HEPES at pH 6.8 buffer and the complexes are stable (little decomposition of 8% observed after 1 week at r.t., see Supplementary Material for complex **4**).

**FIGURE 3:**
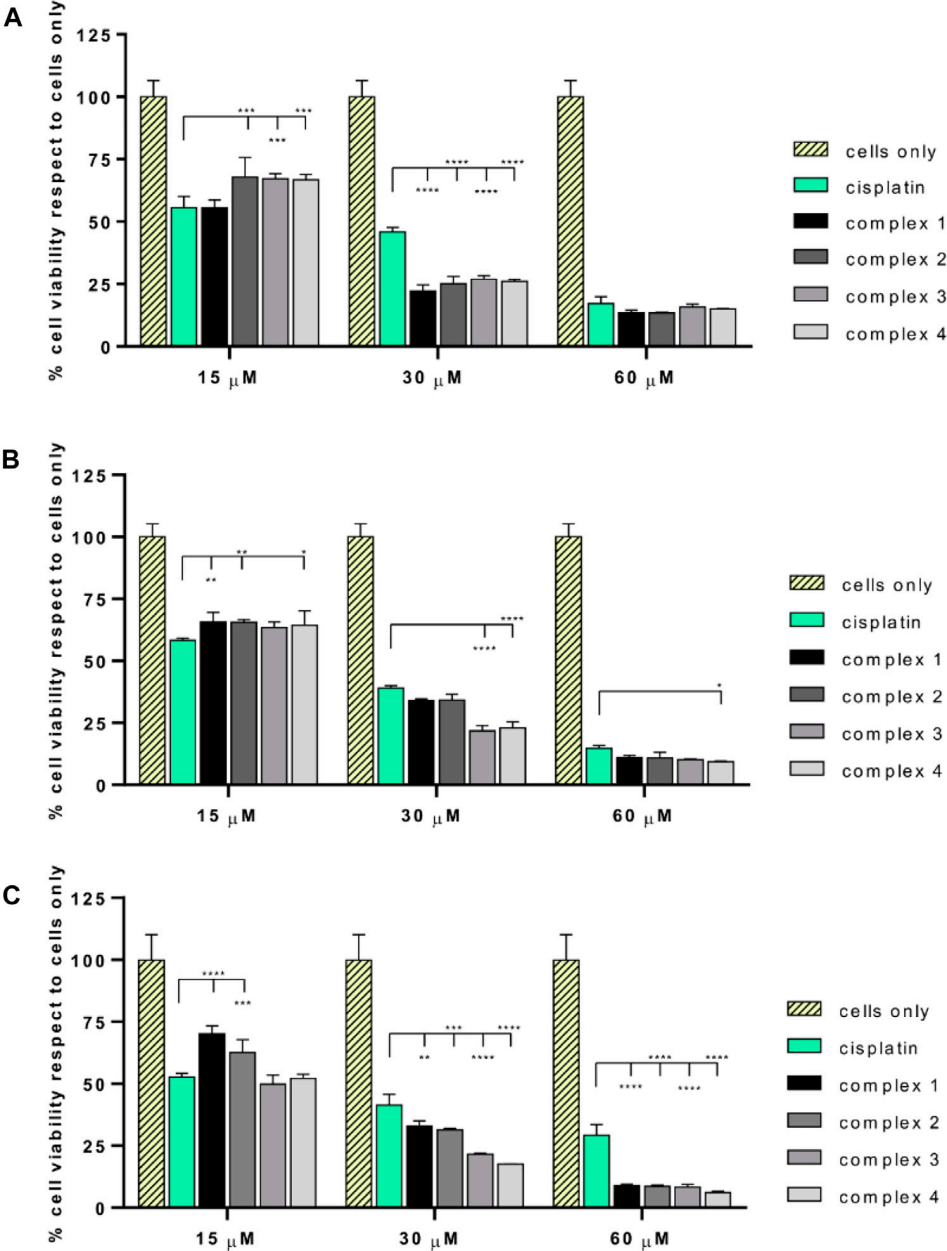
MTT assay of SAOS-2, U-2 OS, and MG63**.** Percentage of cell viability (mean ± SEM) respect to cells only is reported in the graphs for SAOS-2 **(A)**, U-2 OS **(B)** and MG63 **(C)** after 72 h of drug exposure. Statistically significant differences respect to cisplatin are reported in the graphs (*p value ≤ 0.05, **p value ≤ 0.01, ***p value ≤ 0.001, ****p value ≤ 0.0001).

The biological effect of the complexes **1–4,** in comparison with the effect of cisplatin has been evaluated on different osteosarcoma cells lines. All the drugs showed a dose-dependent anticancer effect (Figure 3) in all the tested cancer cell lines. A statistically significant decrease of cell viability induced by all the drug concentrations (*p* value ≤ 0.0001) has been reported with respect to the cells grown without drugs (cells only). Most excitingly, starting from 30 μM, the complexes showed a significantly higher cytotoxicity with respect to cisplatin. Looking in detail at the single-cell lines, it was possible to observe phenotypic-dependent behaviors in response to the different complexes. In detail, the viability of SAOS-2 (Figure 3A) was significantly decreased in the presence of the four complexes with respect to cisplatin when supplied at 30 µM (*p* value ≤ 0.0001); conversely the viability of U-2 OS (Figure 3B) was significantly reduced only in the presence of **3** and **4** compared to cisplatin at 30 µM (*p* value ≤ 0.0001), and only in the presence of complex **4** at 60 µM (*p* value ≤ 0.05). All the complexes seem to be more effective with respect to cisplatin in the MG63 cell line (Figure 3C), starting from 30 µM and at higher concentrations, the anticancer effect greatly increases (*p* value ≤ 0.0001). The IC_50_ values (μM) reported in Table 1, confirmed the phenotypic-dependent effect of the different complexes. In fact, in SAOS-2 the most effective drug is complex **1**, showing a IC_50_ of 16.48 (−1.84; +2.08), while in U-2 OS and MG63 complex **3** has the best IC_50_ values of 18.57 (−1.3; +1.4) and 14.88 (−0.91; +0.98), respectively (see Supplementary Material).

**TABLE 1:** IC_50_ (µM) values of Cisplatin and complexes **1–4** on OS cell lines.

Complex	Cisplatin		1		2		3		4	
OS cancer cell	IC_50_ (µM)	95% CI	IC_50_ (µM)	95% CI	IC_50_ (µM)	95% CI	IC_50_ (µM)	95% CI	IC_50_ (µM)	95% CI
SAOS-2	20.42	−4.13; +5.17	16.48	−1.84; +2.08	20.18	−2.38; +2.7	20.39	−2.12; +2.37	20.1	−1.99; +2.21
U-2 OS	19.85	−1.64; +1.8	16.48	−0.98; +1.02	21.09	−0.86, +0.89	18.57	−1.3; +1.4	18.91	−1.5; +1.63
MG63	17.8	−3.34; +4.13	21.9	−0.86; +0.89	19.68	−1.27; +1.36	14.88	−0.91; +0.98	15.5	−0.54; +0.55

**FIGURE 4:**
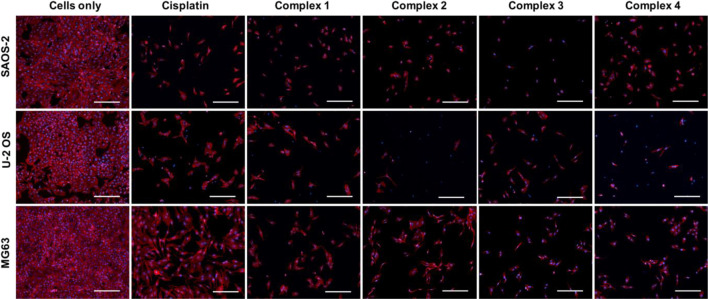
Cell morphology evaluation on SAOS-2, U-2 OS, and MG63. Actin and DAPI staining of osteosarcoma cell lines treated with and without drugs (30 µM) for 72 h. F-actin filaments in red; cell nuclei in blue. Scale bars 200 µm.

The qualitative analysis of cell morphology confirmed the cytotoxicity results (Figure 4). As shown in the panel, the number of all the cells treated with cisplatin and complexes **1–4** drastically decreased compared to cells only which, on the contrary, showed a higher cell density. The different behaviors observed among the osteosarcoma cell lines, induced by the different drugs, are ascribable to the well-known different degrees of genetic complexity of each cell line, inducing cell-specific biological behaviors (e.g., tumorigenicity, colony-forming ability, invasive/migratory potential, metabolism, and proliferation capacity) (Lauvrak et al., 2013; Liu et al., 2016).

Furthermore, even cancer stem cells (CSCs), unipotent cell population presents within the tumor microenvironment, have the ability to alter their metabolism responding to specific bio-energetic and biosynthetic requirements (Deshmukh et al., 2016; Brown et al., 2017). CSCs are key tumor-initiating cells that play an integral role in the metastatic process, and tumor recurrence even after chemotherapy. It is easily understood why CSCs, in the last years, have gained intense interest as a specific target for new therapeutic strategies.

**FIGURE 5:**
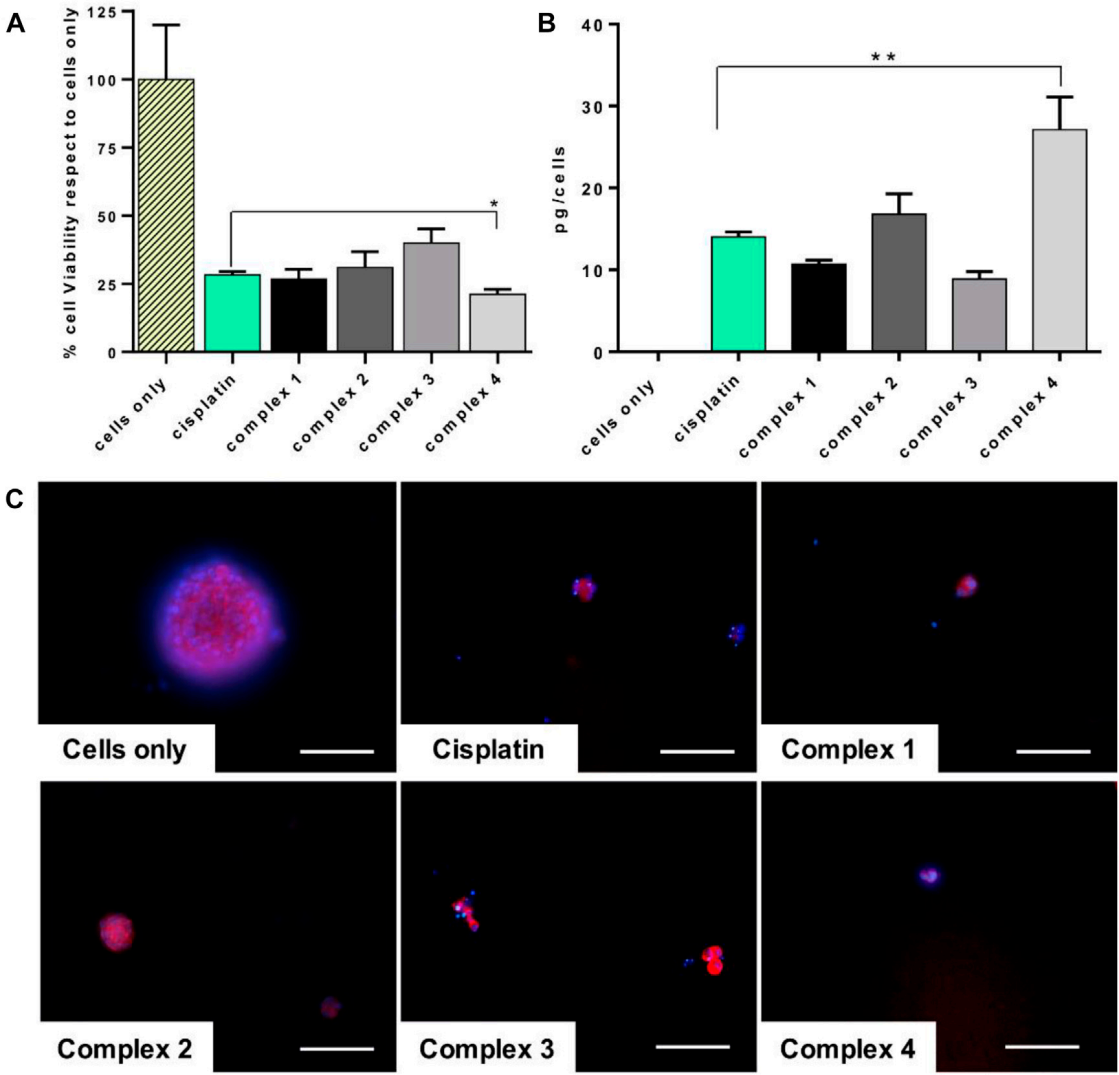
Cisplatin and complexes **1–4** effect on CSCs. CSCs treated for 72 h with 30 µM of drugs. **(A)** MTT assay. Mean ± SEM of the percentage of cell viability respect to cells only (*p value ≤ 0.05). **(B)** ICP-OES analysis. Cellular uptake of picograms of platinum ions (mean ± SEM) per cell are expressed in the graph (**p value ≤ 0.01). **(C)** Actin and DAPI staining of CSCs. F-actin filaments in red; cell nuclei in blue. Scale bars 100 µm.

Based on this evidence, a preliminary *in vitro* study of the tested drug effect on the enriched osteosarcoma stem cell viability and on the drug uptake has been performed. Very promising outcomes indicated that the complex **4** has a statistically significant higher effect on CSCs, *p* value ≤ 0.05 (Figure 5A), strictly related to the observed increased quantity of platinum inside the cells (27.1 pg/cells, *p* value ≤ 0.01), compared to cisplatin (Figure 5B). The morphological evaluation showed a reduction of the dimension of the spheres, typical morphological markers of CSCs, confirming the cytotoxicity results (Figure 5C) (Bouchet and Akhmanova, 2017).

## Conclusion

Four novel glyco-modified Pt(IV) pro-drugs based on cisplatin scaffold were synthesized, linking the sugar moiety and the metal center *via* CUAAC click chemistry. The complexes were tested on a panel of different OS (Osteosarcoma) cell lines and showed very promising activity compared to the reference cisplatin, demonstrating that the presence of a monosaccharide strongly increased the anticancer effect. The complexes resulted also particularly active toward CSCs (Cancer Stem Cells) with the most promising activity shown by complex 4 with a galactose substituent. The interplay of galactose in the metabolism of cancer stem cells is attracting high attention because of potential diagnostic and therapeutic possibilities (Valle et al., 2020; Zheng et al., 2020). At the moment, it is not possible to affirm with certainty if the sugar in the complexes described herein is playing the role of active vector because more specific biological studies (beyond the scope of this work) should be conducted (i.e., inhibition of the GLUTs receptors), but it is clear that the presence of the sugar is increasing the anticancer activity and the drug internalization, as demonstrated with the uptake experiment in CSCs (this could be due to the higher lipophilicity). All the complexes showed very promising activity but the discrimination between glucose and galactose and between the two linkers is not evident yet and this could confirm the hypothesis that these species are internalized by passive diffusion. Ideally, the sugars should be deprotected in order to be better recognized by the receptors, and our group is working in this direction, even if the synthesis is not trivial. We are planning to speculate on the role of the sugar scaffold by developing analogous complexes where the carbohydrate is conjugated *via* the C2 carbon (not the anomeric carbon) that was demonstrated to be the best in terms of cellular recognition (Patra et al., 2016). Finally, we are conjugating these species, through specific linkers, to magnetic nanoparticles that will act as a delivery platform to increase the drug selectivity and cellular internalization.

## Data Availability

The original contributions presented in the study are included in the article/Supplementary Material, further inquiries can be directed to the corresponding authors.
