# Supplementary material for: Click Pt(IV)-Carbohydrates Pro-Drugs for Treatment of Osteosarcoma
Source: Front Chem. 2021 Dec 7;9:795997. doi: 10.3389/fchem.2021.795997 (PMC8688915; doi:10.3389/fchem.2021.795997)
Supplement: Supplementary file 1 [file DataSheet1.pdf]

Supporting Information

Click Pt(IV)-carbohydrates pro-drugs for treatment of osteosarcoma

Eoin Moynihan, Giada Bassi, Andrea Ruffini, Silvia Panseri, Monica Montesi[2] Trinidad Velasco Torrijos, Diego Montagner

NMR, HR-MS and HPLC

Compound 9

^1H NMR

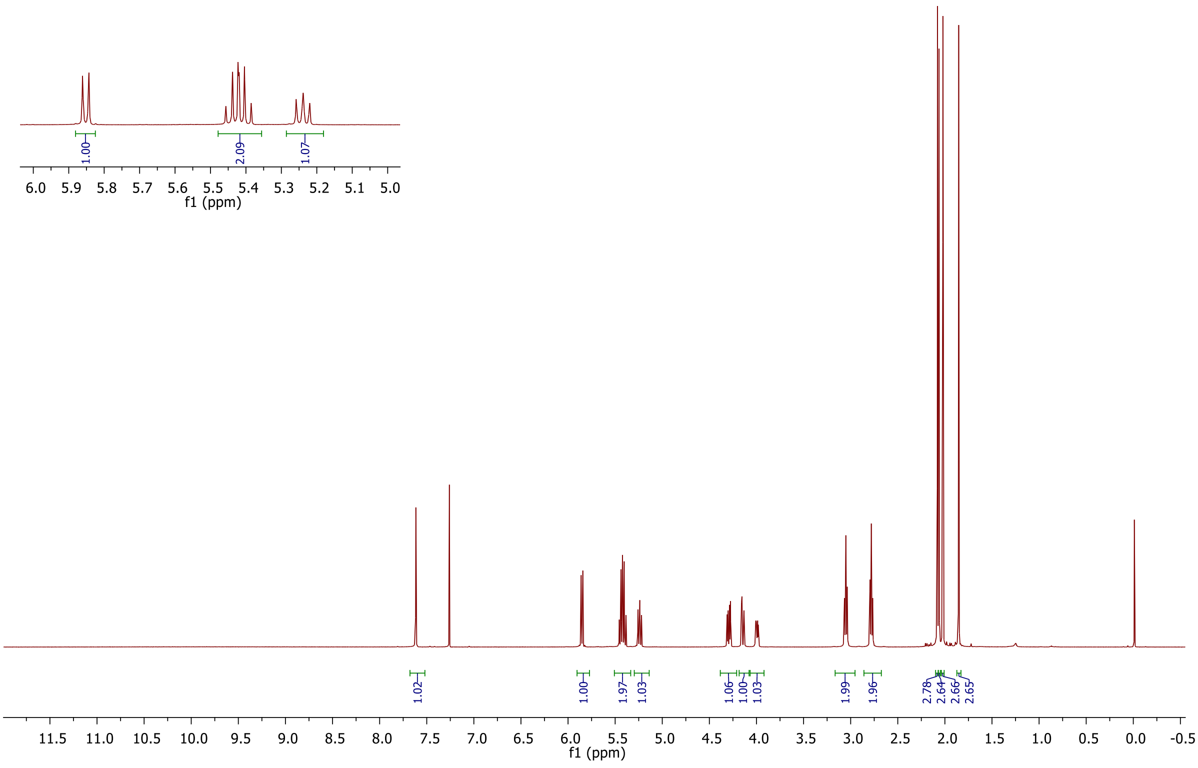

^{13}C NMR

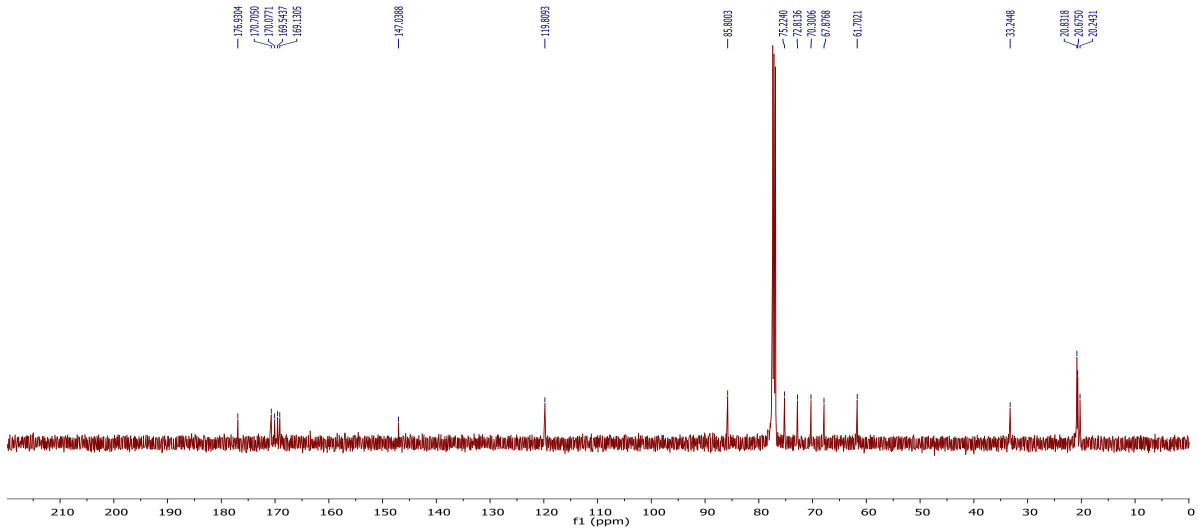

176.9304
170.7050
170.0771
169.5437
169.1305
147.0388
119.8093
85.8003
75.2240
72.8136
70.3006
67.8768
61.7021
33.2448
20.8318
20.6750
20.2431
f1 (ppm)

COSY

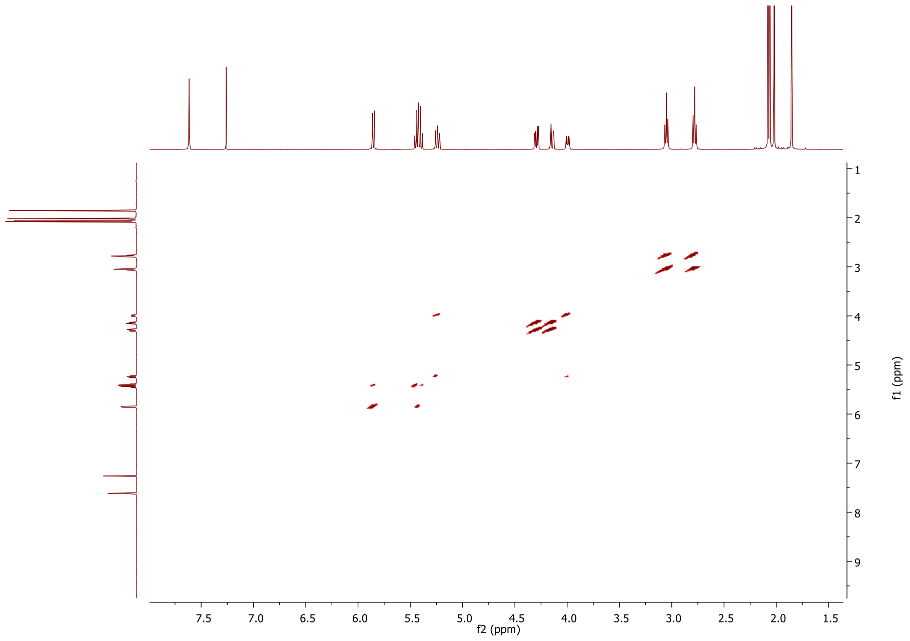

f2 (ppm)
f1 (ppm)

HSQC

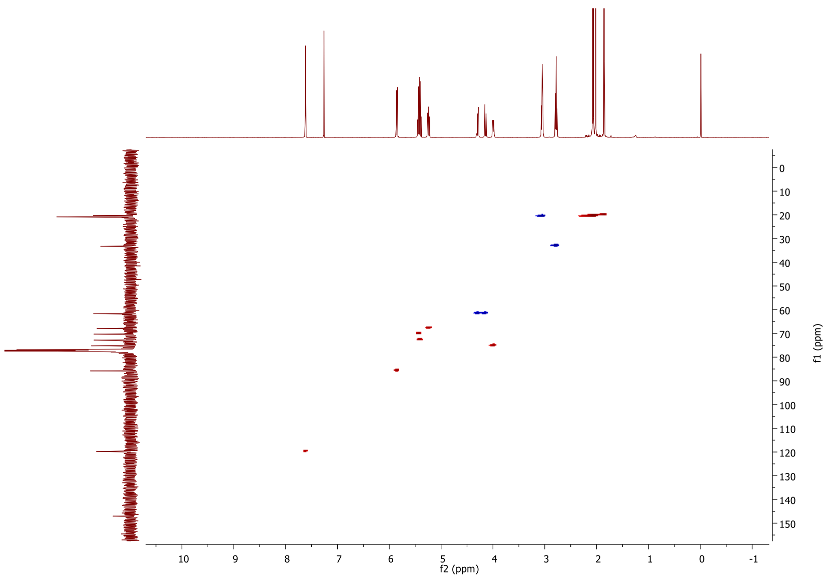

HMBC

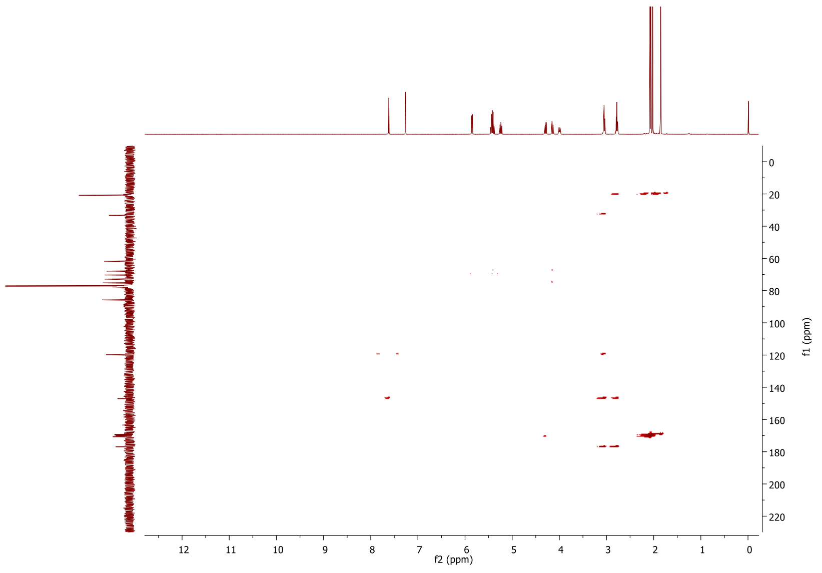

HR-MS

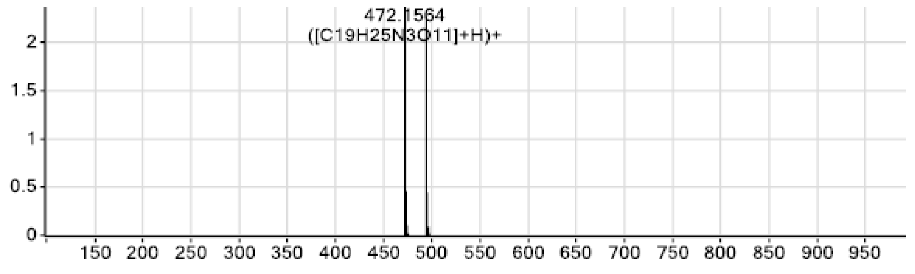

Compound 10

^{1}H NMR

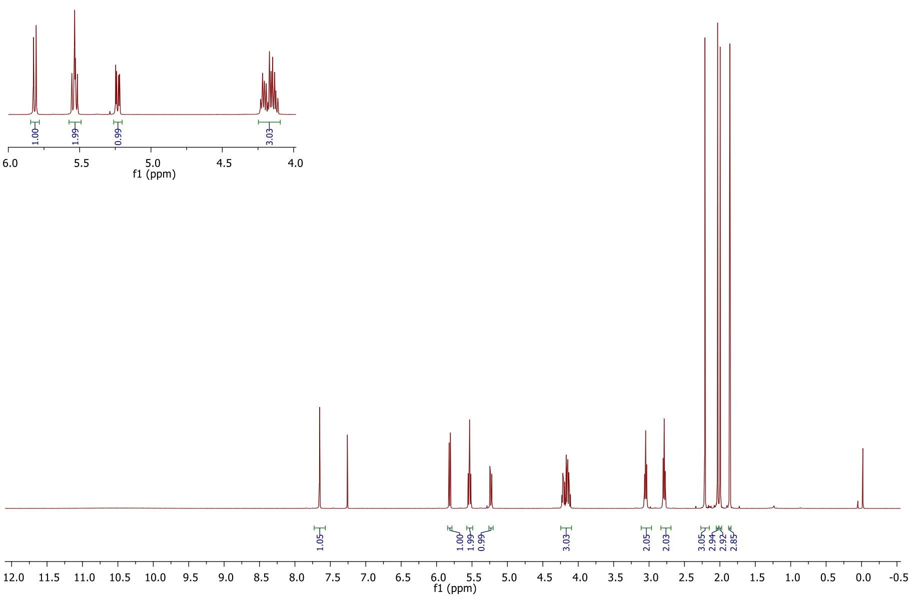

^{13}C NMR

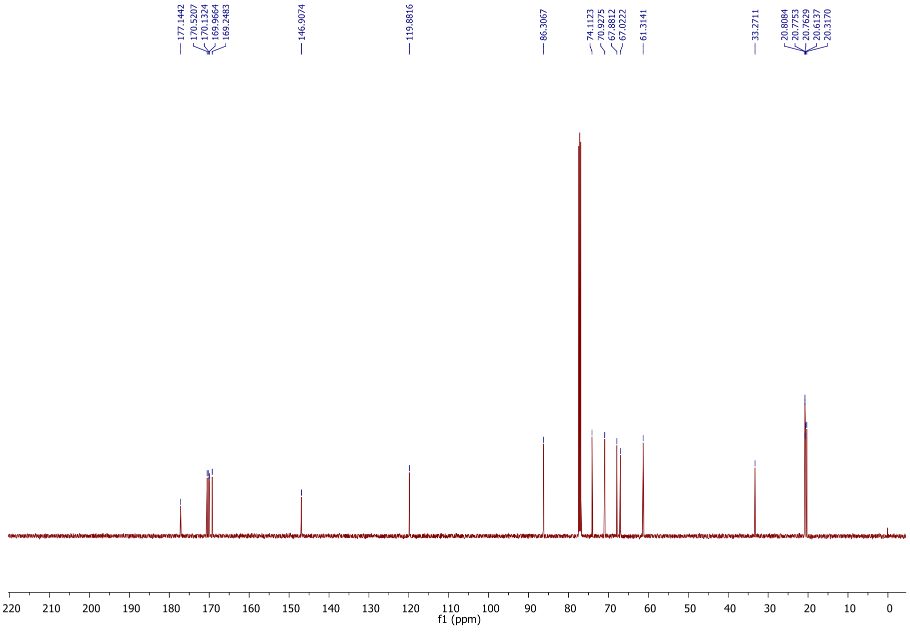

COSY

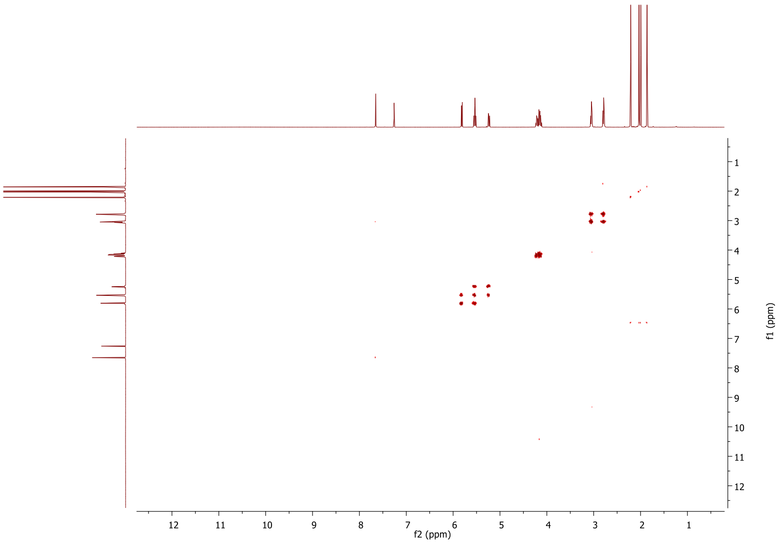

HSQC

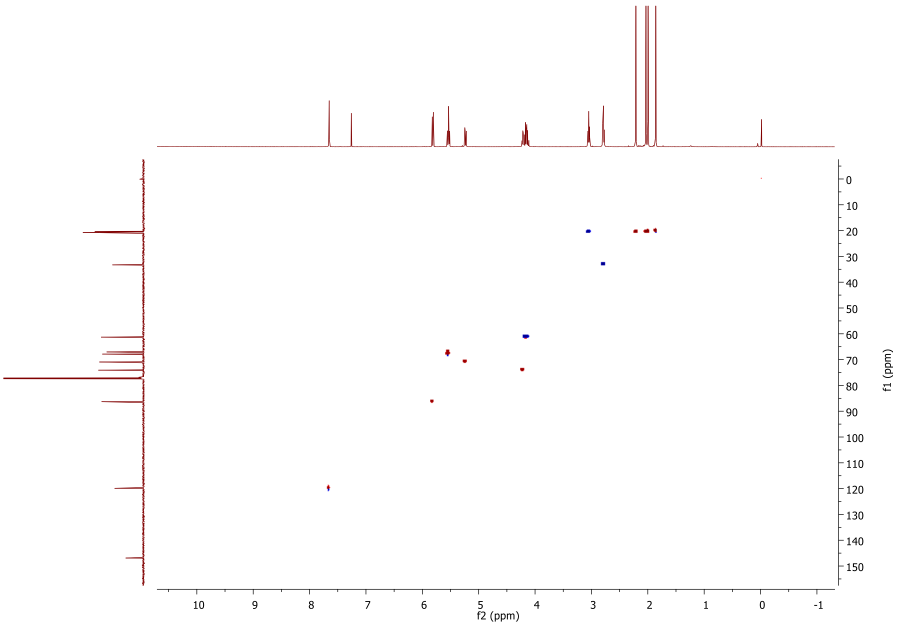

HMBC

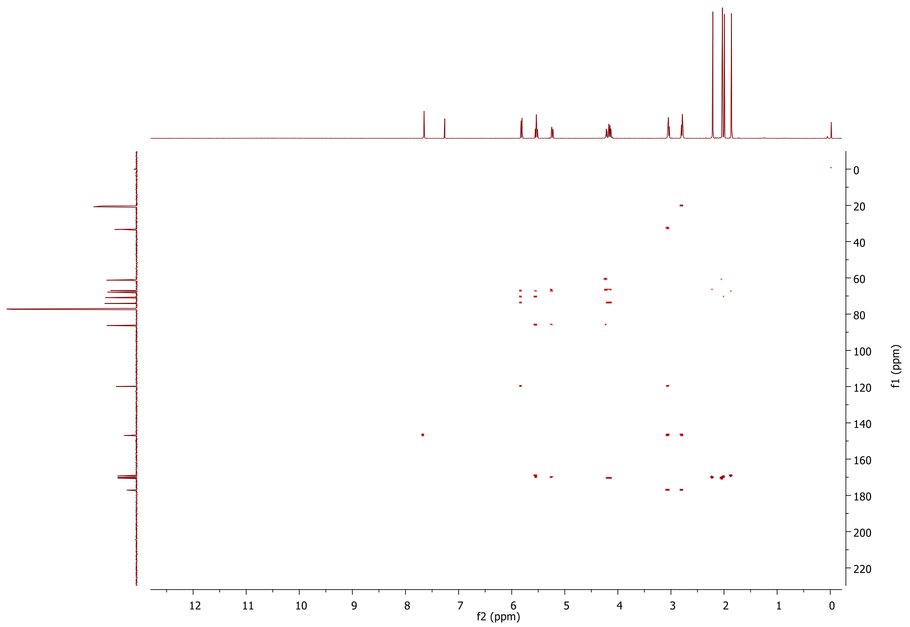

12
11
10
9
8
7
6
5
4
3
2
1
0
f2 (ppm)
0
20
40
60
80
100
120
140
160
180
200
220
f1 (ppm)

HR-MS

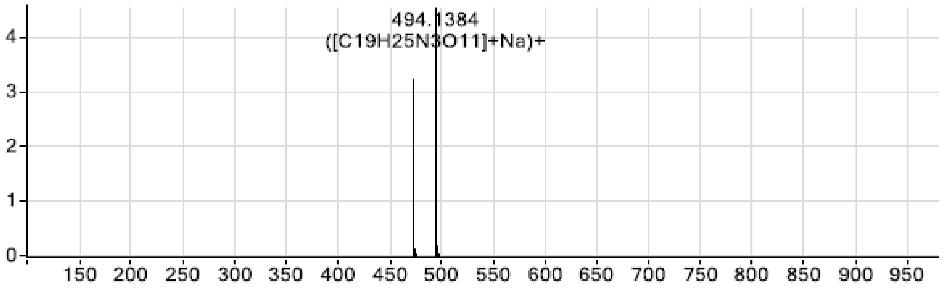

494.1384
([C19H25N3O11]+Na)+
4
3
2
1
0
150
200
250
300
350
400
450
500
550
600
650
700
750
800
850
900
950

Compound 17

^{1}H NMR

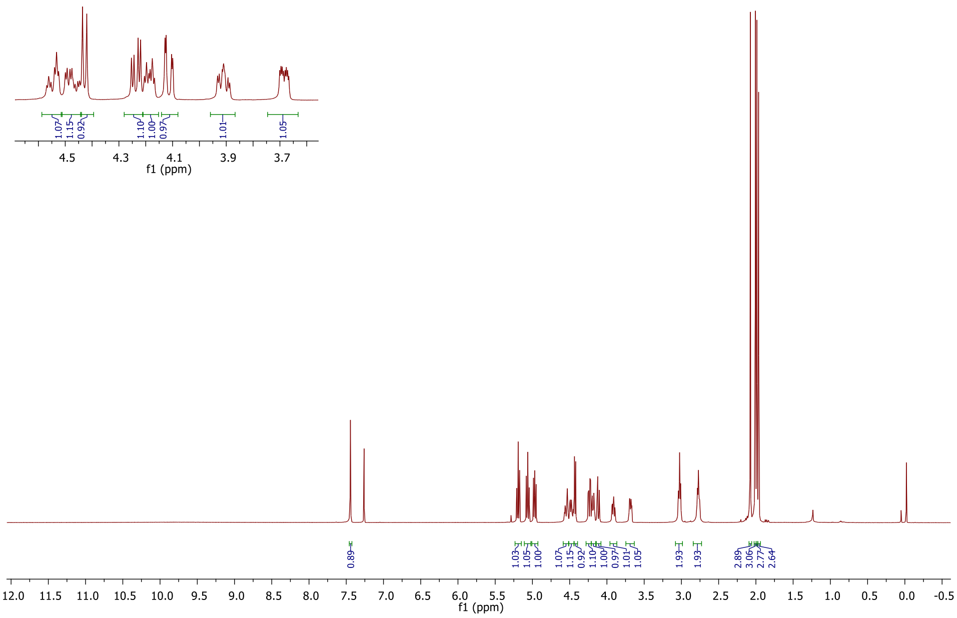

^{13}C NMR

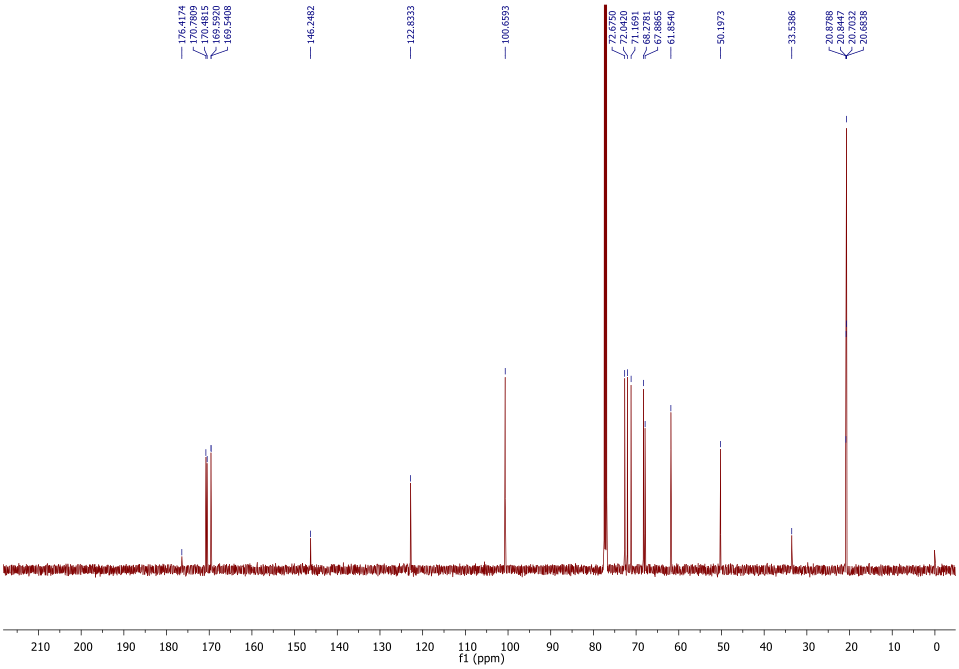

COSY

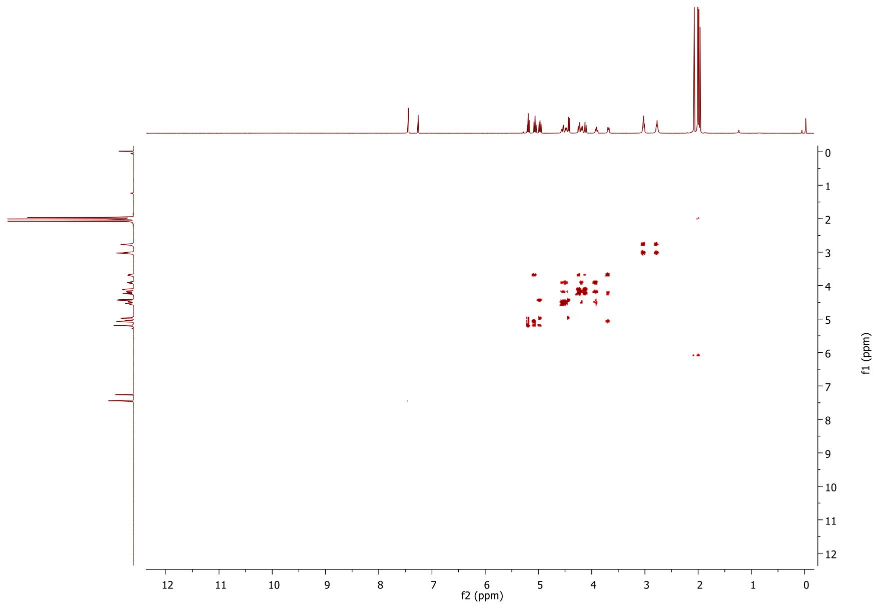

HSQC

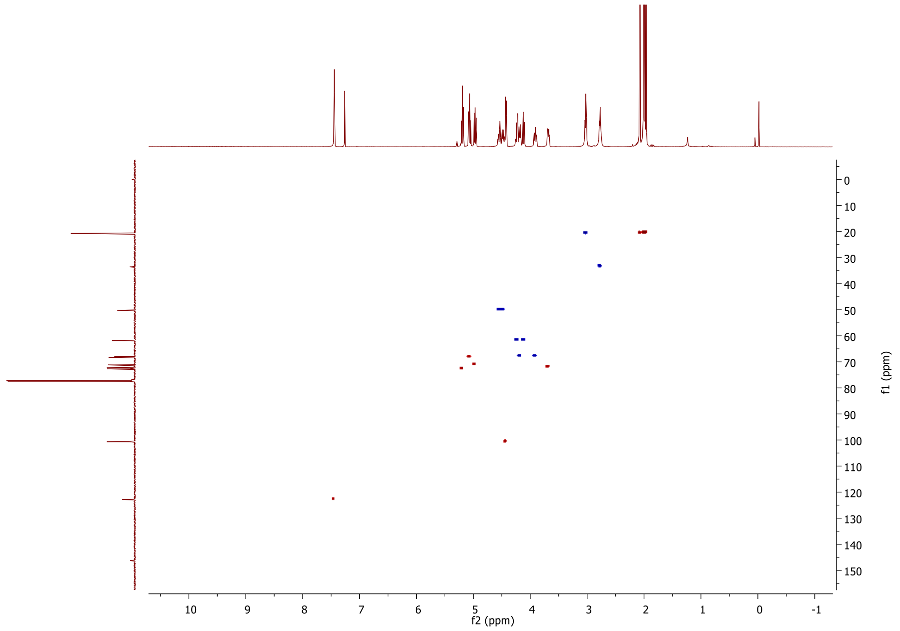

HMBC

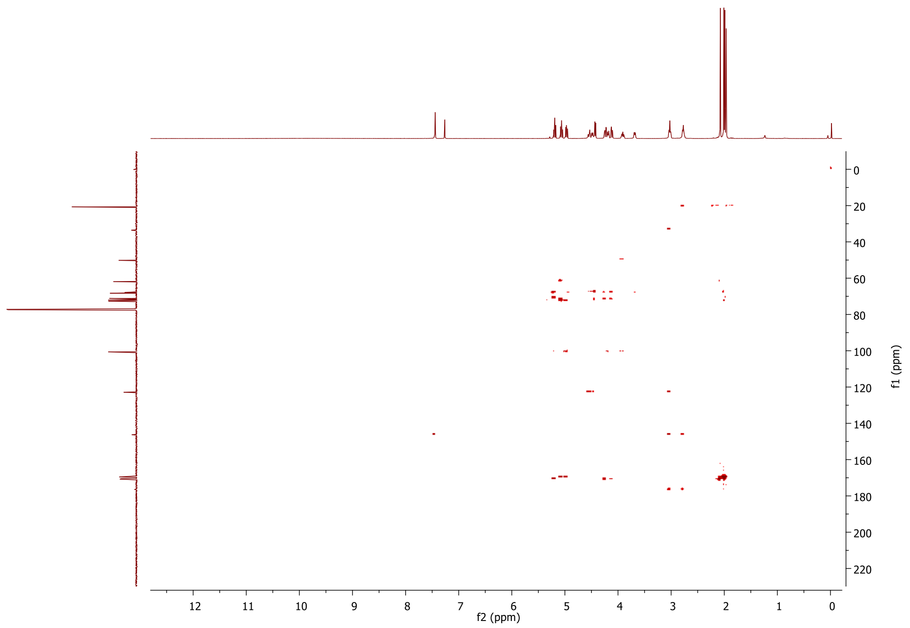

HR-MS

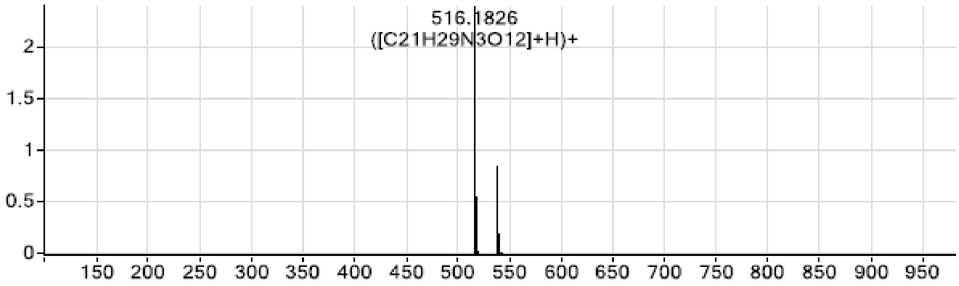

Compound 18

^{1}H NMR

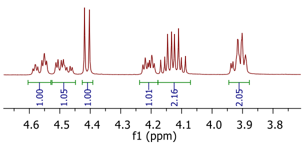

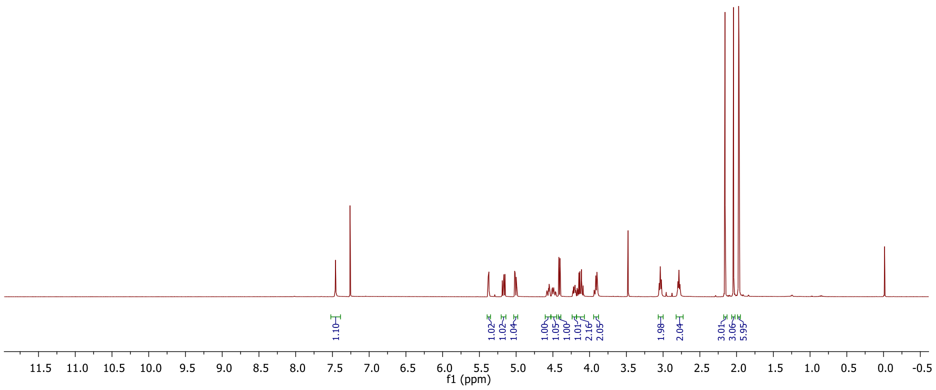

^{13}C NMR

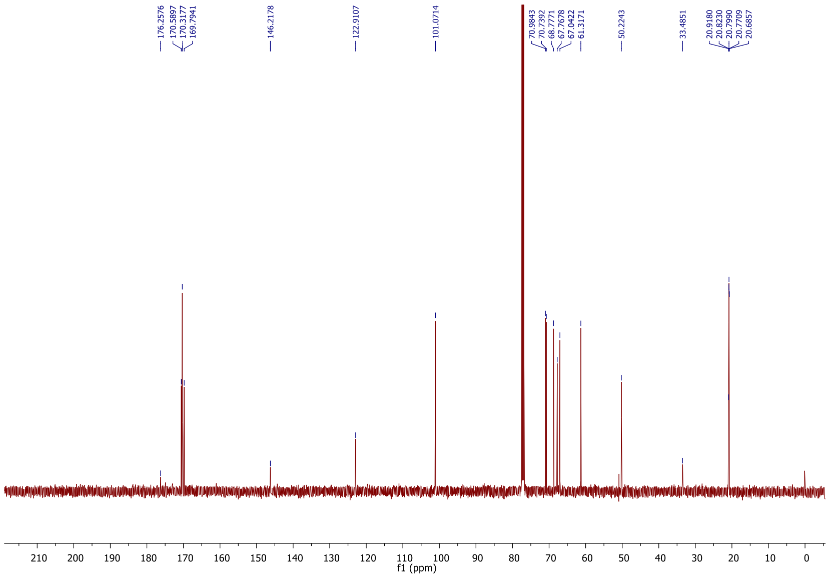

COSY

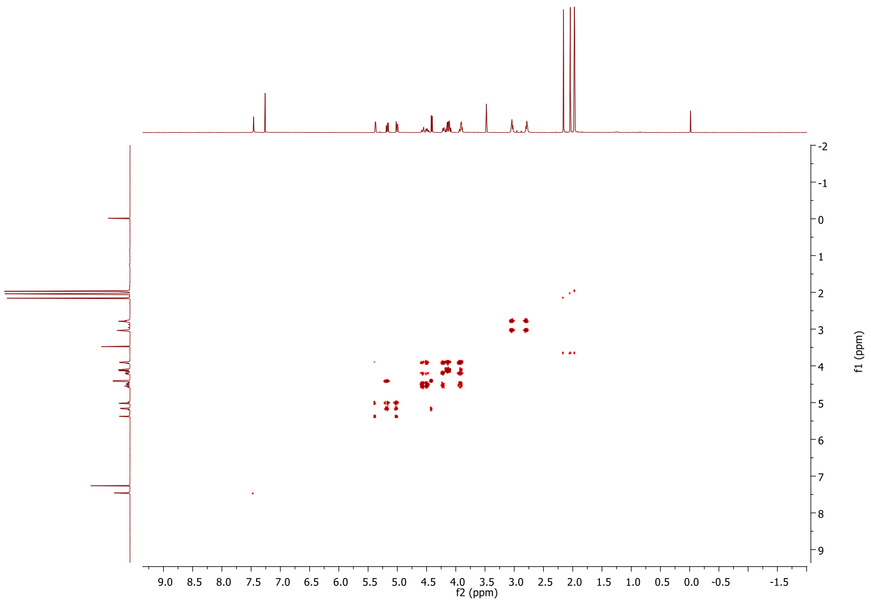

HSQC

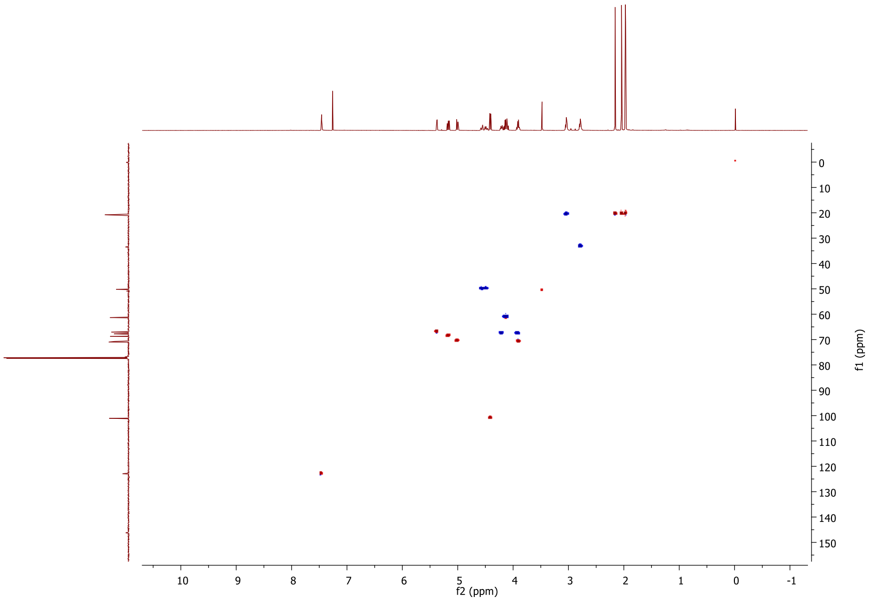

HMBC

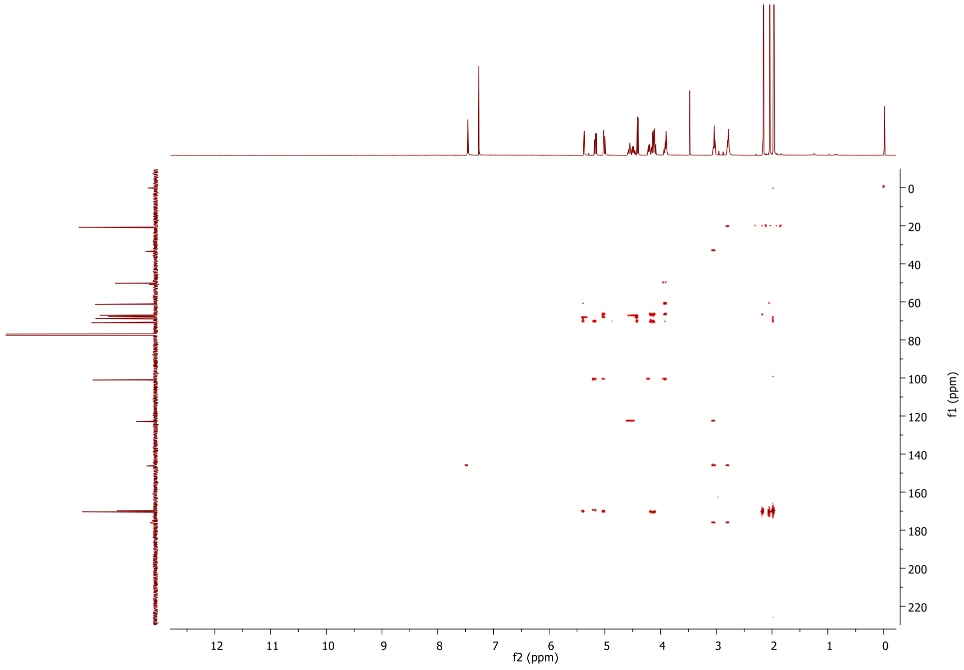

HR-MS

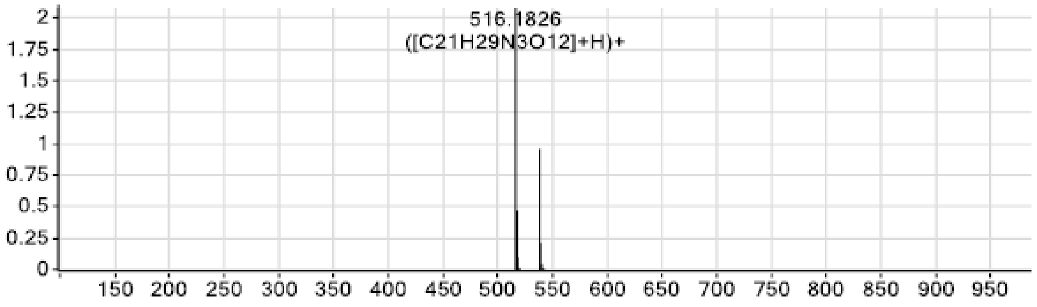

Compound 11

^{1}H NMR

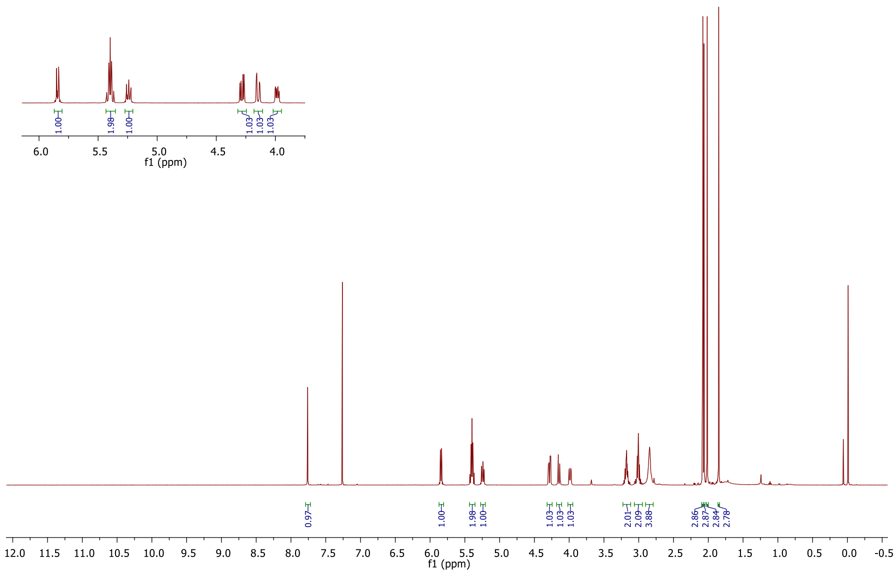

^{13}C NMR

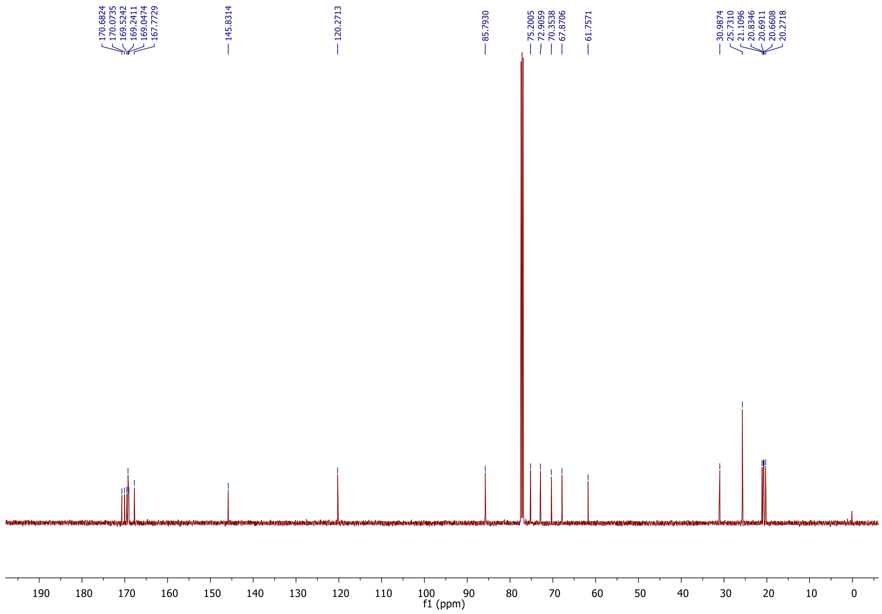

COSY

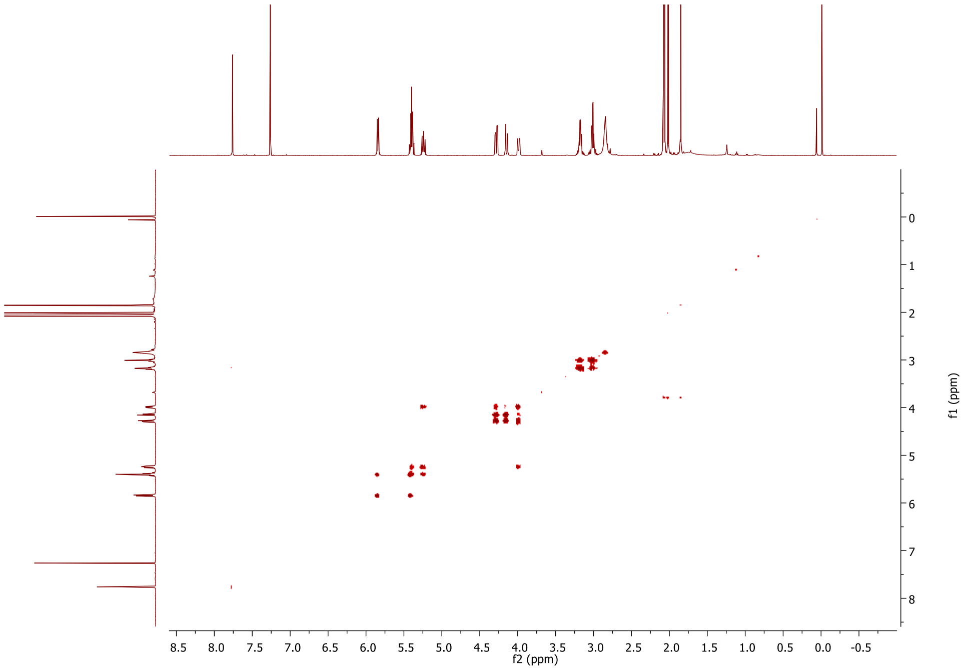

8.5 8.0 7.5 7.0 6.5 6.0 5.5 5.0 4.5 4.0 3.5 3.0 2.5 2.0 1.5 1.0 0.5 0.0 -0.5
f2 (ppm)
0 1 2 3 4 5 6 7 8
f1 (ppm)

HSQC

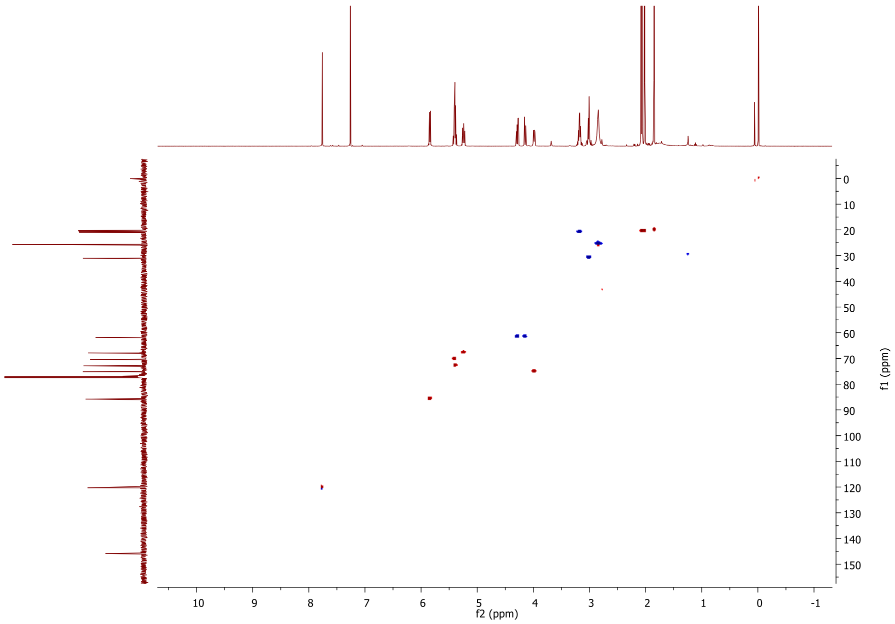

10 9 8 7 6 5 4 3 2 1 0 -1
f2 (ppm)
0 10 20 30 40 50 60 70 80 90 100 110 120 130 140 150
f1 (ppm)

HMBC

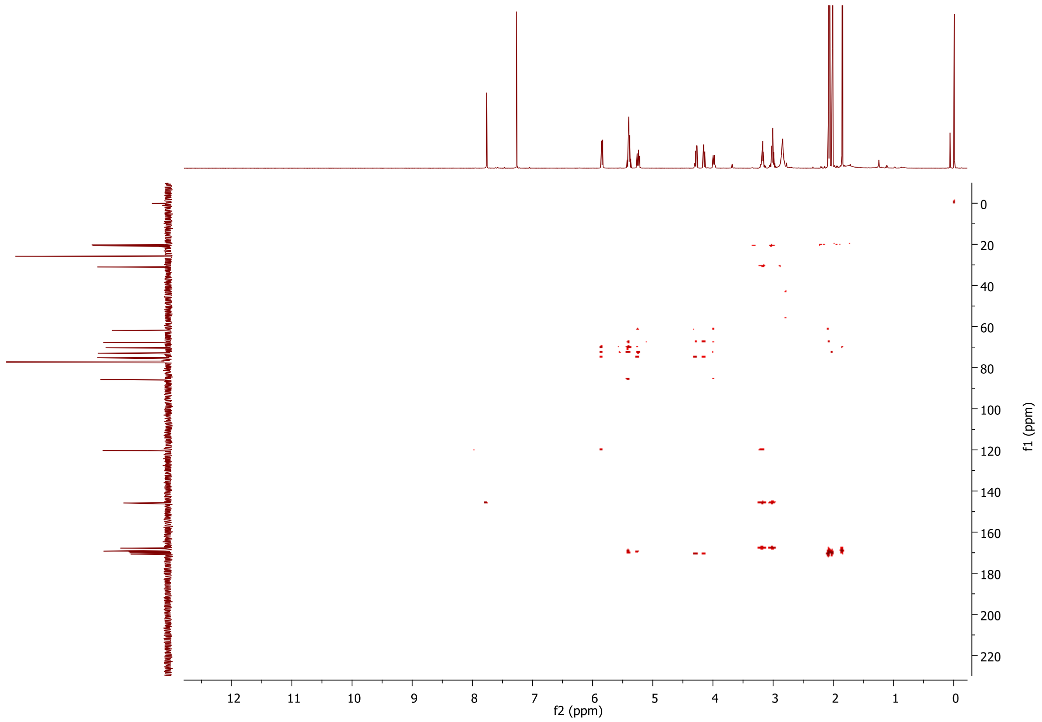

HR-MS

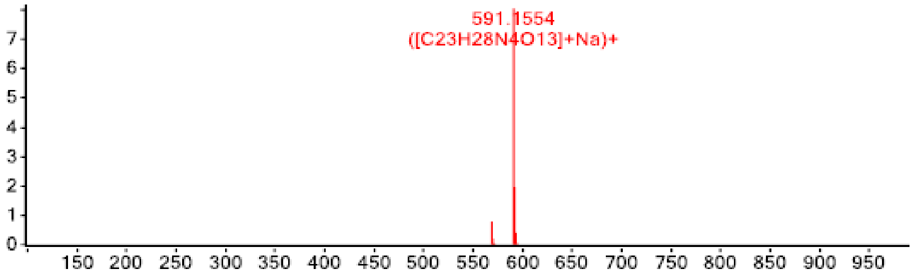

Compound 12

^{1}H NMR

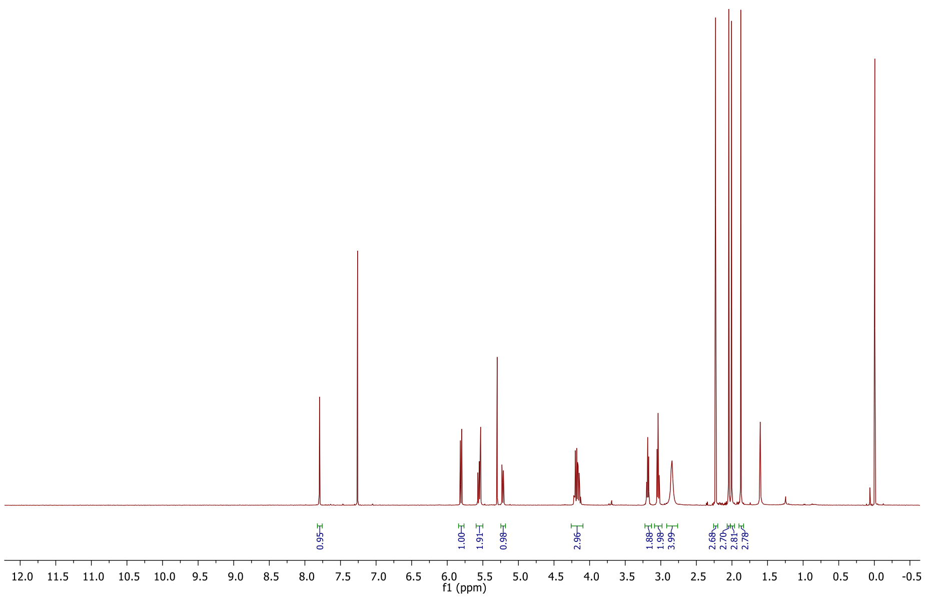

^{13}C NMR

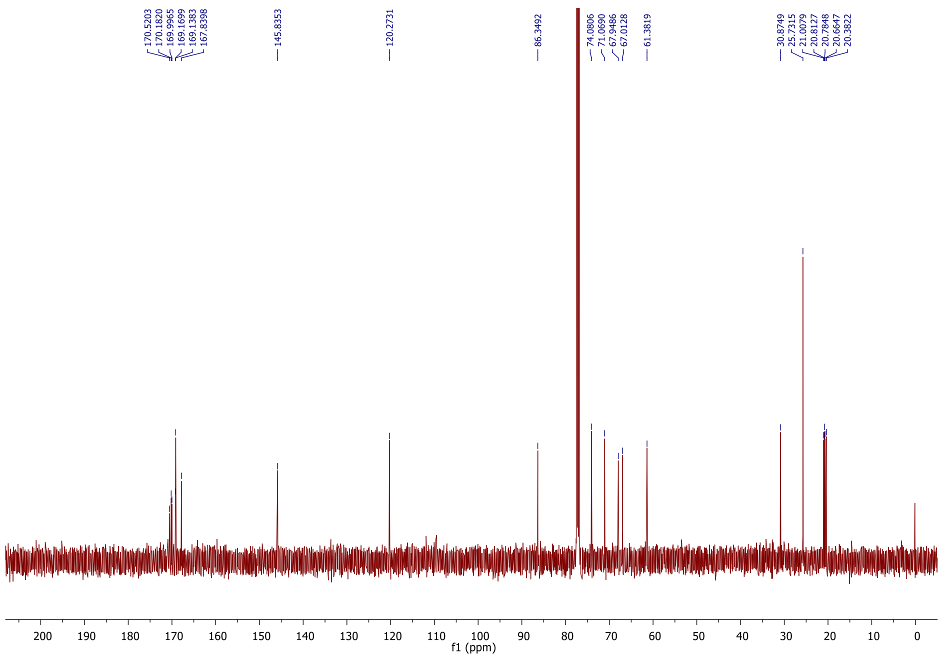

COSY

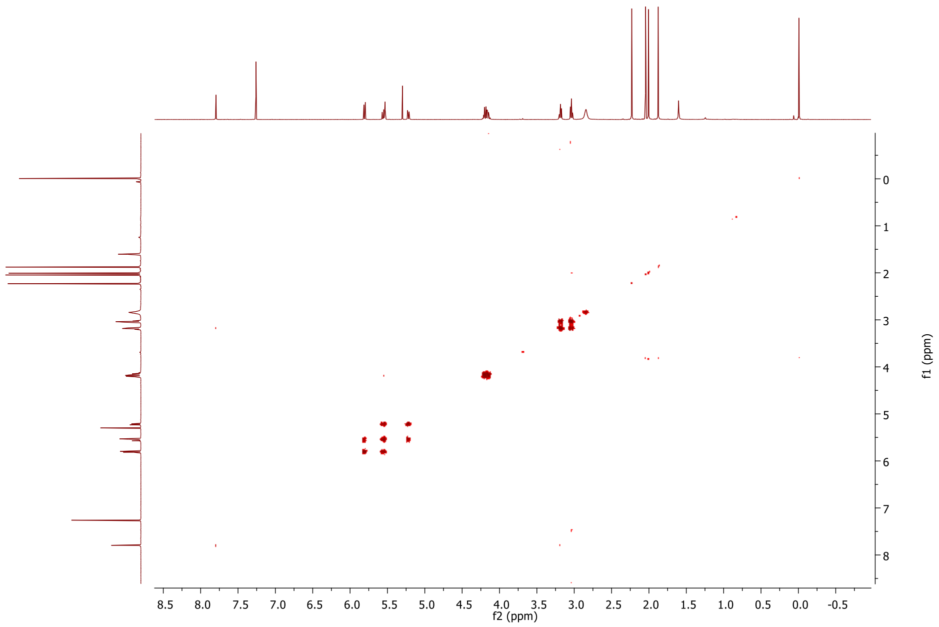

8.5 8.0 7.5 7.0 6.5 6.0 5.5 5.0 4.5 4.0 3.5 3.0 2.5 2.0 1.5 1.0 0.5 0.0 -0.5
f2 (ppm)
0 1 2 3 4 5 6 7 8
f1 (ppm)

HSQC

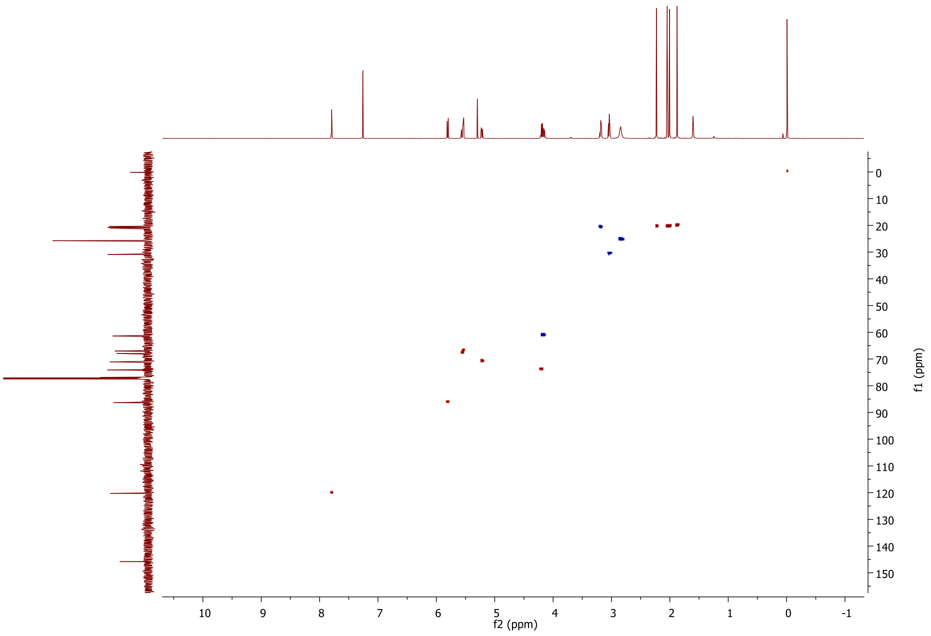

10 9 8 7 6 5 4 3 2 1 0 -1
f2 (ppm)
0 10 20 30 40 50 60 70 80 90 100 110 120 130 140 150
f1 (ppm)

HMBC

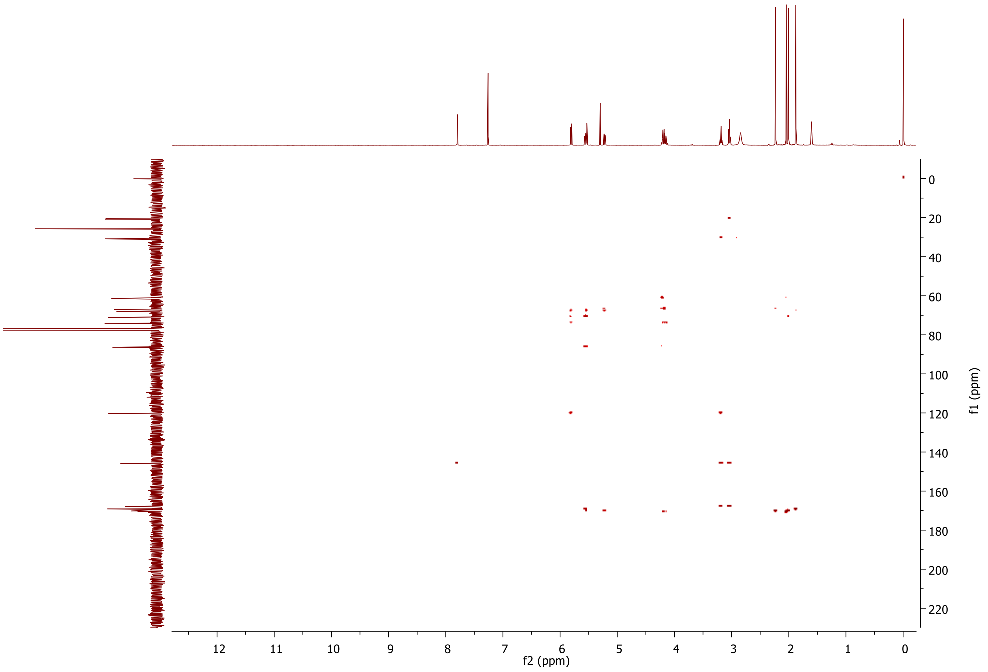

HR-MS

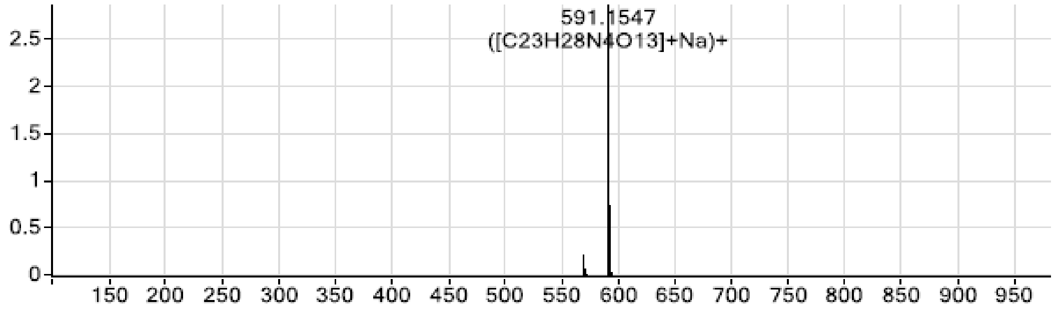

Compound 19

^{1}H NMR

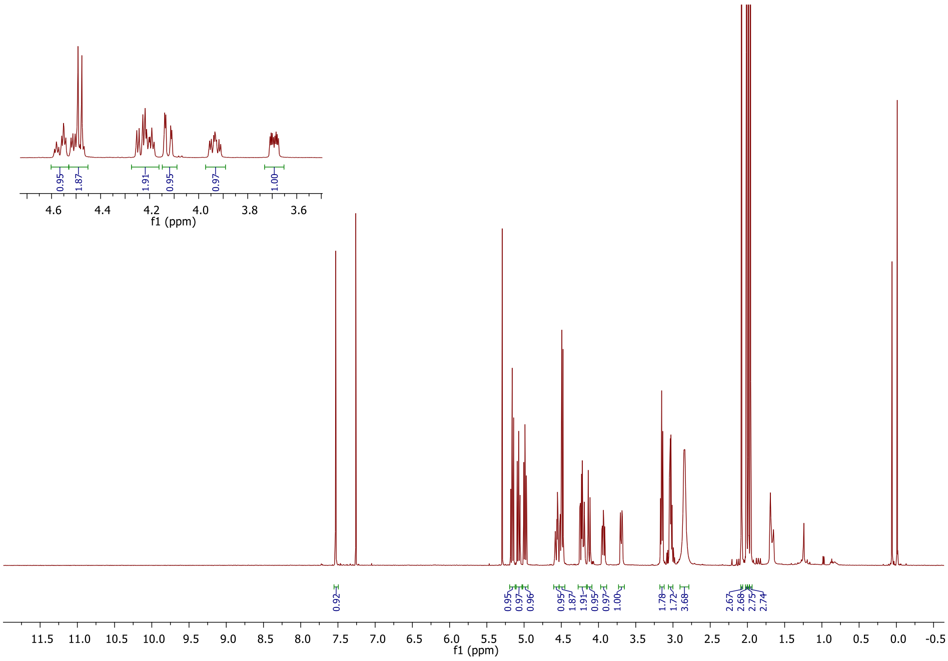

^{13}C NMR

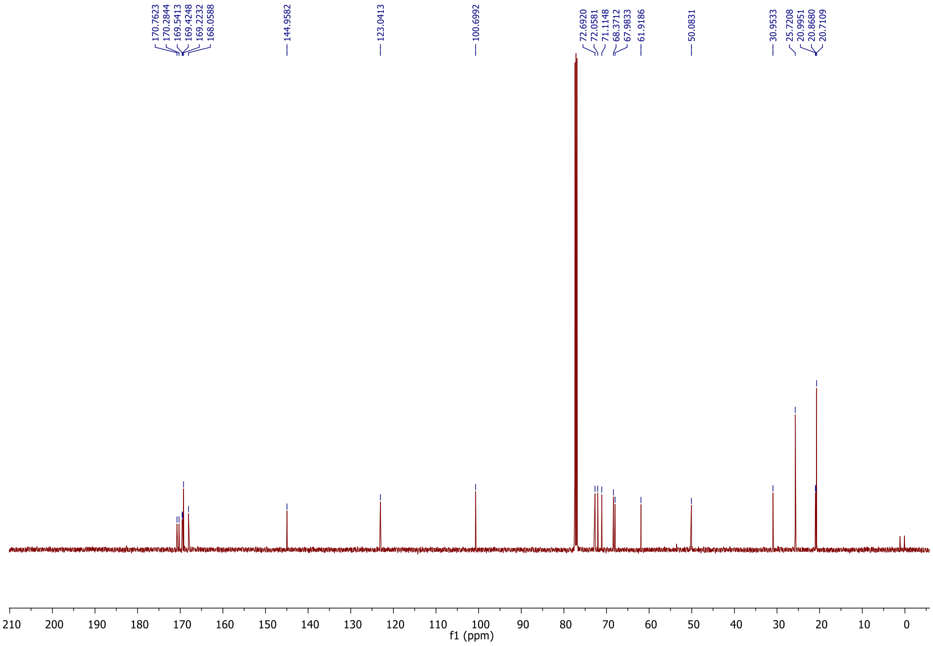

COSY

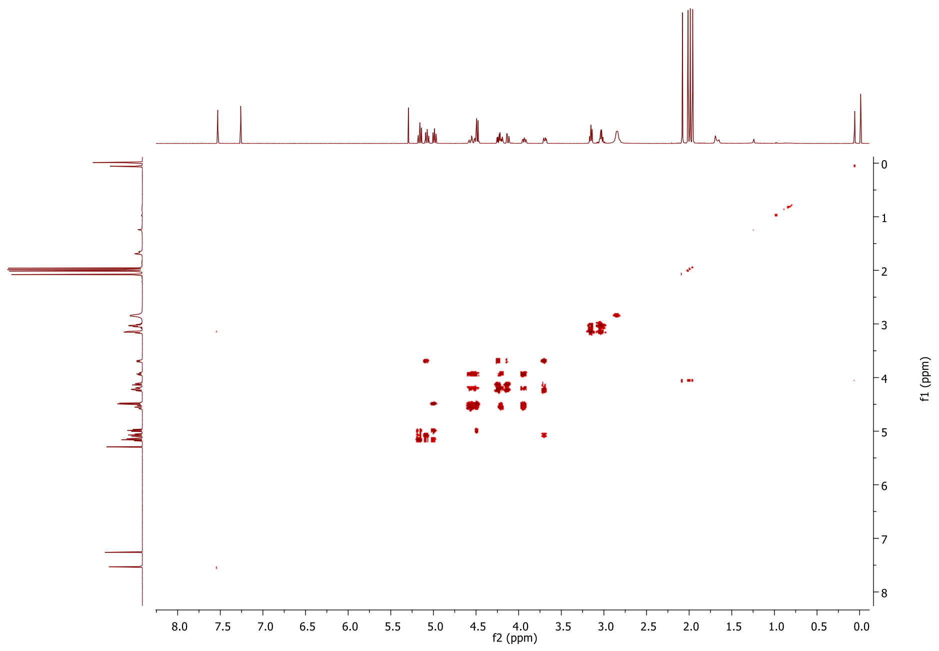

8.0
7.5
7.0
6.5
6.0
5.5
5.0
4.5
4.0
3.5
3.0
2.5
2.0
1.5
1.0
0.5
0.0
f2 (ppm)
0
1
2
3
4
5
6
7
8
f1 (ppm)

HSQC

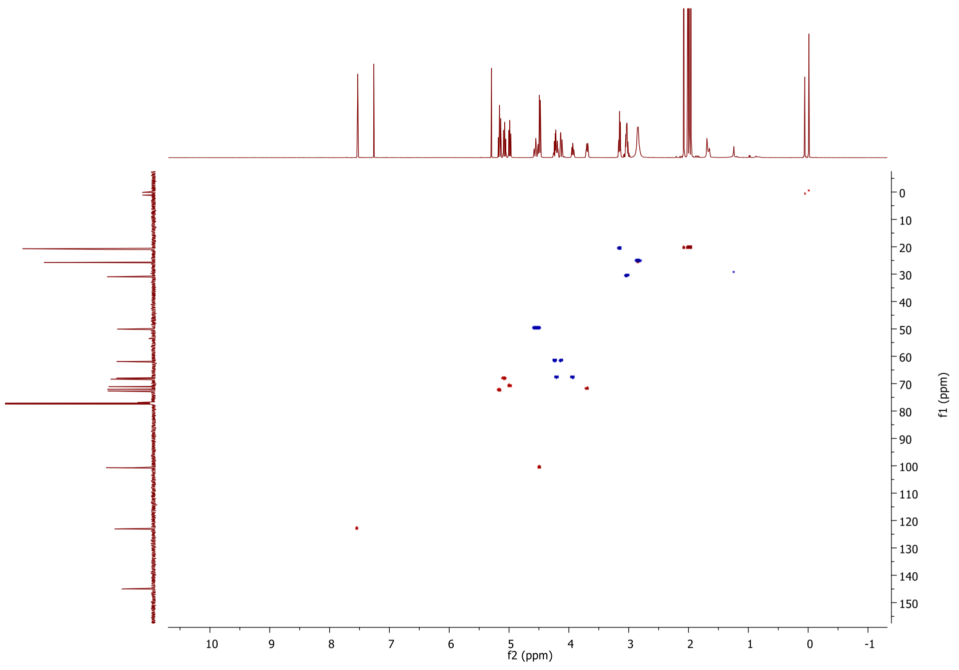

10
9
8
7
6
5
4
3
2
1
0
-1
f2 (ppm)
0
10
20
30
40
50
60
70
80
90
100
110
120
130
140
150
f1 (ppm)

HMBC

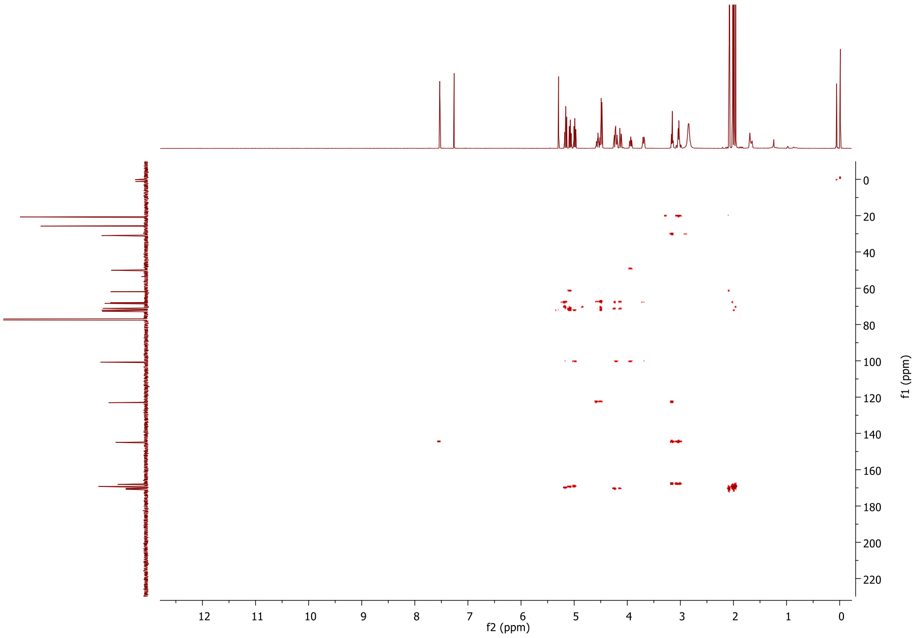

HR-MS

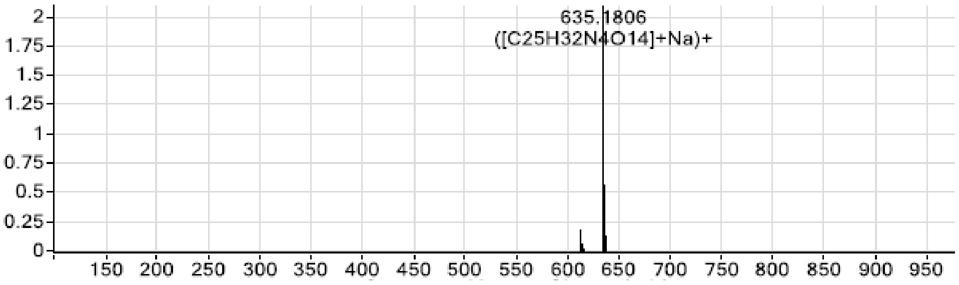

Compound 20

^{1}H NMR

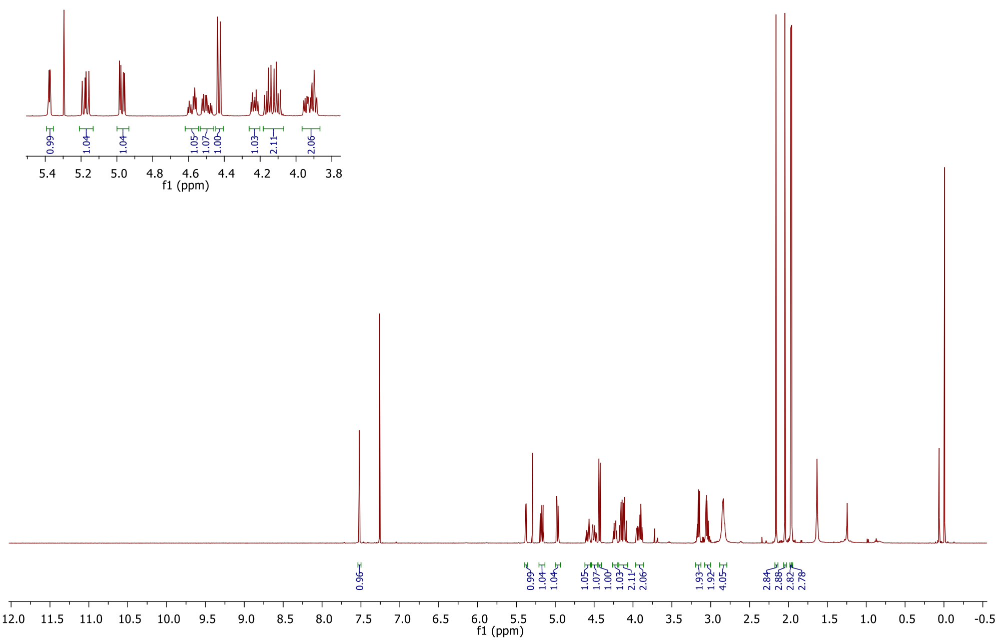

^{13}C NMR

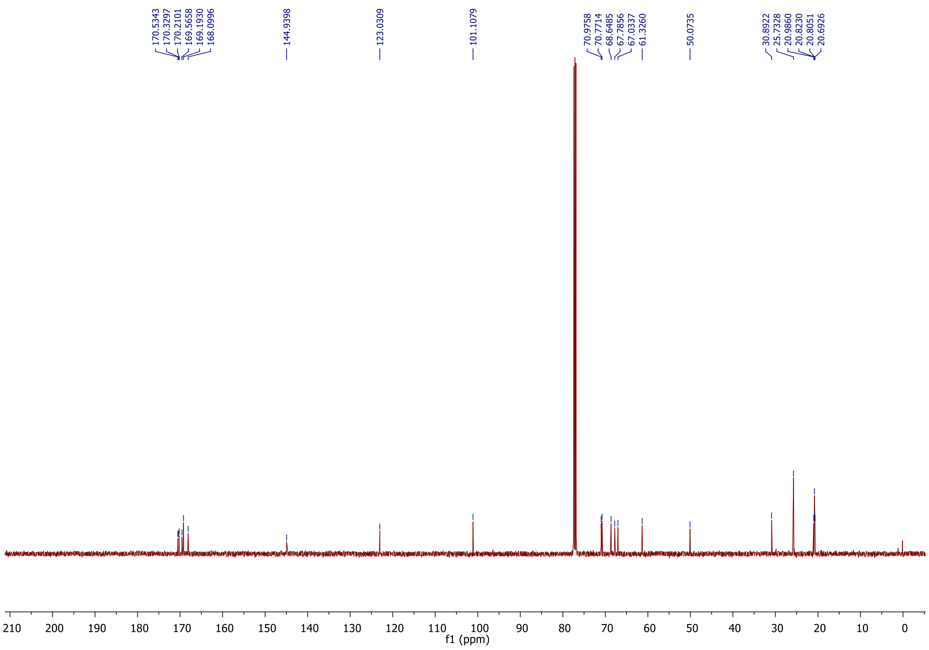

COSY

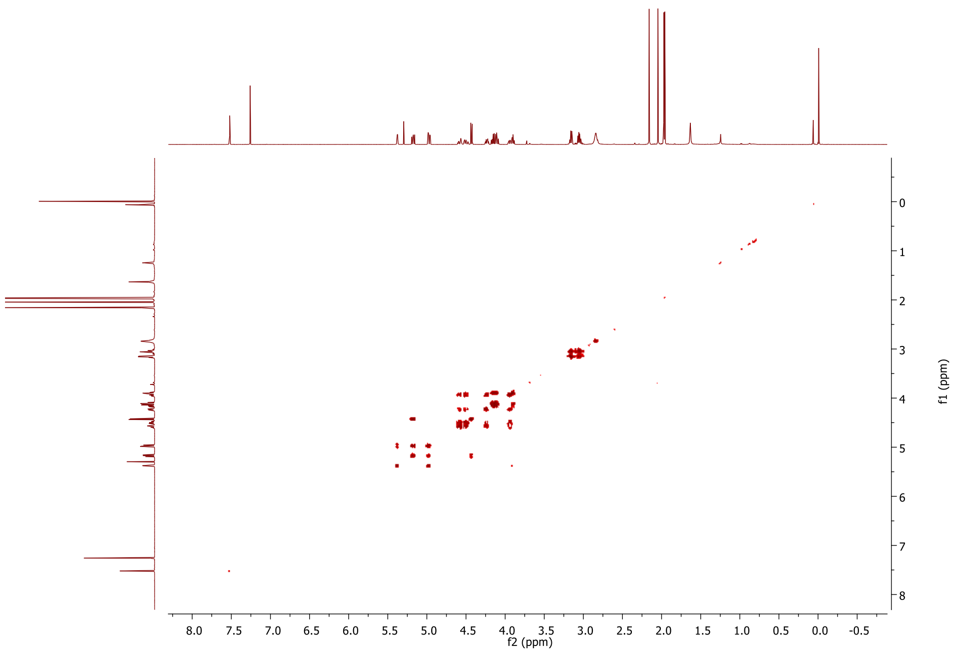

HSQC

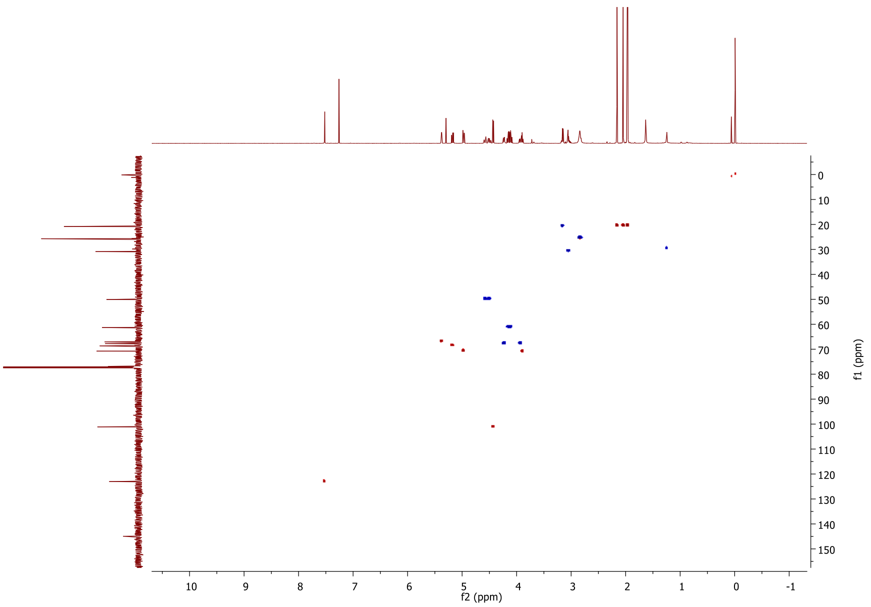

HMBC

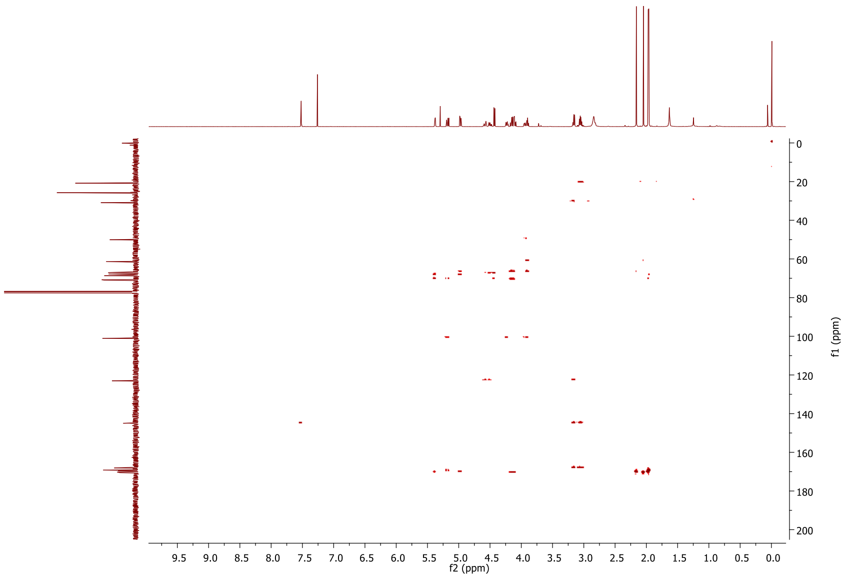

HR-MS

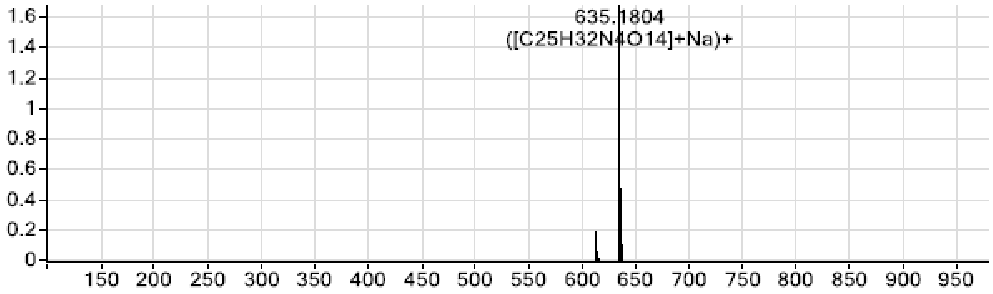

Complex 1

^{1}H NMR

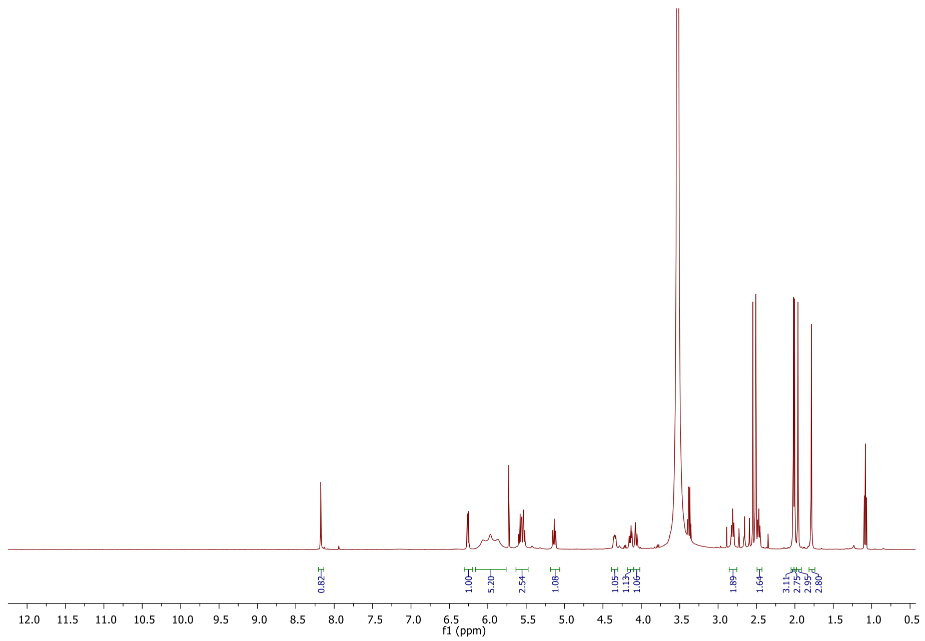

^{13}C NMR

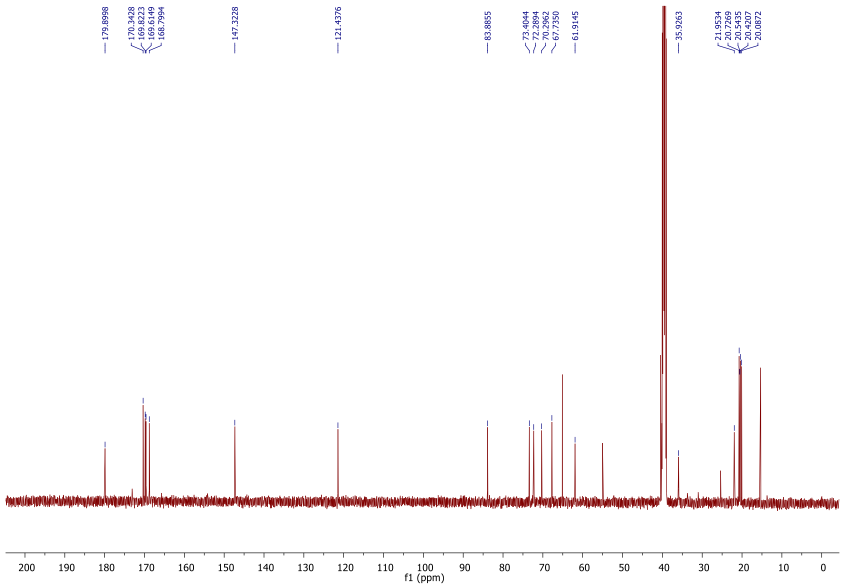

COSY

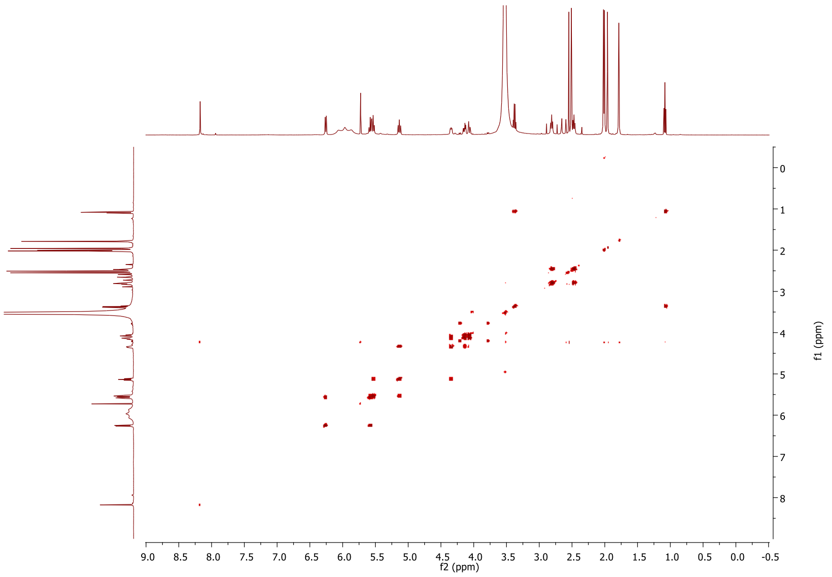

9.0 8.5 8.0 7.5 7.0 6.5 6.0 5.5 5.0 4.5 4.0 3.5 3.0 2.5 2.0 1.5 1.0 0.5 0.0 -0.5
f2 (ppm)
0 1 2 3 4 5 6 7 8
f1 (ppm)

HSQC

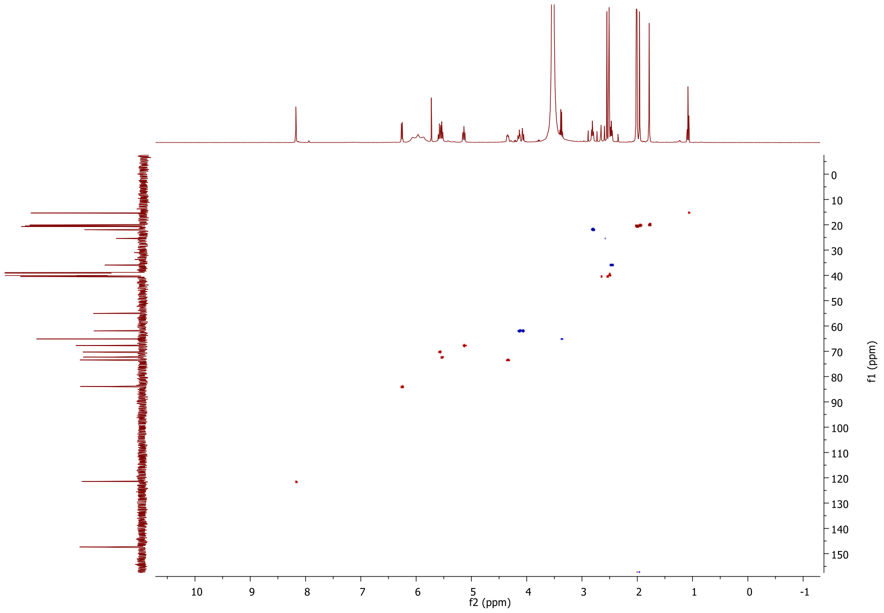

10 9 8 7 6 5 4 3 2 1 0 -1
f2 (ppm)
0 10 20 30 40 50 60 70 80 90 100 110 120 130 140 150
f1 (ppm)

HMBC

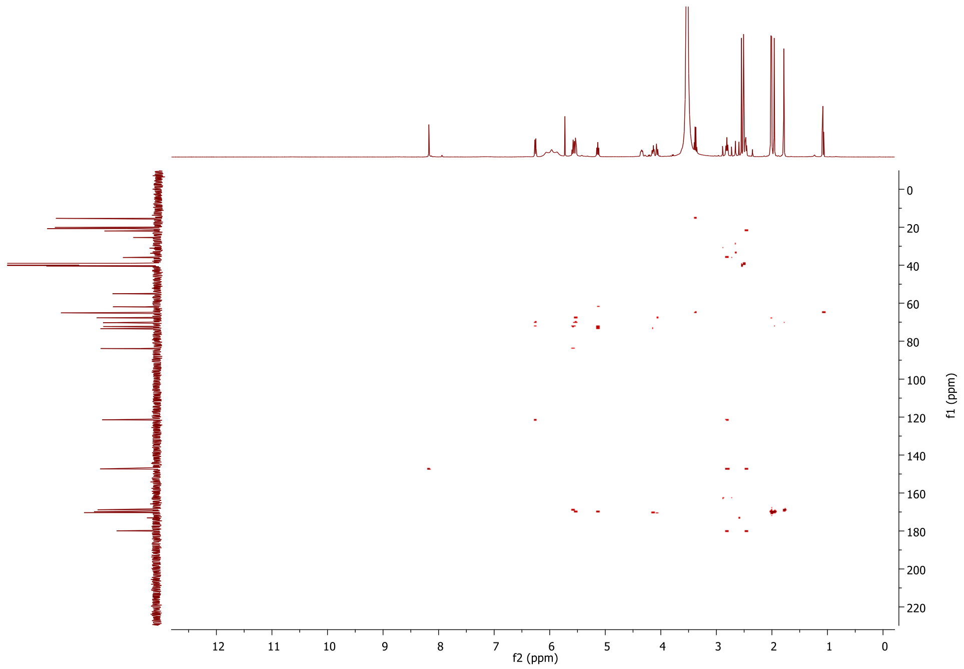

^{195}Pt NMR

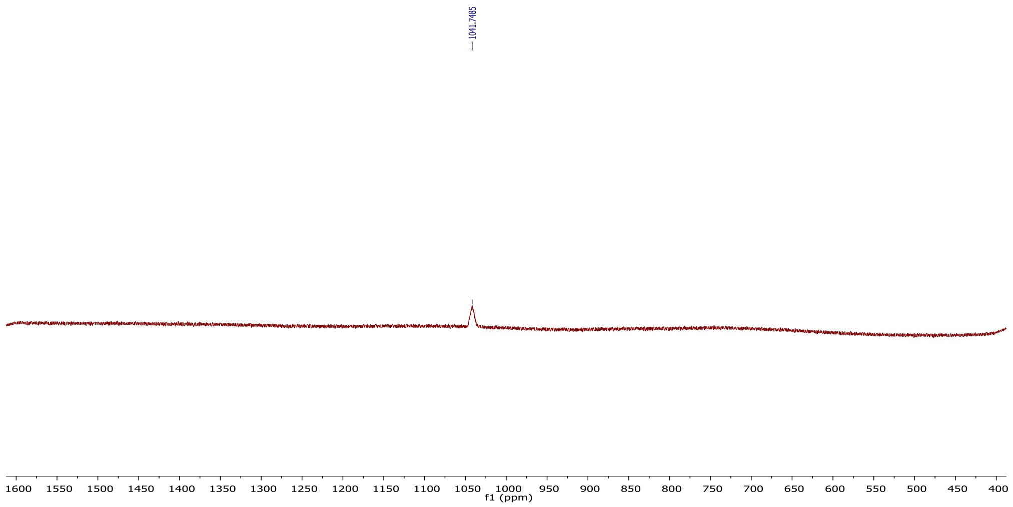

HR-MS

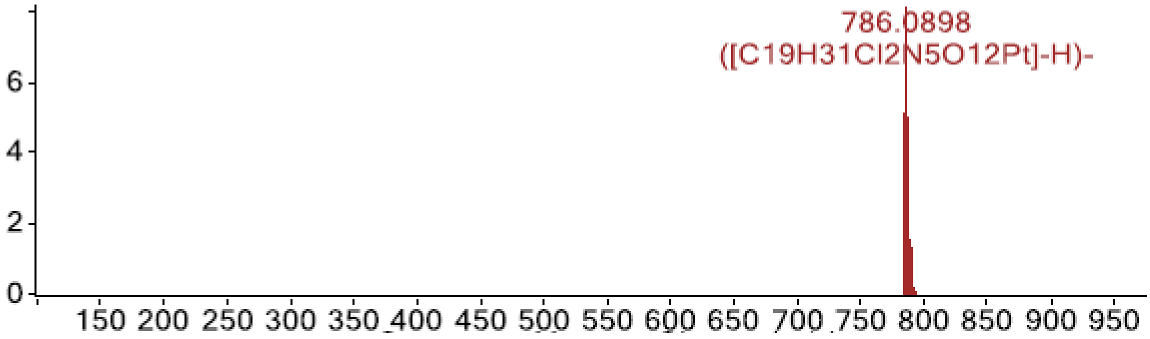

Complex 2

^{1}H NMR

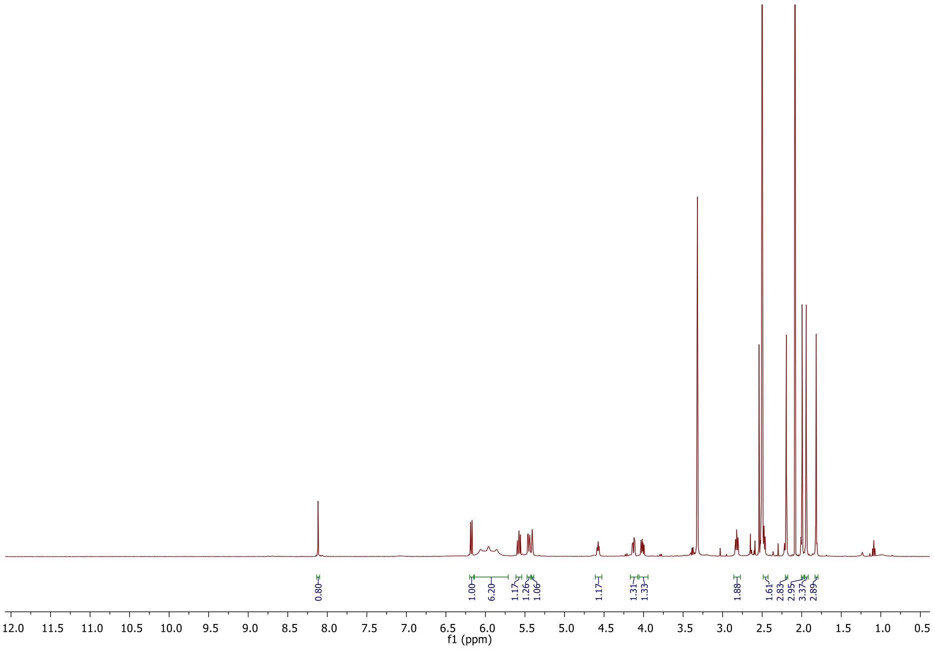

^{13}C NMR

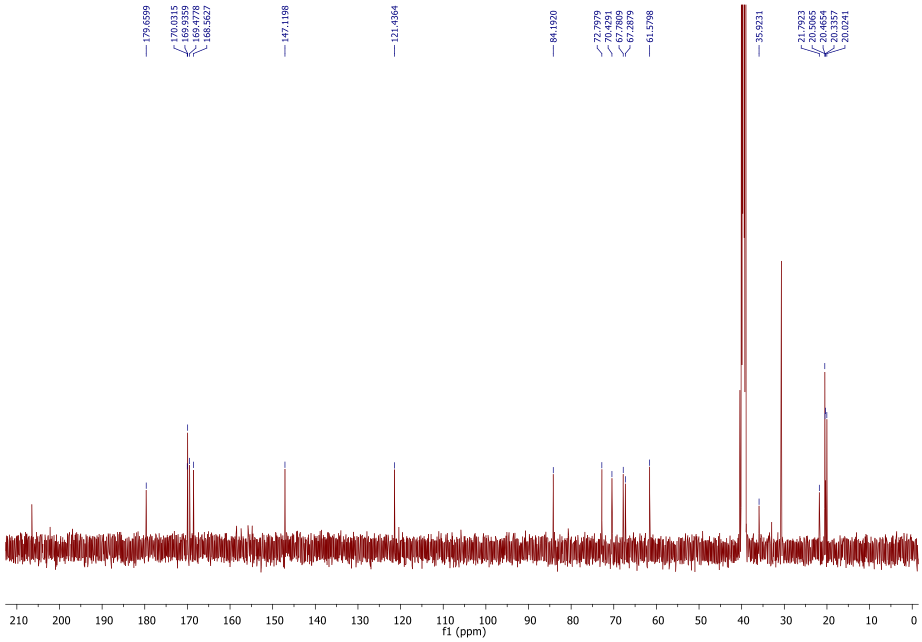

COSY

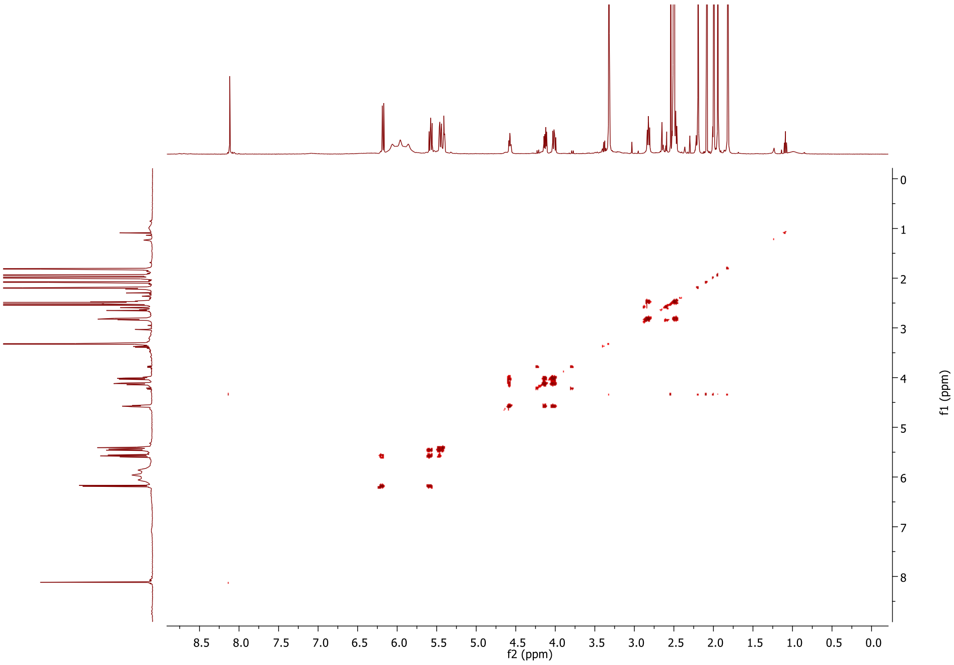

HSQC

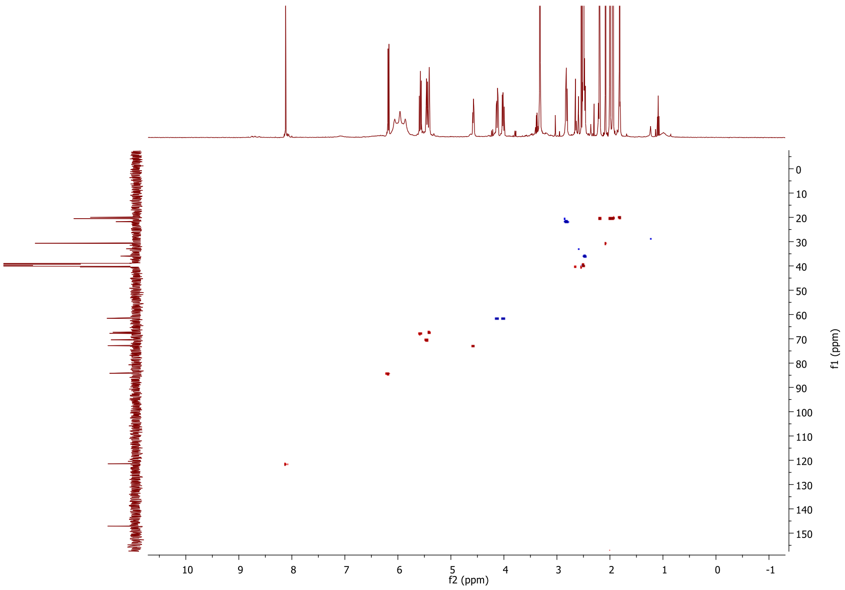

HMBC

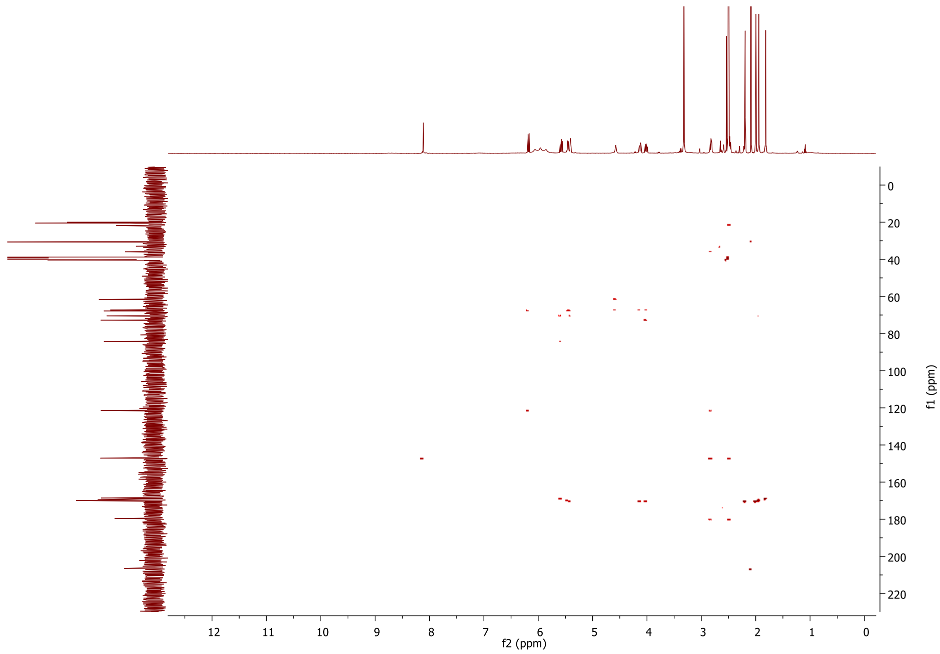

^{195}Pt NMR

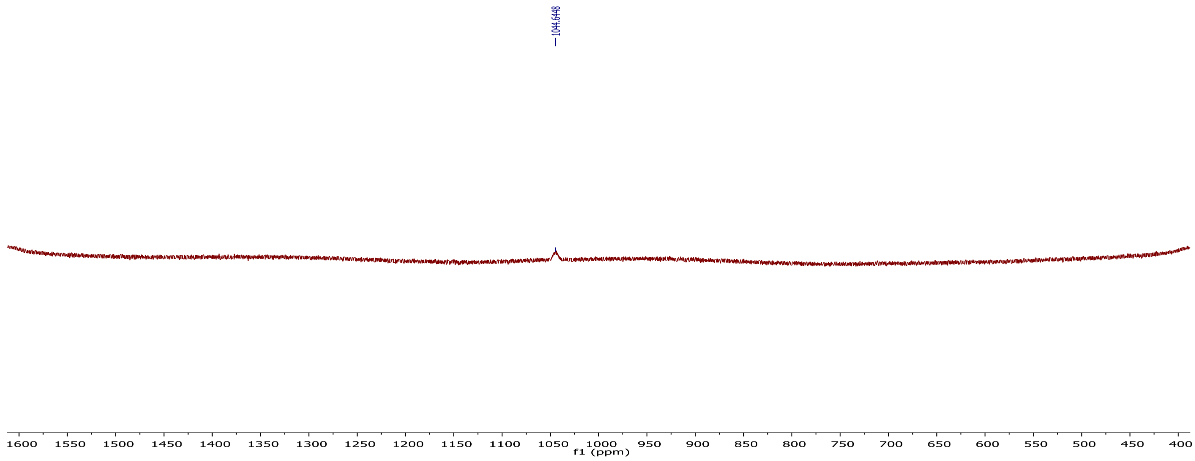

HR-MS

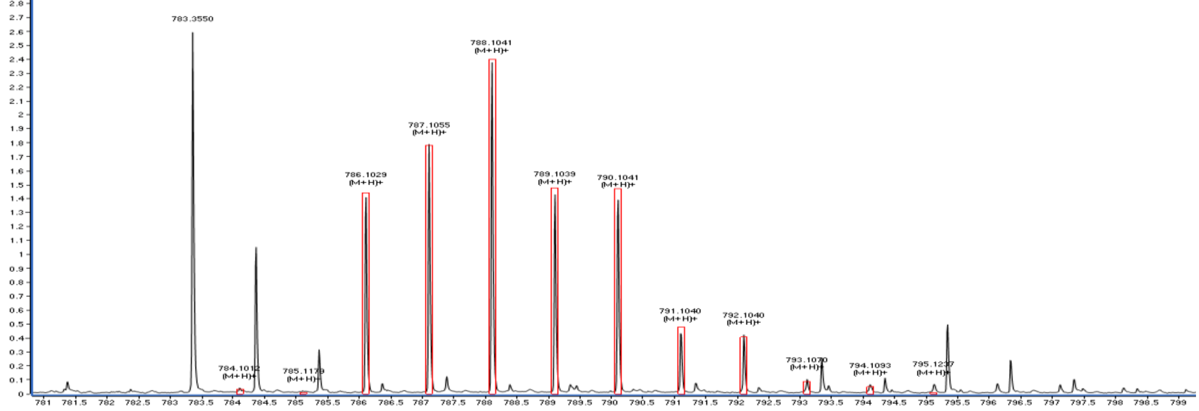

Complex 3

^{1}H NMR

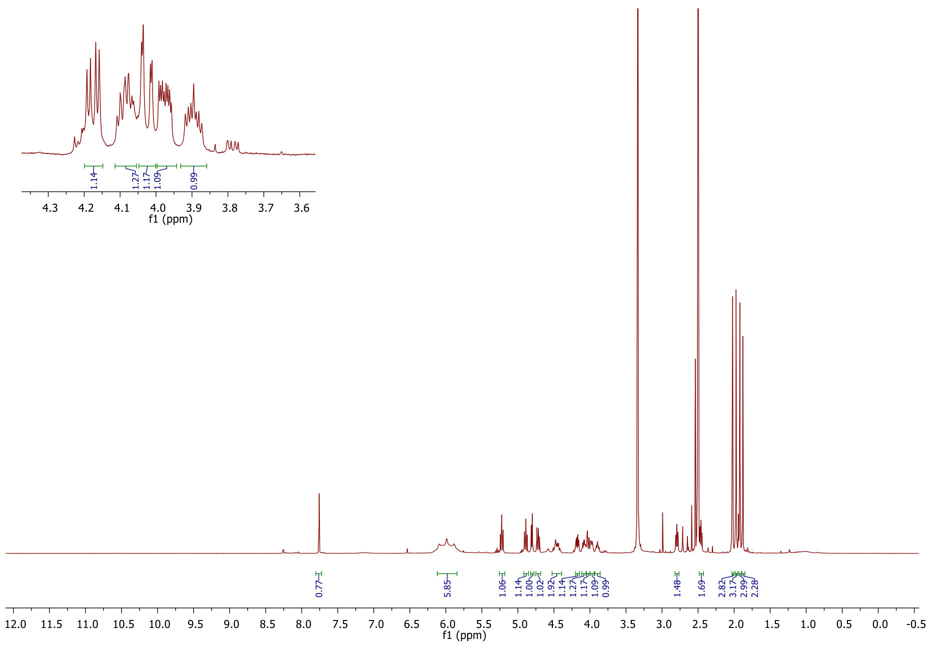

^{13}C NMR

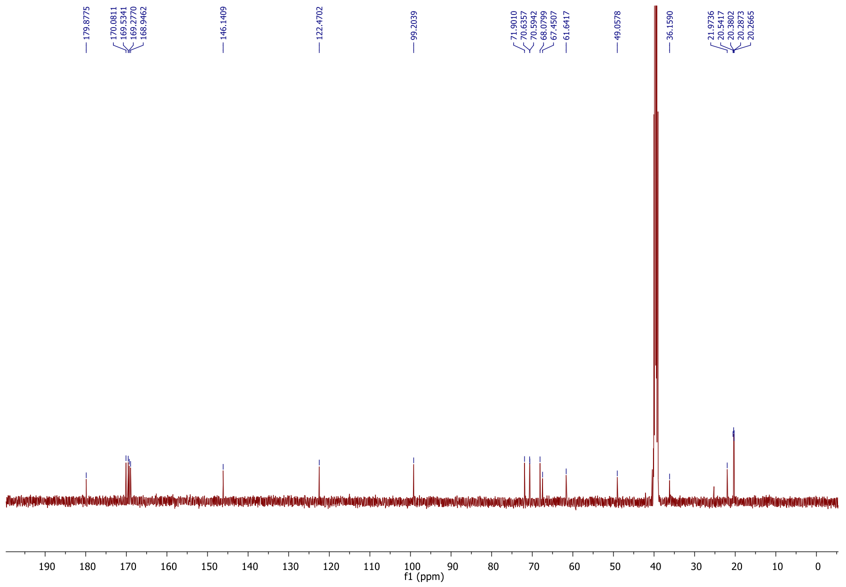

COSY

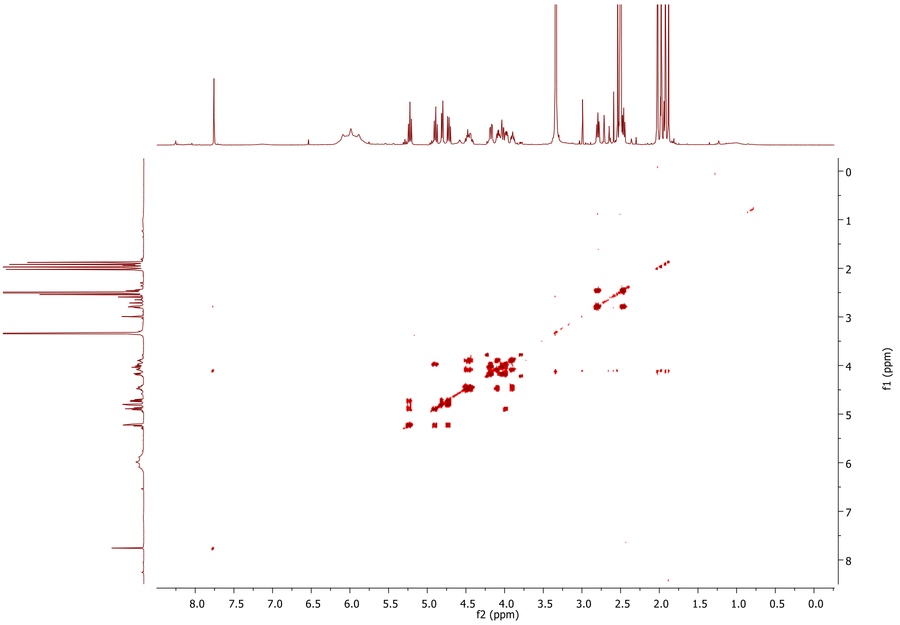

HSQC

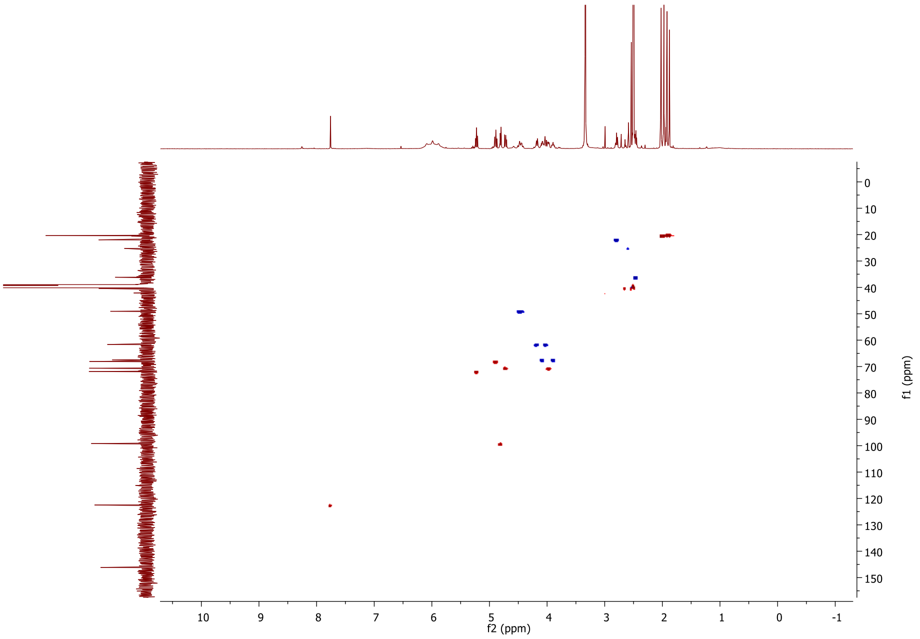

HMBC

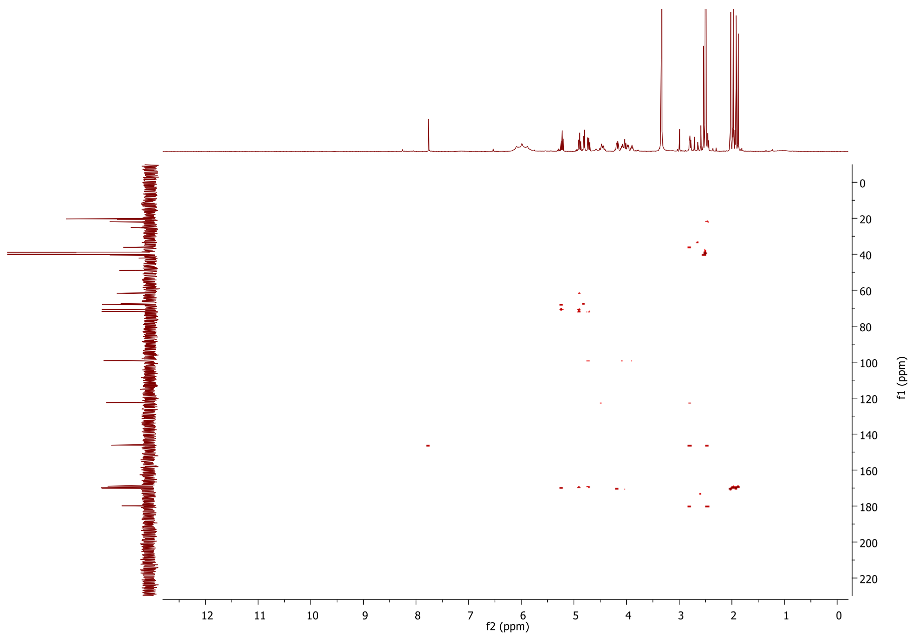

^{195}Pt NMR

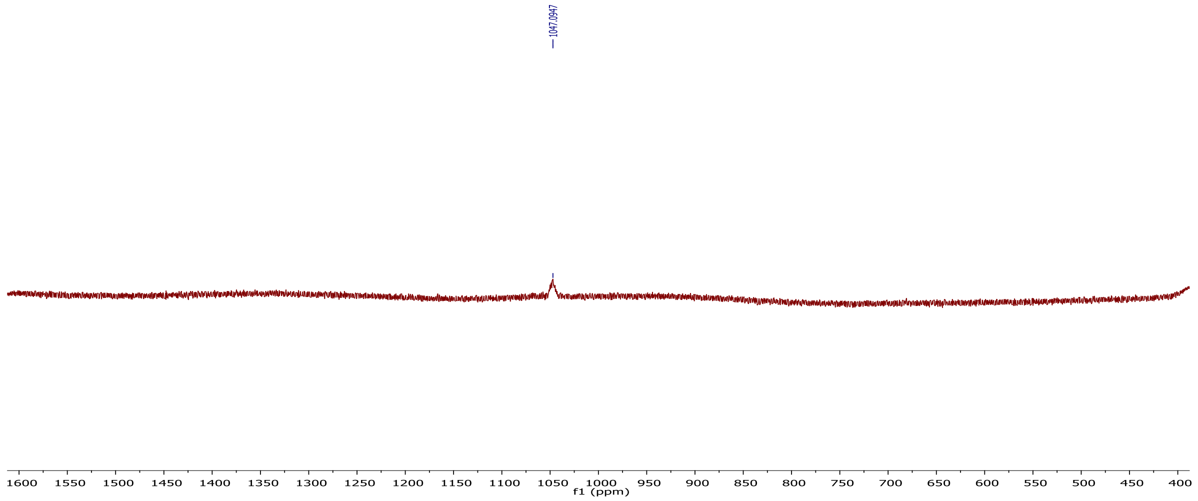

HR-MS

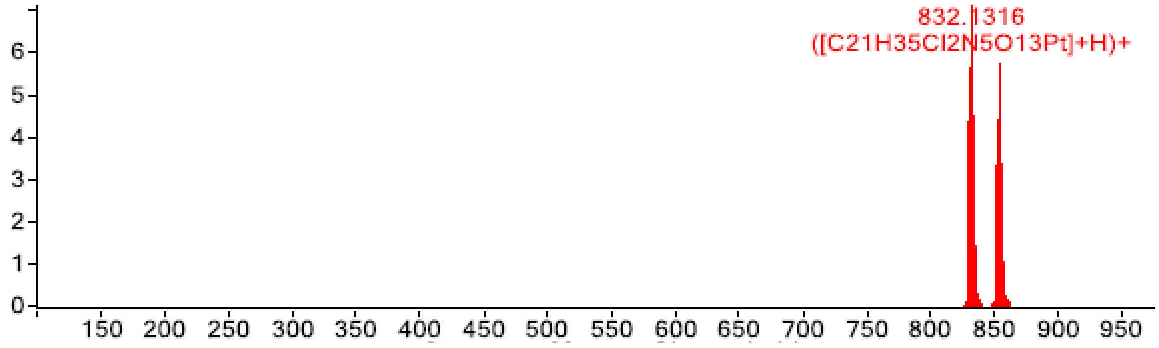

Complex 4

HPLC. Chromatograms of complex 4 in DMSO/HEPES (pH 6.8): a) t = 0 minutes; b) after one week at r.t.

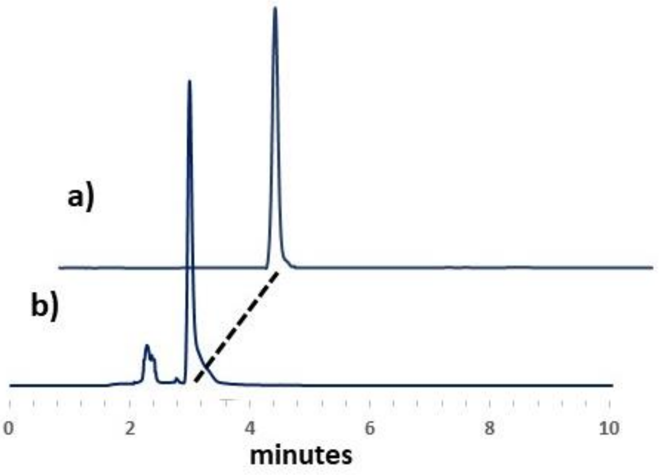

^{1}H NMR

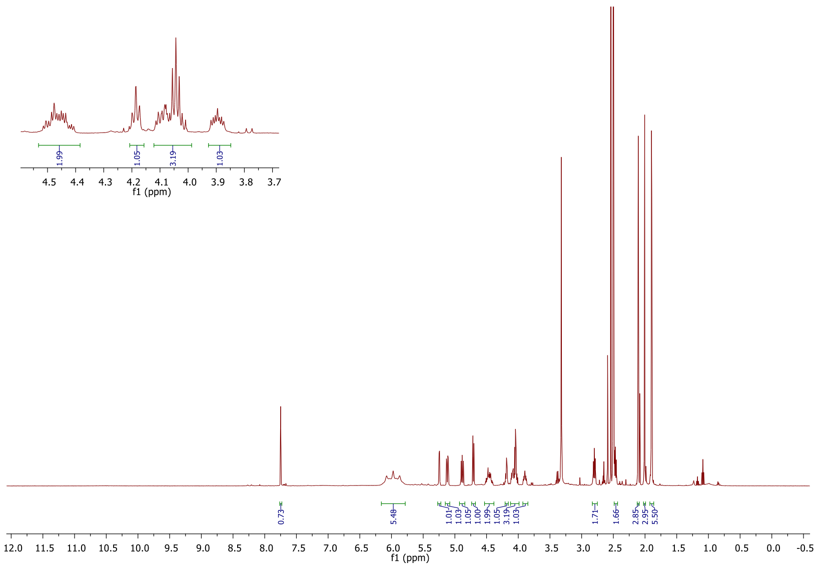

^{13}C NMR

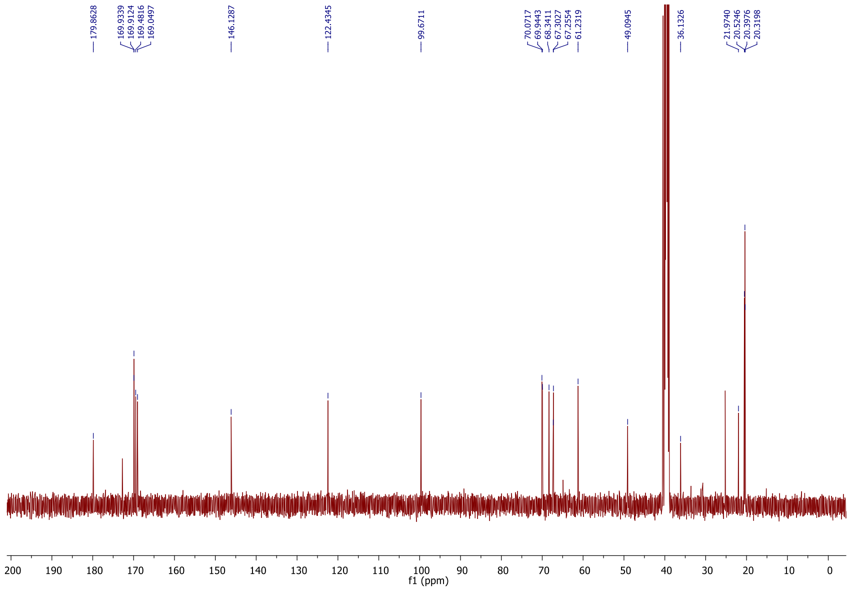

179.8628
169.9339
169.9124
169.4816
169.0497
146.1287
122.4345
99.6711
70.0717
69.9443
68.3411
67.3027
67.2554
61.2319
49.0945
36.1326
21.9740
20.5246
20.3976
20.3198
200 190 180 170 160 150 140 130 120 110 100 90 80 70 60 50 40 30 20 10 0
f1 (ppm)

COSY

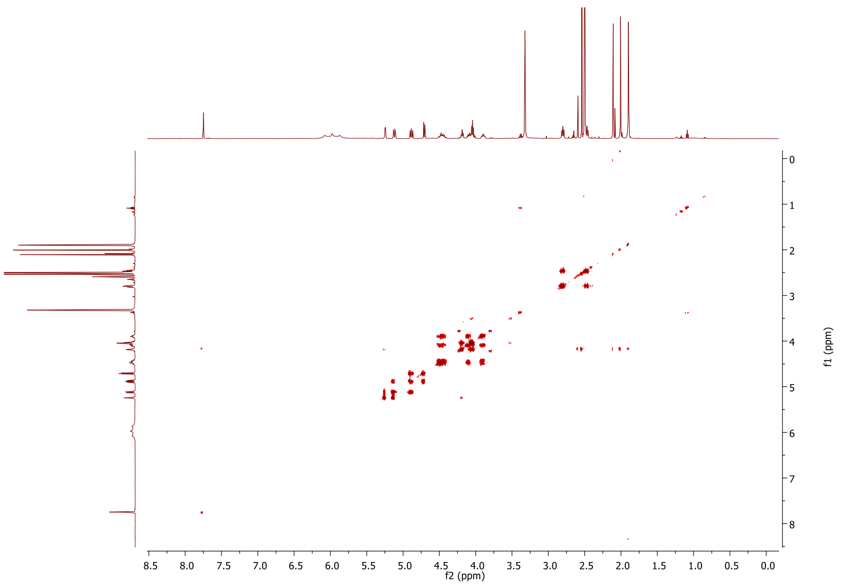

8.5 8.0 7.5 7.0 6.5 6.0 5.5 5.0 4.5 4.0 3.5 3.0 2.5 2.0 1.5 1.0 0.5 0.0
f2 (ppm)
0 1 2 3 4 5 6 7 8
f1 (ppm)

HSQC

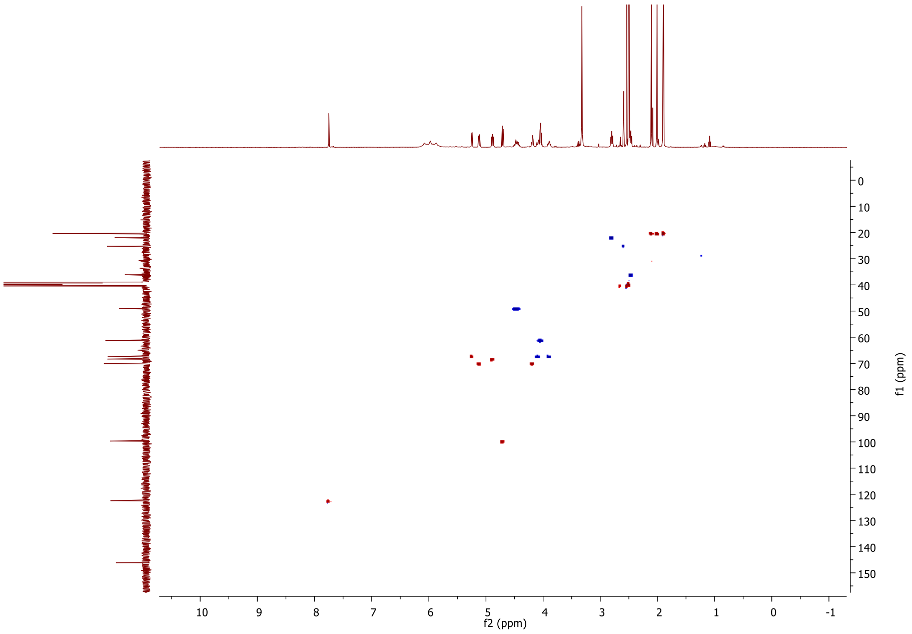

HMBC

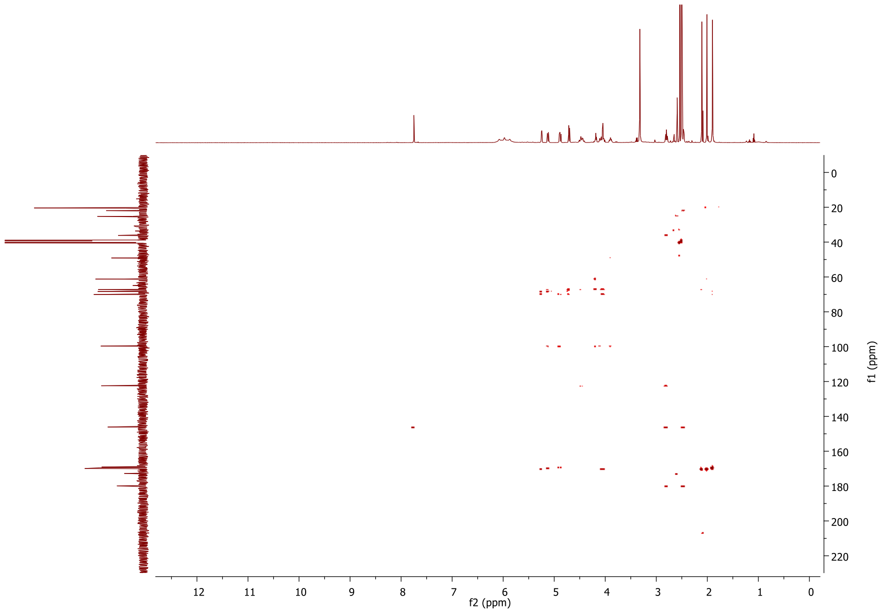

^{195}Pt NMR

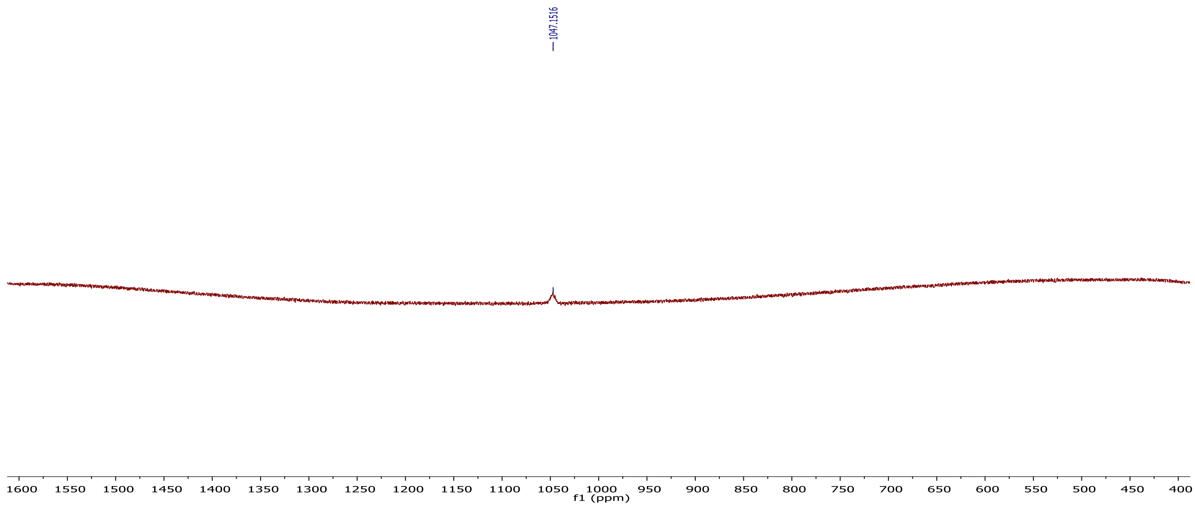

HR-MS

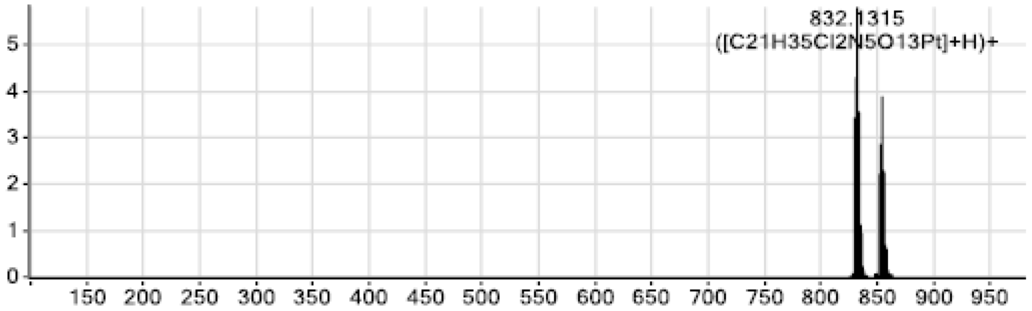

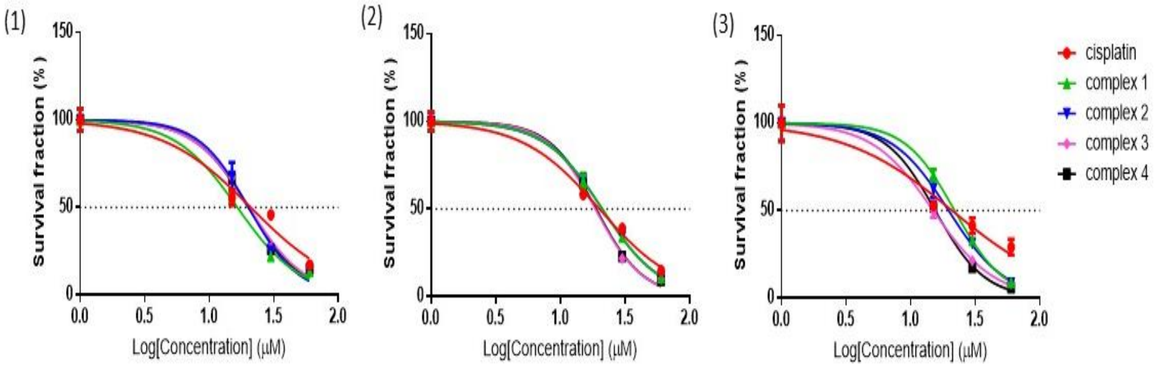

Dose-response curves used to generate IC50 for cisplatin and complexes **1-4** inhibitor activity on viability of SAOS-2 (1), U-2 OS (2) and MG63 (3) cell lines. Log[concentration] in μM and the normalized response (%) of survival fraction of cells are reported on X and Y asses, respectively. 50% survival cells is highlighted in Y dotted line. For each cell line, curves interpolation with 50% survival cells corrispond to LogIC50 of each drug. Data are reported in the graph as mean ± standard deviation. For IC50 values see table 1.
